# Deep learning applications in myocardial perfusion imaging, a systematic review and meta-analysis

**DOI:** 10.1016/j.imu.2022.101055

**Published:** 2022

**Authors:** Ebraham Alskaf, Utkarsh Dutta, Cian M. Scannell, Amedeo Chiribiri

**Affiliations:** a School of Biomedical Engineering & Imaging Sciences, King's College London, United Kingdom; b GKT, School of Medicine, King's College London, United Kingdom

**Keywords:** Deep learning, Myocardial perfusion imaging, Cardiac magnetic resonance, Coronary artery disease

## Abstract

**Background:**

Coronary artery disease (CAD) is a leading cause of death worldwide, and the diagnostic process comprises of invasive testing with coronary angiography and non-invasive imaging, in addition to history, clinical examination, and electrocardiography (ECG). A highly accurate assessment of CAD lies in perfusion imaging which is performed by myocardial perfusion scintigraphy (MPS) and magnetic resonance imaging (stress CMR). Recently deep learning has been increasingly applied on perfusion imaging for better understanding of the diagnosis, safety, and outcome of CAD.

The aim of this review is to summarise the evidence behind deep learning applications in myocardial perfusion imaging.

**Methods:**

A systematic search was performed on MEDLINE and EMBASE databases, from database inception until September 29, 2020. This included all clinical studies focusing on deep learning applications and myocardial perfusion imaging, and excluded competition conference papers, simulation and animal studies, and studies which used perfusion imaging as a variable with different focus. This was followed by review of abstracts and full texts. A meta-analysis was performed on a subgroup of studies which looked at perfusion images classification. A summary receiver-operating curve (SROC) was used to compare the performance of different models, and area under the curve (AUC) was reported. Effect size, risk of bias and heterogeneity were tested.

**Results:**

46 studies in total were identified, the majority were MPS studies (76%). The most common neural network was convolutional neural network (CNN) (41%). 13 studies (28%) looked at perfusion imaging classification using MPS, the pooled diagnostic accuracy showed AUC = 0.859. The summary receiver operating curve (SROC) comparison showed superior performance of CNN (AUC = 0.894) compared to MLP (AUC = 0.848). The funnel plot was asymmetrical, and the effect size was significantly different with p value < 0.001, indicating small studies effect and possible publication bias. There was no significant heterogeneity amongst studies according to Q test (p = 0.2184).

**Conclusion:**

Deep learning has shown promise to improve myocardial perfusion imaging diagnostic accuracy, prediction of patients’ events and safety. More research is required in clinical applications, to achieve better care for patients with known or suspected CAD.

## Highlights

• Deep learning application in cardiac imaging has become a prominent field.
• Coronary artery disease assessment with stress perfusion imaging is essential.
• Deep learning can improve imaging safety, accuracy, and predict outcome.
• Promising applications of deep learning will require clinical validation.

## 1 Introduction

### 1.1 Background

Coronary artery disease (CAD) continues to be a major cause of death and hospitalisation worldwide including in high-income countries [1]. The main underlying pathology lies in the progressive nature of coronary atherosclerotic process. Therefore, timely diagnosis to aid management of patients with CAD has significant impact on both morbidity and mortality.

There have been significant advancements in CAD imaging in the last two decades, from anatomical imaging of the coronary tree by means of invasive x-ray coronary angiography and cardiac computed tomography (CCTA), to functional assessment of coronary stenoses and their impact on the myocardium both at rest and stress (physical or pharmacological), using stress echocardiography, nuclear myocardial perfusion scanning (MPS), and stress perfusion cardiac magnetic resonance (CMR). Myocardial perfusion abnormalities are one of the early stages in the ischaemic cascade and ischaemic constellation, which also includes angina symptoms, electrocardiographic (ECG) changes and ventricular wall motion abnormalities [2].

Another exciting advancement has been made in computer vision technology following the revolution of neural networks and artificial intelligence (AI) algorithms. Deep learning is the main subfield of AI which has been the focus of computing in medical imaging, with cardiovascular imaging being one of the common arenas for such novel applications. Cardiac perfusion imaging is one of the main applications which has been studied by many deep learning practitioners and computer vision experts.

One of the key aspects of deep learning is that it allows automation of clinical tasks, and thus reduces dependence on users. This has significant advantage in perfusion imaging interpretation given that the diagnostic accuracy of visual assessment by users is highly dependent on level of training, and previously it has been demonstrated that automated quantitative analysis performed similar to highly trained users (level 3) in interpreting perfusion CMR imaging [3].

### 1.2 Rationale and objectives

There is mounting evidence of the successful applications of deep learning in cardiac perfusion imaging, as demonstrated by the increasing number of publications. Moreover, data derived from medical imaging can be integrated into specific machine learning approaches to provide valuable information for the prediction of different outcomes by exploring new correlations between variables and clinical data to build predictive models.

As a result, it is becoming increasingly important that the current literature and evidence behind deep learning applications in myocardial perfusion imaging needs further evaluation, as well as recommendations of how to fine-tune the research towards more meaningful results for patients.

Therefore, the objective of this review is to determine the diagnostic accuracy of cardiac perfusion imaging using deep learning algorithms, the impact of deep learning on image quality, image safety, and the assessment of its prognostic value.

## 2 Methods

### 2.1 Design

The umbrella protocol for this systematic review is registered in the International Prospective Register of Systematic Reviews (PROSPERO, CRD42020204164), and reported according to the Preferred Reporting Items for Systematic Reviews and Meta-Analyses (PRISMA) guidelines. This review follows the Cochrane Review structure of Diagnostic Test Accuracy (DTA) [4]. All searching activities were performed by two independent authors (EA and UD), with divergences solved after consensus.

The main review question was determined using the PICO approach:

• Population: patients with suspected or known coronary artery disease (CAD)
• Intervention: deep learning applications in CAD perfusion imaging
• Comparison: comparison with conventional CAD imaging
• Outcome: improve test accuracy and patient care

### 2.2 Selection criteria

Selection criteria decision was made by one author (EA) and over-read by a senior author (AC), with disagreement resolved after consensus. Both prospective and retrospective studies were included with no restrictions based on minimal sample sizes or recruitment process. The analysis focused on participants with known or suspected CAD who had a perfusion imaging modality with the application of deep learning. Comparison was made with the standard imaging tests used in clinical practice to identify the functional significance of coronary artery lesions (index test). A clinical reference standard is used for both techniques (reference test) which is considered the gold standard.

Medical imaging techniques presented in conferences as part of challenges, such as Medical Image Computing and Computer Assisted Intervention (MICCAI), simulation studies and animal studies were not included due to the ambiguity in their direct relation to patient care. Given that the main scope of this review is on the direct application of deep learning on myocardial perfusion imaging, studies which used perfusion data as an input variable for prediction without deep learning image applications were excluded. As there are numerous studies of left ventricular segmentation using deep learning, these studies were not included unless they form the basis for perfusion quantification. Finally, studies of automated perfusion quantification which relied mainly on hand crafted algorithms or non-deep learning algorithms such as principle component analysis (PCA) were not included.

### 2.3 Search procedure

For published literature, we searched MEDLINE (with PubMed extension) and EMBASE using Ovid search engine. To include all possible Medline Subject Headings [MeSH] terms, Yale Mesh Analyzer was used after identifying two studies manually on MEDLINE database with a focus on deep learning and CAD perfusion imaging modalities. The PMIDs for those papers were inserted into the analyser and the resulting MeSH terms were used as a guide in the systematic search. Truncation was used in imaging terms [imag*], cardiac [cardia*], myocardial [myocardia*], [quantif*] and coronary [coronar*]. Wildcards were used with one term [isch?emi*]. Plain terms were used for [‘perfusion’], [‘stress’], [‘deep learning’], [‘machine learning’], [‘neural networks’], [‘artificial intelligence’], [‘supervised learning’], [‘unsupervised learning’], and [‘semi-supervised learning’]. The search included all records from database inception until September 29, 2020 with no language constraints. Full Ovid search strategy and output is shown in Appendix [1]. No routine use of methodology search filters has been used due to reports of missing relevant studies and inconsistency [4].

To avoid publication bias and give currency to this systematic review with upcoming research, the grey literature also has been searched. This includes:

• Web of Science Conference Proceedings.
• Open Grey database.
• Manual searching of references

### 2.4 Data extraction

The extracted summary estimates included imaging modality performance after the application of deep learning (sensitivity, specificity, and area under the curve (AUC)). The sample size of each study with the imaging modality used and deep learning techniques were all reported.

The following is a summary of input data which were reported from each study in the review:

1. First author's surname
2. Year of publication
3. Model output
4. Total number of participants
5. Imaging modality used
6. Index test
7. Reference test
8. Deep learning methods
9. Sensitivity
10. Specificity
11. AUC

### 2.5 Statistical analysis

The diagnostic accuracy of the imaging modalities was measured mainly with specificity and sensitivity analyses and presented as forest plots. Data were reported as count or percentages.

Given that most studies did not report the values for true positive (TP), false positive (FP), false negative (FN), and true negative (TN), a confusion matrix was generated for each of the included studies in the meta-analysis by taking sample size (S) to calculate FN using sensitivity and FP using specificity. This was followed by subtracting FN from S to calculate TN and FP from S to calculate TP.

Although different studies reported different perfusion interpretation scales in MPS imaging, there were 2 common scales: a binary scale of normal vs abnormal, and an ordinal scale from 0 to 4. Both scaling methods are considered similar given that the ordinal scale would group 0 and 1 as normal, and 2,3 and 4 as abnormal. The ground truth finding from the reference test was considered the threshold for the summary receiver-operating curve (SROC) with bivariate diagnostic random effects meta-analysis with logit-transformed pairs of sensitivities and false positive rates method. Two SROC plots were performed using linear mixed model to compare convolutional neural network (CNN) performance against multi-layer perceptron (MLP). Publication bias and effect size derived from each study accuracy compared to mean accuracy was tested using funnel plot and Egger's test. P value of less than 0.05 was considered significant. Heterogeneity was examined using tau^2^, I^2^ and Q tests.

All statistical analysis was performed using RStudio software Version April 1, 1106 using R programming language version 4.0.4, “mada” and “meta” packages were used for meta-analysis.

## 3 Results

### 3.1 Search results

**Fig. 1:**
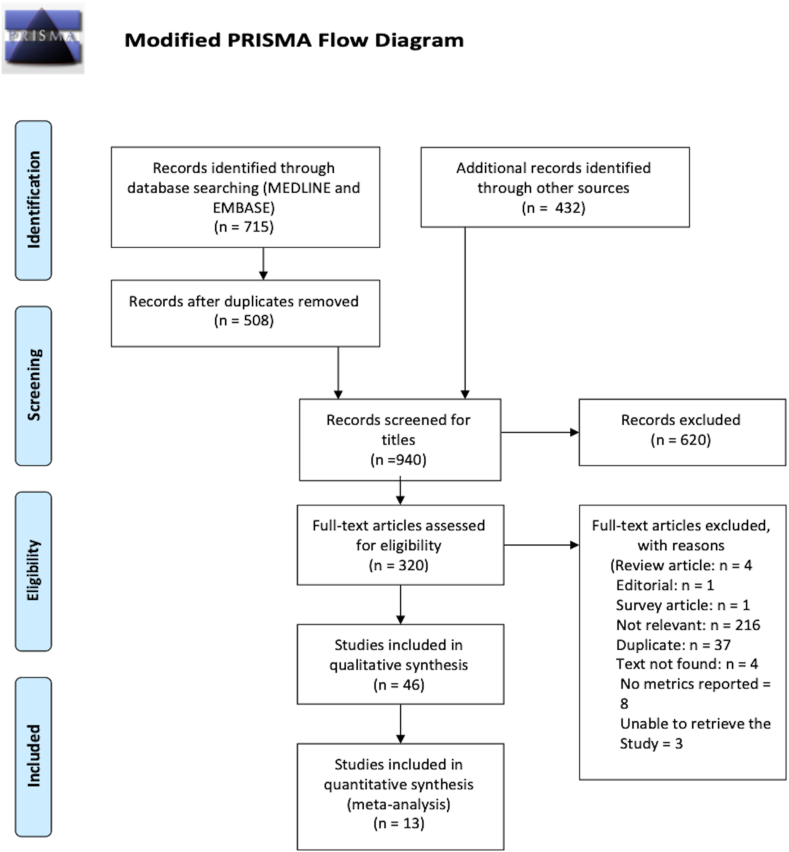
PRISMA flow diagram showing the systematic search strategy.

715 study entries from the published literature Ovid search, and 432 entries from grey literature were identified. After the screening of titles and duplicate selection, 320 studies were included in the initial analysis for which titles indicated that the study might be of relevance. Following full text review, 46 studies were included in the systematic review, of which 13 studies were included in the meta-analysis. The selection procedure and results with reasons for exclusion in the full text assessment is illustrated in Fig. 1.

### 3.2 Characteristics of studies

The final number of studies included in this systematic review was 46, details of first author, year of publication, model output, sample size, machine learning and deep learning techniques, index test (comparator), and reference test (gold standard) are all given in Table 1.

**Table 1:** List of all relevant studies included in this systematic review.

First author	Year	Model output	Sample size	Imaging modality	Model	Index test	Reference test	External validation^a^
Fujita et al. [5]	1992	Perfusion classification (8 classes)	74	MPS	MLP	Expert reader	Invasive coronary angiography	No
Wang et al. [6]	1993	Perfusion classification (2 classes x 64 segments)	100	MPS	MLP	No	Invasive coronary angiography	No
Porenta et al. [7]	1994	Perfusion classification (2 classes x 2 segments)	159	MPS	MLP	Expert reader	Invasive coronary angiography (81 cases)	No
Hamilton et al. [8]	1995	Perfusion classification (2 classes x 24 segments)	410	MPS	MLP	No	Expert reader	No
Goodenday et al. [9]	1997	Perfusion classification (2 × 3 classes)	42	MPS	MLP	Expert reader	Invasive coronary angiography	No
Lindahl et al. [10]	1997	Perfusion classification (2 classes x 2 regions)	135	MPS	MLP	Expert reader	Invasive coronary angiography	No
Scott et al. [11]	2004	Coronary artery disease prediction	102	MPS	MLP	Expert reader	Invasive coronary angiography	No
Ohlsson et al. [12]	2004	Perfusion classification (5 classes x 5 segments)	1320	MPS	MLP	No	Expert reader	No
Tagil et al. [13]	2008	Perfusion classification (2 classes x 5 segments)	316	MPS	MLP, KNN	Logistic Regression	Expert reader	No
Lomsky et al. [14]	2008	Perfusion classification (2 classes x 5 segments)	950	MPS	MLP	Emory cardiac toolbox	Expert reader	Yes
Guner et al. [15]	2010	Perfusion classification (5 classes)	308	MPS	MLP	Expert reader	Invasive coronary angiography	No
Abbasi et al. [16]	2012	Perfusion classification (2 classes x 20 segments)	208	MPS	MLP	No	Expert reader	No
Arsanjani et al. [17]	2013	Perfusion classification (5 classes x 17 segments)	957	MPS	SVM	Expert reader	Invasive coronary angiography	No
Nakajima et al. [18]	2015	Perfusion classification (5 classes x 17 segments)	1157	MPS	MLP	Expert reader	Invasive coronary angiography	Yes
Xiong et al. [19]	2015	Perfusion classification (2 classes x 17 segments)	140	CCTA	AdaBoost	Random Forest & Naïve-Bayes	Invasive coronary angiography	No
Parages et al. [20]	2016	Perfusion classification (5 classes x 17 segments)	280 simulated, 133 clinical	MPS	Naïve-Bayes	Non-prewhitening	Expert reader	na
Lee et al. [21]	2016	Ischaemia prediction from FFRCT and rCTP	250	CCTA	Gradient Boost	FFRCT alone	Invasive FFR	No
Li et al. [22]	2017	Fully automated perfusion segmentation	21	MCE	CNN + Random Forest	Active Shape Model	Expert reader	No
Kim et al. [23]	2017	Automated perfusion landmarks detection (RV insertion point and LV centre point)	59	CMR	Random Forest	Histogram of Oriented Gradients	Expert reader	No
Nakajima et al. [24]	2017	Perfusion classification (3 classes x 17 segments)	1365	MPS	MLP	No	Expert reader	No
Al Mallah et al. [25]	2017	Prediction of cardiac death at median follow-up of 4.3 years	9026	MPS	Random Forest	Logistic Regression	na	No
Nakajima et al. [26]	2018	Perfusion classification (5 classes x 17 segments)	1157	MPS	MLP	No	Expert reader	Yes
Do et al. [27]	2018	Fully automated perfusion segmentation	28	CMR	CNN	No	Expert reader	Yes
Eisenberg et al. [28]	2018	Perfusion classification (5 classes x 17 segments)	1925	MPS	LogitBoost	Expert reader	Invasive coronary angiography	No
Betancur et al. [29]	2019	Perfusion classification (2 classes x 3 segments)	1160	MPS	CNN	Automated cTPD	Invasive coronary angiography	Yes (used leave-one-center-out cross-validation)
Kim et al. [30]	2019	Fully automated perfusion quantification	145	CMR	CNN	Semi-automated	Expert reader	No
Scannell et al. [31]	2019	LV peak signal enhancement, LV bounding box and segmentation, RV insertion point	175	CMR	CNN	No	Expert reader	No
Spier et al. [32]	2019	Perfusion classification (2 classes x 17 segments)	946	MPS	CNN	No	Expert reader	No
Fan et al. [33]	2019	Accelerated k-space perfusion processing	40	CMR	CNN	Compressed sensing reconstruction	Expert reader	No
Ko et al. [34]	2019	Perfusion attenuation map generation	502	MPS	CNN	No	CT-based attenuation maps	No
Chiu et al. [35]	2019	Perfusion classification (5 classes x 17 segments)	150	MPS	CNN	Emory cardiac toolbox	Invasive coronary angiography	No
Song et al. [36]	2019	Prediction of full dose perfusion image from reduced dose	119	MPS	CNN	Spatiotemporal non-local means, Gaussian, Maximum-Likelihood	Full dose perfusion image	No
Rahmani et al. [37]	2019	Coronary angiography outcome prediction (2 classes x 20 segments)	93	MPS	MLP	No	Invasive coronary angiography	na
Singh et al. [38]	2020	Prediction of MACE at median follow-up of 385 days	1185	MPS	CNN	Clinical model, ventricular function model, absolute perfusion quantification model, integrated model	na	No
Knott et al. [39]	2020	Prediction of MACE and death at median follow-up of 605 days	1049	CMR	CNN	na	na	na
Shiri et al. [40]	2020	Prediction of full time from half time perfusion acquisition	363	MPS	CNN	na	Full time/projection acquisition perfusion	No
Ramon et al. [41]	2020	Prediction of full dose perfusion image from reduced dose	1052	MPS	CNN	na	Full dose perfusion image	No
Aposto-lopoulos et al. [42]	2020	Perfusion classification (2 classes x 17 segments)	216	MPS	CNN	Expert reader	Invasive coronary angiography	No
Xue et al. [43]	2020	Automated perfusion landmarks detection (LV blood pool enhancement)	15,789	CMR	CNN	No	Expert reader	No
Berkaya et al. [44]	2020	Perfusion quantification (3 classes x 5 sections)	192	MPS	CNN	No	Expert reader	No
Shi et al. [45]	2020	Perfusion attenuation map generation	65	MPS	CNN	na	CT-based attenuation maps	No
Hu et al. [46]	2020	Revascularisation prediction per patient/per vessel from 49 variables	1980	MPS	LogitBoost	Expert reader	Invasive coronary angiography	No
Juarez-Orozco et al. [47]	2020	Prediction of MI and death at medial follow-up of 6 years	951	MPS	CNN (Cox-Nnet)	na	Expert reader	No
Shu et al. [48]	2020	Prediction of myocardial ischaemia of MPS	154	CCTA	SVM	CTCA stenosis and radiomics signature	Expert reader	Yes
Cantoni et al. [49]	2020	Prediction of CAD from 14 variables	517	MPS (CZT-SPECT)	Random Forest	MPS (C-SPECT)	Expert reader	No
Wang et al. [50]	2020	Prediction of CAD from 5 variables	88	MPS	SVM	6 ML models (LDA, DT, KNN, LR, NB, RF)	Invasive coronary angiography	No

CMR, cardiac magnetic resonance; CNN, convolutional neural network, CT; computed tomography; CCTA, coronary CT angiography; DT, decision tree; FFR, fractional flow reserve; KNN, K-nearest neighbours; LDA, latent Dirichlet algorithm; LR, logistic regression; LV, left ventricle; MACE, major adverse cardiovascular events; MCE, myocardial contrast echocardiography; MI, myocardial infarction; MLP, multi-layer perceptron; MPS, myocardial perfusion scintigraphy; na, not applicable or not available; NB, naïve-bayes; rCTP, resting CT perfusion; RF, random forest; RV, right ventricle; SPECT, single photon-emission computed tomography; SVM, support vector machine.

a External validation by using a completely separate dataset for testing or validation outside the original training dataset.

**Fig. 2:**
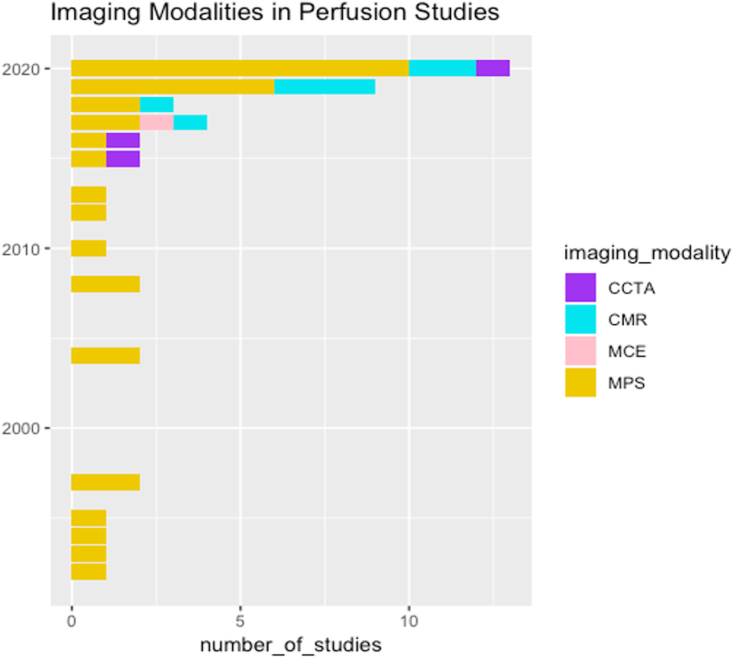
A stacked barplot showing the number of publications for each imaging modality in myocardial perfusion imaging over the last 30 years. CCTA, coronary computed tomography angiography; CMR, cardiac magnetic resonance; MCE, myocardial contrast echocardiography; MPS; myocardial perfusion scintigraphy.

The majority of the studies were performed on MPS (76%). However, the number of studies in CMR has increased in the last 2 years, as shown in Fig. 2.

**Fig. 3:**
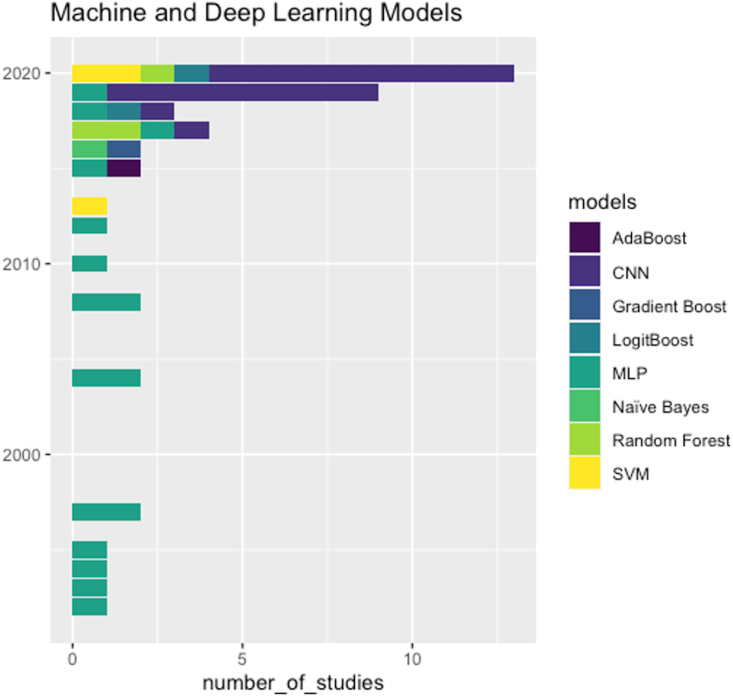
A stacked barplot showing the number of studies for each machine and deep learning algorithm used in myocardial perfusion studies over the last 30 years. CNN, convolutional neural network; MLP, multi-layer perceptron; SVM, support vector machine.

The most common neural network architecture in early years was MLP (35%), which has been dominated by CNN (41%) in recent years, as shown in Fig. 3.

### 3.3 Meta-analysis of perfusion classification

There were several studies which applied deep learning directly to segment and classify perfusion imaging maps with various classes, most of those studies were based on MPS imaging.

**Fig. 4:**
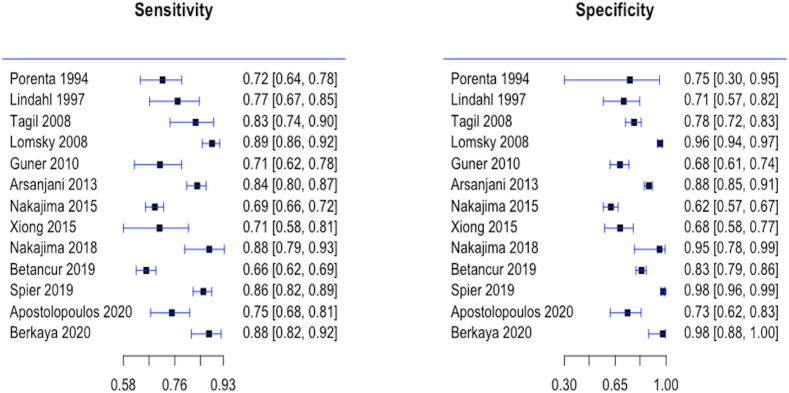
Forest plot of both specificity and sensitivity reported by the 13 MPS studies looking at deep learning in perfusion images classification.

A meta-analysis was performed on 13 studies where the output of the classifier was based on perfusion maps segmentation and referenced to the presence or absence of significant CAD based on invasive coronary angiography or consensus of expert readers of MPS, a summary of their corresponding sensitivity and specificity is depicted in the coupled forest plot Fig. 4. The plot shows good performance of the neural networks with most studies reporting sensitivity and specificity of over 65%.

**Fig. 5:**
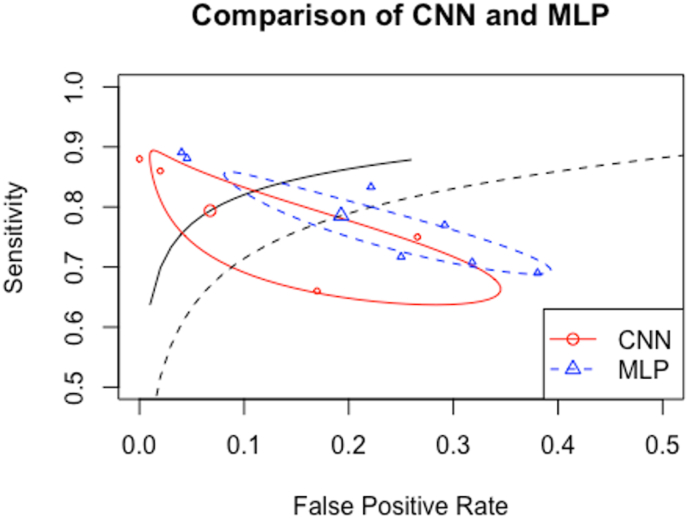
Summary receiver operating curve (SROC) comparing between convolutional neural network (CNN) and multi-layer perceptron (MLP). This shows a higher performance of CNN (solid line) compared to MLP (dotted line).

When comparing the performance of MLP with CNN across these studies using the summary receiver operating curve (SROC), CNN showed a higher value of SROC (higher sensitivity, lower false positive rate) with area under the curve (AUC) of 0.894, compared to MLP (AUC = 0.848), as showed in Fig. 5. The overall pooled AUC including all 13 studies was averaged at 0.859, showing good performance.

### 3.4 Assessment of heterogeneity

Quantifying heterogeneity showed τ^2^ = 0.0037 with confidence interval [0.0000, 0.0295], which contains zero, indicating no significant between-study heterogeneity exists in our data.

I^2^ was found to be 22.3%, meaning that less than quarter of the variation in our data is estimated to stem from true effect size differences. Using literature “rule of thumb”, we can characterize this amount of heterogeneity as mild.

Predictive interval was found to be ranging from [0.8569 to 1.1645], meaning that it is possible that some future studies will likely find positive effect based on the present evidence.

Finally, the reported p value for Q test was found to be above significance level (p = 0.2184), meaning there is no significant heterogeneity.

### 3.5 Assessment of risk of bias

The risk of bias was assessed using the Quality Assessment of Diagnostic Accuracy Studies (QUADAS) tool. A modified version was adapted and five main fields were assessed:

1. Patient selection: a high quality study would randomly select patients from a population meeting the inclusion criteria.
2. Index test: a high quality diagnostic test study would include a comparator test.
3. Reference test: all diagnostic test studies should have a gold standard test for validation.
4. Index test results blinded: a high quality study would blind the results of the comparator test to the deep learning arm.
5. Reference test results blinded: a high quality study would blind the results of the gold standard test to the deep learning arm.

Taking all the above into consideration, a table of the included studies with their associated risk of bias is shown in Appendix [2]. There were 13 studies (28%) which did not include an index test to compare with the machine learning or deep learning model before comparing with the reference test (ground truth). All studies defined a ground truth test against which they tested the model performance, and the majority of the studies blinded the model reporters from the ground truth results. This indicates the high reliability of the reported results.

**Fig. 6:**
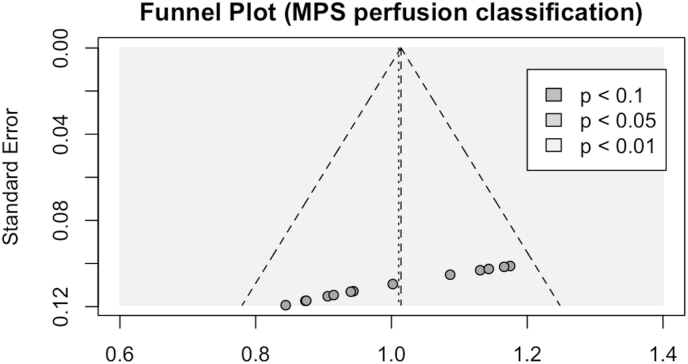
Funnel plot showing asymmetry of the studies and significant variation in their effect size values (p < 0.01).

Funnel plot in Fig. 6 shows asymmetrical pattern indicating small-study effects. Egger's test was significant with p value < 0.001, indicates that the data in the funnel plot are indeed asymmetrical, and possibly related to publication bias.

## 4 Discussion

### 4.1 Deep learning techniques

The application of deep learning models on myocardial perfusion imaging started in the early 1990s, where all the studies were focused on the use of MLP architecture and applied on MPS [[5], [6], [7], [8], [9], [10]]. MLPs are composed of three types of layers: input layers taking the raw image data, hidden layers which are connected via weight vectors, and an output layer which takes the weighted sum, applies an output function and returns a prediction [51]. The main output prediction of interest in early studies was perfusion map classification, this has continued in early 2000s, when the performance of MLP was also compared to other traditional linear and non-linear machine learning algorithms, such as K-nearest neighbours (KNN) [13] and support vector machines (SVM) [17]. Most of the networks achieved high performance metrics when compared to human expert readers.

There has been a substantial increase in the number of publications on deep learning in general with more focus on CNN over the last few years, as shown in Fig. 3. Due to the high dimensionality of imaging data, the fully connected layers which MLPs are based on put a significant limitation on the size of the model available to learn image features. The CNN overcomes this challenge by using convolutional layers which have significantly fewer parameters and make use of extensive weight sharing. The process of several convolutional layers can be thought in the following steps: detect low level features and edges from raw pixel data in the early layers, use these edges to detect shapes in the later layers, and use these shapes to detect higher-level features for prediction. An additional useful property of CNNs is that they lend themselves well to be used with transfer learning where the majority of the network is kept with its high-level feature extraction ability and only the last output layer is exchanged with a new layer to fit with the purpose of the study [51]. As a result, the majority of deep learning studies on perfusion imaging in the last few years have used CNNs as the main architecture, as shown in Fig. 3. Furthermore, the power and flexibility of CNNs has opened the window for deep learning applications in more challenging image analysis domains such as stress perfusion CMR [23,27,30,31], resting CT perfusion (rCTP), and myocardial contrast echocardiography (MCE) [22].

### 4.2 Summary of main results

The performance of neural networks for the identification of perfusion defects has proven to have a comparative performance to human expert reading, and had a strong overall accuracy in MPS studies (AUC 0.859) regardless of the comparator or reference tests. The meta-analysis presented in this review also shows the superior performance of CNNs compared to MLPs in reading and classification of perfusion maps.

The applications of deep learning on stress perfusion CMR has been increasing in recent years. There are some promising data on the effectiveness of using deep learning with CNNs to the pre-processing stage of perfusion quantification in CMR by automated identification of anatomical landmarks, such as the right ventricle (RV) insertion point into the septum and left ventricle (LV) centre on peak contrast enhancement [23,31,43]. Furthermore, CNN algorithms have been successfully applied to the segmentation of CMR perfusion images [27,30] with high performance. These applications in CMR still require further research. Another exciting application of CNN is on k-space acceleration and reconstruction for faster perfusion images acquisition [33] which is another attractive research application for deep learning. Another novel application on the horizon is using deep learning for predicting myocardial blood flow in perfusion CMR using physics-informed neural networks (PINNs) [52,53].

Other successful applications of deep learning include prediction, whether looking at prediction of death or myocardial infarction [39,47], prediction of revascularisation events [46], or prediction of high-quality full radiation dose MPS images from low dose or short time scans which has significant impact on the radiation dose delivered to the patients [36,40,41], and the identification of acquisition sequence type and image plane in CMR [54].

Furthermore, there are newer deep learning techniques using generative adversarial network (GAN) which have some promising application for image reconstruction, but this is still an active area of research.

### 4.3 Applicability of findings to review question

Given the evidence of multiple successful applications of deep learning on perfusion imaging presented in this review, the value of this evidence, although significant, remains in research applications with limited clinical use. A wider use of such applications based on the evidence presented could have significant impact on patients with known or suspected CAD. Reducing scan time, radiation dose, human resources and increasing diagnostic accuracy can save patients time, and result in better management of their coronary artery disease which has significant mortality and morbidity benefits.

## 5 Limitations

There are more applications and techniques which have been used without full publications of clinical studies and were not reported in this review, given that the main scope is clinical applications of deep learning in perfusion imaging.

The published articles included in this review did not report the same performance metrics, which was a challenge on the meta-analysis process, one of the main observations was that the performance metrics were reported in some studies for both stress and rest images, but not in others. As a result, only the highest performance score of the models for the stress images was reported in this meta-analysis.

## 6 Conclusion

### 6.1 Implications for practice

In this review the evidence of successful deep learning applications in myocardial perfusion imaging has been presented. Most of the early studies used the standard MLP perceptron architecture on MPS imaging, but more recently CNN architectures gained in popularity given its superior performance in image analysis, and deep learning applications have expanded to other perfusion imaging modalities, mainly stress perfusion CMR. The accuracy of deep learning has proven to be high in perfusion image classification to diagnose CAD compared to human readers and conventional diagnostic procedures performed in routine clinical practice, based on our meta-analysis of the relevant studies.

### 6.2 Implications for research

The successful preliminary applications of deep learning in stress perfusion CMR have opened a wide spectrum of potential applications to improve accuracy, accelerate scan times, and predict outcomes. Despite the high performance of deep learning in MPS image classification, which have shown promise for more than two decades, there is still a lack of wide use in clinical practice.

As a result, the findings of this review would encourage more clinical studies and trials to assess the performance and accuracy of deep learning in cardiac perfusion imaging using the latest techniques, in order to obtain clinical validation and to start to use this technology as clinical applications in perfusion imaging. Furthermore, other perfusion imaging modalities which are still in their infancy, such as rCTP and MCE, can also benefit from deep learning applications.

## Funding

This research received no grant from any funding agency in the public, commercial or not-for-profit sectors. This research has received grant from 10.13039/100010269Wellcome Trust [222678/Z/21/Z].

## Authors contributions

EA and UD have performed the systematic search, data extraction and writing the manuscript. EA and CS have contributed to data analysis and statistics. EA and AC have contributed to discussion and conclusion. AC has contributed to final proof-reading as a senior author. All authors have reviewed and approved the submission.

## Declaration of competing interest

The authors declare that they have no known competing financial interests or personal relationships that could have appeared to influence the work reported in this paper.
