# Supplementary material for: Deep learning applications in myocardial perfusion imaging, a systematic review and meta-analysis
Source: Inform Med Unlocked. 2022;32:101055. doi: 10.1016/j.imu.2022.101055 (PMC9514037; doi:10.1016/j.imu.2022.101055)
Supplement: Multimedia component 1 [file mmc1.pdf]

Database: Embase <1974 to 2020 Week 39>, Ovid MEDLINE(R) and Epub Ahead of Print, In-Process & Other Non-Indexed Citations and Daily <1946 to September 29, 2020>
Search Strategy:
----
1 machine learning.mp. [mp=ti, ab, hw, tn, ot, dm, mf, dv, kw, fx, dq, nm, kf, ox, px, rx, an, ui, sy] (89952)
2 deep learning.mp. [mp=ti, ab, hw, tn, ot, dm, mf, dv, kw, fx, dq, nm, kf, ox, px, rx, an, ui, sy] (27628)
3 neural network.mp. [mp=ti, ab, hw, tn, ot, dm, mf, dv, kw, fx, dq, nm, kf, ox, px, rx, an, ui, sy] (98463)
4 artificial intelligence.mp. [mp=ti, ab, hw, tn, ot, dm, mf, dv, kw, fx, dq, nm, kf, ox, px, rx, an, ui, sy] (59934)
5 supervised learning.mp. [mp=ti, ab, hw, tn, ot, dm, mf, dv, kw, fx, dq, nm, kf, ox, px, rx, an, ui, sy] (6006)
6 unsupervised learning.mp. [mp=ti, ab, hw, tn, ot, dm, mf, dv, kw, fx, dq, nm, kf, ox, px, rx, an, ui, sy] (3051)
7 semi-supervised learning.mp. [mp=ti, ab, hw, tn, ot, dm, mf, dv, kw, fx, dq, nm, kf, ox, px, rx, an, ui, sy] (1098)
8 1 or 2 or 3 or 4 or 5 or 6 or 7 (230496)
9 coronar*.mp. [mp=ti, ab, hw, tn, ot, dm, mf, dv, kw, fx, dq, nm, kf, ox, px, rx, an, ui, sy] (1227438)
10 cardia*.mp. [mp=ti, ab, hw, tn, ot, dm, mf, dv, kw, fx, dq, nm, kf, ox, px, rx, an, ui, sy] (1768037)
11 myocardia*.mp. [mp=ti, ab, hw, tn, ot, dm, mf, dv, kw, fx, dq, nm, kf, ox, px, rx, an, ui, sy] (954843)
12 stress.mp. [mp=ti, ab, hw, tn, ot, dm, mf, dv, kw, fx, dq, nm, kf, ox, px, rx, an, ui, sy] (2220009)
13 heart.mp. [mp=ti, ab, hw, tn, ot, dm, mf, dv, kw, fx, dq, nm, kf, ox, px, rx, an, ui, sy] (3670378)
14 9 or 10 or 11 or 12 or 13 (6668079)
15 perfusion.mp. [mp=ti, ab, hw, tn, ot, dm, mf, dv, kw, fx, dq, nm, kf, ox, px, rx, an, ui, sy] (475568)
16 14 and 15 (172843)
17 8 and 16 (467)
18 perfusion quantif*.mp. [mp=ti, ab, hw, tn, ot, dm, mf, dv, kw, fx, dq, nm, kf, ox, px, rx, an, ui, sy] (637)
19 14 and 18 (261)
20 17 or 19 (715)
21 remove duplicates from 20 (508)

****

1.

Classification of Polar Maps from Cardiac Perfusion Imaging with Graph-Convolutional Neural Networks.
Spier N., Nekolla S., Rupprecht C., Mustafa M., Navab N., Baust M.
Embase
Scientific reports. 9 (1) (pp 7569), 2019. Date of Publication: 20 May 2019.
[Article]
AN: 627922840
Publisher

NLM (Medline)

Link to the Ovid Full Text or citation:
Click here for full text options

Link to the External Link Resolver:
SFX Link

2.

Positron emission tomography imaging in cardiovascular disease.
Tarkin J.M., A orovic A., Wall C., Gopalan D., Rudd J.H.F.
Embase
Heart. (no pagination), 2020. Article Number: 315183. Date of Publication: 2020.
[Review]
AN: 632892148
Publisher
BMJ Publishing Group (E-mail: support@bmj.com)

Link to the Ovid Full Text or citation:
Click here for full text options

Link to the External Link Resolver:
SFX Link

3.

The sub-millisievert era in CTCA: the technical basis of the new radiation dose approach.
Schicchi N., Fogante M., Palumbo P., Agliata G., Esposto Pirani P., Di Cesare E., Giovagnoni A.
Embase
La Radiologia medica. (no pagination), 2020. Date of Publication: 15 Sep 2020.
[Review]
AN: 632881858
Publisher
NLM (Medline)

Link to the Ovid Full Text or citation:
Click here for full text options

Link to the External Link Resolver:
SFX Link

4.

Improving Diagnostic Accuracy in Low-Dose SPECT Myocardial Perfusion Imaging with Convolutional Denoising Networks.
Ramon A.J., Yang Y., Pretorius P.H., Johnson K.L., King M.A., Wernick M.N.
Embase
IEEE Transactions on Medical Imaging. 39 (9) (pp 2893-2903), 2020. Article Number: 9031353. Date of Publication: September 2020.
[Article]
AN: 632735868
Publisher
Institute of Electrical and Electronics Engineers Inc.

Link to the Ovid Full Text or citation:
Click here for full text options

Link to the External Link Resolver:
SFX Link

5.

AI and Machine Learning in Nuclear Medicine: Future Perspectives.
Seifert R., Weber M., Kocakavuk E., Rischpler C., Kersting D.
Embase
Seminars in Nuclear Medicine. (no pagination), 2020. Date of Publication: 2020.
[Review]
AN: 2007818651
Publisher
W.B. Saunders

Link to the Ovid Full Text or citation:
Click here for full text options

Link to the External Link Resolver:
SFX Link

6.

Pulmonary function evaluation based on time-series analysis of radiographic lung density: A preliminary study.
Tanaka R., Matsumoto I., Tamura M., Takata M., Kasahara K., Ohkura N., Inoue D., Matsuura Y.
Embase
International Journal of Computer Assisted Radiology and Surgery. Conference: 34th International Congress and Exhibition of the Computer Assisted Radiology and Surgery, CARS 2020. Germany. 15 (1 Supplement) (pp S156-S157), 2020. Date of Publication: 2020.
[Conference Abstract]
AN: 632801711
Publisher
Springer

Link to the Ovid Full Text or citation:
[Click here for full text options]

Link to the External Link Resolver:
[SFX Link]

7.

Impact of the Arterial Input Sampling Location on CMR First-Pass Myocardial Perfusion Quantification.
Franks R., Milidonis X., Schneider T., Sanchez-Gonzalez J., Plein S., Chiribiri A.
Embase
JACC: Cardiovascular Imaging. (no pagination), 2020. Date of Publication: 2020.
[Letter]
AN: 2007794488
Publisher
Elsevier Inc. (E-mail: usjcs@elsevier.com)

Link to the Ovid Full Text or citation:
[Click here for full text options]

Link to the External Link Resolver:
[SFX Link]

8.

Functional cardiac CT-Going beyond Anatomical Evaluation of Coronary Artery Disease with Cine CT, CT-FFR, CT Perfusion and Machine Learning.
Peper J., Sucha D., Swaans M., Leiner T.
Embase
The British journal of radiology. 93 (1113) (pp 20200349), 2020. Date of Publication: 01 Sep 2020.
[Review]
AN: 632594662
Publisher
NLM (Medline)

Link to the Ovid Full Text or citation:
[Click here for full text options]

Link to the External Link Resolver:
[SFX Link]

9.

Applications of artificial intelligence in multimodality cardiovascular imaging: A state-

of-the-art review.
Xu B., Kocyigit D., Grimm R., Griffin B.P., Cheng F.
Embase
Progress in Cardiovascular Diseases. 63 (3) (pp 367-376), 2020. Date of Publication: May - June 2020.
[Review]
AN: 2005439672
Publisher
W.B. Saunders

Link to the Ovid Full Text or citation:
Click here for full text options

Link to the External Link Resolver:
SFX Link

10.

The expanding potential of functional liver imaging: From research tools to clinical practice in oncology and internal medicine.
Piscaglia F., Marcelli E.
Embase
European Journal of Internal Medicine. 79 (pp 23-24), 2020. Date of Publication: September 2020.
[Note]
AN: 2007064065
Publisher
Elsevier B.V.

Link to the Ovid Full Text or citation:
Click here for full text options

Link to the External Link Resolver:
SFX Link

11.

Automatic characterization of myocardial perfusion imaging polar maps employing deep learning and data augmentation.
Apostolopoulos I.D., Papathanasiou N.D., Spyridonidis T., Apostolopoulos D.J.
Embase
Hellenic journal of nuclear medicine. 23 (2) (pp 125-132), 2020. Date of Publication: 01 May 2020.
[Article]
AN: 632461708
Publisher
NLM (Medline)

Link to the Ovid Full Text or citation:

Click here for full text options

Link to the External Link Resolver:
SFX Link

12.

Quantification of Myocardial Blood Flow by Machine Learning Analysis of Modified Dual Bolus MRI Examination.
Husso M., Afara I.O., Nissi M.J., Kuivanen A., Halonen P., Tarkia M., Teuho J., Saunavaara V., Vainio P., Sipola P., Manninen H., Yla-Herttuala S., Knuuti J., Toyras J.
Embase
Annals of Biomedical Engineering. (no pagination), 2020. Date of Publication: 2020.
[Article]
AN: 2005920920
Publisher
Springer

Link to the Ovid Full Text or citation:
Click here for full text options

Link to the External Link Resolver:
SFX Link

13.

Software reproducibility of myocardial blood flow and flow reserve quantification in ischemic heart disease: A 13N-ammonia PET study.
Monroy-Gonzalez A.G., Juarez-Orozco L.E., Han C., Vedder I.R., Garcia D.V., Borra R., Slomka P.J., Nesterov S.V., Knuuti J., Slart R.H.J.A., Alexanderson-Rosas E.
Embase
Journal of Nuclear Cardiology. 27 (4) (pp 1225-1233), 2020. Date of Publication: 01 Aug 2020.
[Article]
AN: 2003386415
Publisher
Springer

Link to the Ovid Full Text or citation:
Click here for full text options

Link to the External Link Resolver:
SFX Link

14.

Preface.
Miller F.H.
Embase
Advances in Clinical Radiology. 2 (pp xvii-xviii), 2020. Date of Publication: September 2020.
[Editorial]
AN: 2007433115
Publisher
Elsevier Inc. (E-mail: usjcs@elsevier.com)

Link to the Ovid Full Text or citation:
Click here for full text options

Link to the External Link Resolver:
SFX Link

15.

Automated detection of left ventricle in arterial input function images for inline perfusion mapping using deep learning: A study of 15,000 patients.
Xue H., Tseng E., Knott K.D., Kotecha T., Brown L., Plein S., Fontana M., Moon J.C., Kellman P.
Embase
Magnetic Resonance in Medicine. 84 (5) (pp 2788-2800), 2020. Date of Publication: 01 Nov 2020.
[Article]
AN: 2004853592
Publisher
John Wiley and Sons Inc. (P.O.Box 18667, Newark NJ 07191-8667, United States)

Link to the Ovid Full Text or citation:
Click here for full text options

Link to the External Link Resolver:
SFX Link

16.

Classification models for SPECT myocardial perfusion imaging.
Kaplan Berkaya S., Ak Sivrikoz I., Gunal S.
Embase
Computers in Biology and Medicine. 123 (no pagination), 2020. Article Number: 103893. Date of Publication: August 2020.
[Article]
AN: 2007121460
Publisher
Elsevier Ltd

Link to the Ovid Full Text or citation:

Click here for full text options

Link to the External Link Resolver:
SFX Link

17.

PET Imaging of Tumor Perfusion - A Potential Cancer Biomarker?.
Johnson G.B., Harms H.J., Johnson D.R., Jacobson M.S.
Embase
Seminars in Nuclear Medicine. (no pagination), 2020. Date of Publication: 2020.
[Review]
AN: 2007388554
Publisher
W.B. Saunders

Link to the Ovid Full Text or citation:
Click here for full text options

Link to the External Link Resolver:
SFX Link

18.

Cardiovascular CT and MRI in 2019: Review of Key Articles.
Dodd J.D., Leipsic J.
Embase
Radiology. (pp 200605), 2020. Date of Publication: 04 Aug 2020.
[Article]
AN: 632526951
Publisher
NLM (Medline)

Link to the Ovid Full Text or citation:
Click here for full text options

Link to the External Link Resolver:
SFX Link

19.

Compressed Optoacoustic Sensing of Volumetric Cardiac Motion.
Ozbek A., Dean-Ben X.L., Razansky D.
Embase
IEEE transactions on medical imaging. PP (no pagination), 2020. Date of Publication: 28 May 2020.
[Article]

AN: 632522332
Publisher
NLM (Medline)

Link to the Ovid Full Text or citation:
[Click here for full text options]

Link to the External Link Resolver:
[SFX Link]

20.

Application and Translation of Artificial Intelligence to Cardiovascular Imaging in Nuclear Medicine and Noncontrast CT.
Slomka P.J., Miller R.J., Isgum I., Dey D.
Embase
Seminars in Nuclear Medicine. 50 (4) (pp 357-366), 2020. Date of Publication: July 2020.
[Review]
AN: 2005950120
Publisher
W.B. Saunders

Link to the Ovid Full Text or citation:
[Click here for full text options]

Link to the External Link Resolver:
[SFX Link]

21.

Machine learning predicts per-vessel early coronary revascularization after fast myocardial perfusion SPECT: Results from multicentre REFINE SPECT registry.
Hu L.-H., Betancur J., Sharir T., Einstein A.J., Bokhari S., Fish M.B., Ruddy T.D., Kaufmann P.A., Sinusas A.J., Miller E.J., Bateman T.M., Dorbala S., Di Carli M., Germano G., Commandeur F., Liang J.X., Otaki Y., Tamarappoo B.K., Dey D., Berman D.S., Slomka P.J.
Embase
European Heart Journal Cardiovascular Imaging. 21 (5) (pp 549-559), 2020. Date of Publication: 01 May 2020.
[Article]
AN: 632134799
Publisher
Oxford University Press

Link to the Ovid Full Text or citation:
[Click here for full text options]

Link to the External Link Resolver:

SFX Link

22.

Deep learning-based attenuation map generation for myocardial perfusion SPECT.
Shi L., Onofrey J.A., Liu H., Liu Y.-H., Liu C.
Embase
European Journal of Nuclear Medicine and Molecular Imaging. 47 (10) (pp 2383-2395), 2020. Date of Publication: 01 Sep 2020.
[Article]
AN: 2004579936
Publisher
Springer

Link to the Ovid Full Text or citation:
Click here for full text options

Link to the External Link Resolver:
SFX Link

23.

Late gadolinium uptake demonstrated with magnetic resonance in patients where automated PERFIT analysis of myocardial SPECT suggests irreversible perfusion defect.
Rosendahl L., Blomstrand P., Ohlsson J.L., Bjorklund P.G., Ahlander B.M., Starck S.A., Engvall J.E.
Embase
BMC medical imaging. 8 (pp 17), 2008. Article Number: 17. Date of Publication: 2008.
[Article]
AN: 354410567

Link to the Ovid Full Text or citation:
Click here for full text options

Link to the External Link Resolver:
SFX Link

24.

Coronary arteries hemodynamics: effect of arterial geometry on hemodynamic parameters causing atherosclerosis.
Wong K.K.L., Wu J., Liu G., Huang W., Ghista D.N.
Embase
Medical and Biological Engineering and Computing. 58 (8) (pp 1831-1843), 2020. Date of Publication: 01 Aug 2020.

[Article]
AN: 2005188106
Publisher
Springer

Link to the Ovid Full Text or citation:
Click here for full text options

Link to the External Link Resolver:
SFX Link

25.

Recent and upcoming technological developments in computed tomography: High speed, low dose, deep learning, multienergy.
Lell M.M., Kachelriess M.
Embase
Investigative Radiology. 55 (1) (pp 8-19), 2020. Date of Publication: 01 Jan 2020.
[Review]
AN: 631013484
Publisher
Lippincott Williams and Wilkins (E-mail: kathiest.clai@apta.org)

Link to the Ovid Full Text or citation:
Click here for full text options

Link to the External Link Resolver:
SFX Link

26.

Left ventricle segmentation in the era of deep learning.
Wolterink J.M.
Embase
Journal of Nuclear Cardiology. 27 (3) (pp 988-991), 2020. Date of Publication: 01 Jun 2020.
[Editorial]
AN: 626707426
Publisher
Springer

Link to the Ovid Full Text or citation:
Click here for full text options

Link to the External Link Resolver:
SFX Link

27.

Rationale and design of the REgistry of Fast Myocardial Perfusion Imaging with NExt generation SPECT (REFINE SPECT).
Slomka P.J., Betancur J., Liang J.X., Otaki Y., Hu L.-H., Sharir T., Dorbala S., Di Carli M., Fish M.B., Ruddy T.D., Bateman T.M., Einstein A.J., Kaufmann P.A., Miller E.J., Sinusas A.J., Azadani P.N., Gransar H., Tamarappoo B.K., Dey D., Berman D.S., Germano G.
Embase
Journal of Nuclear Cardiology. 27 (3) (pp 1010-1021), 2020. Date of Publication: 01 Jun 2020.
[Article]
AN: 622668113
Publisher
Springer

Link to the Ovid Full Text or citation:
Click here for full text options

Link to the External Link Resolver:
SFX Link

28.

A learning-based automatic segmentation and quantification method on left ventricle in gated myocardial perfusion SPECT imaging: A feasibility study.
Wang T., Lei Y., Tang H., He Z., Castillo R., Wang C., Li D., Higgins K., Liu T., Curran W.J., Zhou W., Yang X.
Embase
Journal of Nuclear Cardiology. 27 (3) (pp 976-987), 2020. Date of Publication: 01 Jun 2020.
[Article]
AN: 626255686
Publisher
Springer

Link to the Ovid Full Text or citation:
Click here for full text options

Link to the External Link Resolver:
SFX Link

29.

Impact of machine-learning CT-derived fractional flow reserve for the diagnosis and management of coronary artery disease in the randomized CRESCENT trials.
Nous F.M.A., Budde R.P.J., Lubbers M.M., Yamasaki Y., Kardys I., Bruning T.A., Akkerhuis J.M., Kofflard M.J.M., Kietselaer B., Galema T.W., Nieman K.
Embase

European Radiology. 30 (7) (pp 3692-3701), 2020. Date of Publication: 01 Jul 2020.
[Article]
AN: 2004450501
Publisher
Springer

Link to the Ovid Full Text or citation:
Click here for full text options

Link to the External Link Resolver:
SFX Link

30.

Predicting Chronic Myocardial Ischemia Using CCTA-Based Radiomics Machine Learning Nomogram.
Shu Z.-Y., Cui S.-J., Zhang Y.-Q., Xu Y.-Y., Hung S.-C., Fu L.-P., Pang P.-P., Gong X.-Y., Jin Q.-Y.
Embase
Journal of Nuclear Cardiology. (no pagination), 2020. Date of Publication: 2020.
[Article]
AN: 2005272248
Publisher
Springer

Link to the Ovid Full Text or citation:
Click here for full text options

Link to the External Link Resolver:
SFX Link

31.

A machine learning-based approach to directly compare the diagnostic accuracy of myocardial perfusion imaging by conventional and cadmium-zinc telluride SPECT.
Cantoni V., Green R., Ricciardi C., Assante R., Zampella E., Nappi C., Gaudieri V., Mannarino T., Genova A., De Simini G., Giordano A., D'Antonio A., Acampa W., Petretta M., Cuocolo A.
Embase
Journal of Nuclear Cardiology. (no pagination), 2020. Date of Publication: 2020.
[Article]
AN: 2005101544
Publisher
Springer

Link to the Ovid Full Text or citation:
Click here for full text options

Link to the External Link Resolver:

SFX Link

32.

Evaluation of the diagnostic value of joint PET myocardial perfusion and metabolic imaging for vascular stenosis in patients with obstructive coronary artery disease.
Wang F., Xu W., Lv W., Du D., Feng H., Zhang X., Wang S., Chen W., Lu L.
Embase
Journal of Nuclear Cardiology. (no pagination), 2020. Date of Publication: 2020.
[Article]
AN: 2005014180
Publisher
Springer

Link to the Ovid Full Text or citation:
Click here for full text options

Link to the External Link Resolver:
SFX Link

33.

Deep-Learning-Based Preprocessing for Quantitative Myocardial Perfusion MRI.
Scannell C.M., Veta M., Villa A.D.M., Sammut E.C., Lee J., Breeuwer M., Chiribiri A.
Embase
Journal of Magnetic Resonance Imaging. 51 (6) (pp 1689-1696), 2020. Date of Publication: 01 Jun 2020.
[Article]
AN: 2003606699
Publisher
John Wiley and Sons Inc. (P.O.Box 18667, Newark NJ 07191-8667, United States)

Link to the Ovid Full Text or citation:
Click here for full text options

Link to the External Link Resolver:
SFX Link

34.

The use of HPI (Hypotension probability indicator) during major intracranial surgery; preliminary results of a prospective randomized trial.
Pouska J., Cerveny V., Zatloukal J., Kletecka J., Bene J.
Embase
Intensive Care Medicine Experimental. Conference: 32nd European Society of Intensive Care Medicine Annual Congress, ESICM 2019. Germany. 7 (Supplement 3) (no pagination), 2019. Date of Publication: September 2019.

[Conference Abstract]
AN: 631808551
Publisher
Springer

Link to the Ovid Full Text or citation:
Click here for full text options

Link to the External Link Resolver:
SFX Link

35.

Predicting donor heart function in a heartbeat.
Keller S.P.
Embase
Science Translational Medicine. 12 (538) (no pagination), 2020. Article Number: eabb5667. Date of Publication: 08 Apr 2020.
[Article]
AN: 2005682241
Publisher
American Association for the Advancement of Science

Link to the Ovid Full Text or citation:
Click here for full text options

Link to the External Link Resolver:
SFX Link

36.

Computed tomographic evaluation of pancreatic perfusion in healthy dogs.
Kloer T.B., Rao S., Twedt D.C., Marolf A.J.
Embase
American Journal of Veterinary Research. 81 (2) (pp 131-138), 2020. Date of Publication: February 2020.
[Article]
AN: 2003705586
Publisher
American Veterinary Medical Association (E-mail: avmainfo@avma.org)

Link to the Ovid Full Text or citation:
Click here for full text options

Link to the External Link Resolver:
SFX Link

37.

Standard SPECT myocardial perfusion estimation from half-time acquisitions using deep convolutional residual neural networks.
Shiri I., AmirMozafari Sabet K., Arabi H., Pourkeshavarz M., Teimourian B., Ay M.R., Zaidi H.
Embase
Journal of Nuclear Cardiology. (no pagination), 2020. Date of Publication: 2020.
[Article]
AN: 2004804723
Publisher
Springer

Link to the Ovid Full Text or citation:
Click here for full text options

Link to the External Link Resolver:
SFX Link

38.

Accuracy and Reliability of Multiphase CTA Perfusion for Identifying Ischemic Core.
Reid M., Famuyide A.O., Forkert N.D., Sahand Talai A., Evans J.W., Sitaram A., Hafeez M., Najm M., Menon B.K., Demchuk A., Goyal M., Gupta Sah R., d'Esterre C.D., Barber P.
Embase
Clinical Neuroradiology. 29 (3) (pp 543-552), 2019. Date of Publication: 01 Sep 2019.

[Article]
AN: 623717163
Publisher
Urban und Vogel GmbH

Link to the Ovid Full Text or citation:
Click here for full text options

Link to the External Link Resolver:
SFX Link

39.

The Prognostic Significance of Quantitative Myocardial Perfusion: An Artificial Intelligence-Based Approach Using Perfusion Mapping.
Knott K.D., Seraphim A., Augusto J.B., Xue H., Chacko L., Aung N., Petersen S.E., Cooper J.A., Manisty C., Bhuva A.N., Kotecha T., Bourantas C.V., Davies R.H., Brown L.A.E., Plein S., Fontana M., Kellman P., Moon J.C.
Embase
Circulation. (pp 1282-1291), 2020. Date of Publication: 2020.
[Article]

AN: 631606566
Publisher
Lippincott Williams and Wilkins (E-mail: kathiest.clai@apta.org)

Link to the Ovid Full Text or citation:
Click here for full text options

Link to the External Link Resolver:
SFX Link

40.

Global Developments in Stress Perfusion Cardiovascular Magnetic Resonance.
Arai A.E., Hsu L.-Y.
Embase
Circulation. (pp 1292-1294), 2020. Date of Publication: 2020.
[Review]
AN: 631605205
Publisher
Lippincott Williams and Wilkins (E-mail: kathiest.clai@apta.org)

Link to the Ovid Full Text or citation:
Click here for full text options

Link to the External Link Resolver:
SFX Link

41.

Machine Learning and Deep Neural Networks Applications in Computed Tomography for Coronary Artery Disease and Myocardial Perfusion.
Monti C.B., Codari M., van Assen M., De Cecco C.N., Vliegenthart R.
Embase
Journal of thoracic imaging. 35 (Supplement 1) (pp S58-S65), 2020. Date of Publication: 01 May 2020.
[Article]
AN: 631289670
Publisher
NLM (Medline)

Link to the Ovid Full Text or citation:
Click here for full text options

Link to the External Link Resolver:
SFX Link

42.

Rapid dealiasing of undersampled, non-Cartesian cardiac perfusion images using U-net.
Fan L., Shen D., Haji-Valizadeh H., Naresh N.K., Carr J.C., Freed B.H., Lee D.C., Kim D.
Embase
NMR in Biomedicine. 33 (5) (no pagination), 2020. Article Number: e4239. Date of Publication: 01 May 2020.
[Article]
AN: 2004064514
Publisher
John Wiley and Sons Ltd (Southern Gate, Chichester, West Sussex PO19 8SQ, United Kingdom. E-mail: vgorayska@wiley.com)

Link to the Ovid Full Text or citation:
Click here for full text options

Link to the External Link Resolver:
SFX Link

43.

Predicting the Need for Revascularization in Stable Coronary Artery Disease: Protons or Photons?.
Leiner T., Takx R.A.P.
Embase
JACC: Cardiovascular Imaging. 13 (4) (pp 1005-1007), 2020. Date of Publication: April 2020.
[Editorial]
AN: 2003313024
Publisher
Elsevier Inc. (E-mail: usjcs@elsevier.com)

Link to the Ovid Full Text or citation:
Click here for full text options

Link to the External Link Resolver:
SFX Link

44.

Intradialytic Hypotension: Mechanisms and Outcome.
Sars B., Van Der Sande F.M., Kooman J.P.
Embase
Blood Purification. 49 (1-2) (pp 158-167), 2020. Date of Publication: 01 Feb 2020.
[Review]
AN: 630297494
Publisher
S. Karger AG

Link to the Ovid Full Text or citation:
[Click here for full text options]

Link to the External Link Resolver:
[SFX Link]

45.

Future directions in coronary CT angiography: CT-fractional flow reserve, plaque vulnerability, and quantitative plaque assessment.
Kay F.U., Canan A., Abbara S.
Embase
Korean Circulation Journal. 50 (3) (pp 185-202), 2020. Date of Publication: 2020.
[Review]
AN: 2005208104
Publisher
Korean Society of Circulation (E-mail: herz4@circulation.or.kr)

Link to the Ovid Full Text or citation:
[Click here for full text options]

Link to the External Link Resolver:
[SFX Link]

46.

Evaluation of Lung Quality by Near-Infrared Fluorescent Imaging during Ex Vivo Lung Perfusion.
Nykanen A.I., Mariscal A., Ali A., Chen M., Gokhale H., Shan H., Cypel M., Liu M., Keshavjee S.
Embase
Journal of Heart and Lung Transplantation. Conference: 2020 Anniversary Meeting and Scientific Sessions - ISHLT. Canada. 39 (4 Supplement) (pp S179), 2020. Date of Publication: April 2020.
[Conference Abstract]
AN: 2005251197
Publisher
Elsevier USA

Link to the Ovid Full Text or citation:
[Click here for full text options]

Link to the External Link Resolver:
[SFX Link]

47.

Neural network-derived perfusion maps for the assessment of lesions in patients with acute ischemic stroke.
Meier R., Lux P., Jung S., Fischer U., Gralla J., Reyes M., Wiest R., McKinley R., Kaesmacher J.
Embase
Radiology: Artificial Intelligence. 1 (5) (no pagination), 2019. Article Number: e190019. Date of Publication: September 2019.
[Article]
AN: 2003399876
Publisher
Radiological Society of North America Inc. (820 Jorie Boulevard, Oak Brook IL 60523-2251, United States)

Link to the Ovid Full Text or citation:
Click here for full text options

Link to the External Link Resolver:
SFX Link

48.

Imaging the heart and the brain: From the amygdala to arterial inflammation.
Luscher T.F.
Embase
European Heart Journal. 41 (6) (pp 727-730), 2020. Date of Publication: 01 Feb 2020.
[Article]
AN: 631117899
Publisher
Oxford University Press

Link to the Ovid Full Text or citation:
Click here for full text options

Link to the External Link Resolver:
SFX Link

49.

Machine learning in the integration of simple variables for identifying patients with myocardial ischemia.
Juarez-Orozco L.E., Knol R.J.J., Sanchez-Catasus C.A., Martinez-Manzanera O., van der Zant F.M., Knuuti J.
Embase
Journal of Nuclear Cardiology. 27 (1) (pp 147-155), 2020. Date of Publication: 01 Feb 2020.
[Article]
AN: 2004302084

Publisher
Springer

Link to the Ovid Full Text or citation:
[Click here for full text options]

Link to the External Link Resolver:
[SFX Link]

50.

Machine Learning based SpO2 Computation Using Reflectance Pulse Oximetry.
Venkat S., Arsath P S M.T.P.S., Alex A., S P P., Balamugesh, D J C., Joseph J., Sivaprakasam M.
Embase
Conference proceedings : ... Annual International Conference of the IEEE Engineering in Medicine and Biology Society. IEEE Engineering in Medicine and Biology Society. Annual Conference. 2019 (pp 482-485), 2019. Date of Publication: 01 Jul 2019.
[Article]
AN: 630651216
Publisher
NLM (Medline)

Link to the Ovid Full Text or citation:
[Click here for full text options]

Link to the External Link Resolver:
[SFX Link]

51.

Left ventricular mechanical dyssynchrony for CAD diagnosis: Does it have incremental clinical values?.
Jiang Z., Zhou W.
Embase
Journal of Nuclear Cardiology. 27 (1) (pp 251-253), 2020. Date of Publication: 01 Feb 2020.
[Editorial]
AN: 623950331
Publisher
Springer

Link to the Ovid Full Text or citation:
[Click here for full text options]

Link to the External Link Resolver:
[SFX Link]

52.

The machine learning approach: Artificial intelligence is coming to support critical clinical thinking.
Nappi C., Cuocolo A.
Embase
Journal of Nuclear Cardiology. 27 (1) (pp 156-158), 2020. Date of Publication: 01 Feb 2020.
[Editorial]
AN: 622668472
Publisher
Springer

Link to the Ovid Full Text or citation:
Click here for full text options

Link to the External Link Resolver:
SFX Link

53.

Hierarchical Bayesian myocardial perfusion quantification.
Scannell C.M., Chiribiri A., Villa A.D.M., Breeuwer M., Lee J.
Embase
Medical Image Analysis. 60 (no pagination), 2020. Article Number: 101611. Date of Publication: February 2020.
[Article]
AN: 2003881032
Publisher
Elsevier B.V.

Link to the Ovid Full Text or citation:
Click here for full text options

Link to the External Link Resolver:
SFX Link

54.

Automated calcium scores collected during myocardial perfusion imaging improve identification of obstructive coronary artery disease.
Dekker M., Waissi F., Bank I.E.M., Lessmann N., Isgum I., Velthuis B.K., Scholtens A.M., Leenders G.E., Pasterkamp G., de Kleijn D.P.V., Timmers L., Mosterd A.
Embase
IJC Heart and Vasculature. 26 (no pagination), 2020. Article Number: 100434. Date of Publication: February 2020.
[Article]

AN: 2003846650
Publisher
Elsevier Ireland Ltd (P.O. Box 211, Amsterdam 1000 AE, Netherlands)

Link to the Ovid Full Text or citation:
Click here for full text options

Link to the External Link Resolver:
SFX Link

55.

Automatic myocardial segmentation in dynamic contrast enhanced perfusion MRI using Monte Carlo dropout in an encoder-decoder convolutional neural network.
Kim Y.-C., Kim K.R., Choe Y.H.
Embase
Computer Methods and Programs in Biomedicine. 185 (no pagination), 2020. Article Number: 105150. Date of Publication: March 2020.
[Article]
AN: 2003546427
Publisher
Elsevier Ireland Ltd

Link to the Ovid Full Text or citation:
Click here for full text options

Link to the External Link Resolver:
SFX Link

56.

Microsphere skimming in the porcine coronary arteries: Implications for flow quantification.
Sinclair M., Lee J., Schuster A., Chiribiri A., van den Wijngaard J., van Horssen P., Siebes M., Spaan J.A.E., Nagel E., Smith N.P.
Embase
Microvascular Research. 100 (pp 59-70), 2015. Date of Publication: 01 Jul 2015.
[Article]
AN: 607406329
Publisher
Academic Press Inc. (E-mail: apjcs@harcourt.com)

Link to the Ovid Full Text or citation:
Click here for full text options

Link to the External Link Resolver:
SFX Link

57.

Prediction of Significant Vasospasm in Aneurysmal Subarachnoid Hemorrhage Using Automated Data.
Roederer A., Holmes J.H., Smith M.J., Lee I., Park S.
Embase
Neurocritical Care. 21 (3) (pp 444-450), 2014. Date of Publication: 2014.
[Article]
AN: 53096035
Publisher
Humana Press Inc. (E-mail: humana@humanapr.com)

Link to the Ovid Full Text or citation:
Click here for full text options

Link to the External Link Resolver:
SFX Link

58.

Quantitative cardiac MRI.
Seraphim A., Knott K.D., Augusto J., Bhuva A.N., Manisty C., Moon J.C.
Embase
Journal of Magnetic Resonance Imaging. 51 (3) (pp 693-711), 2020. Date of Publication: 01 Mar 2020.
[Review]
AN: 627846427
Publisher
John Wiley and Sons Inc. (P.O.Box 18667, Newark NJ 07191-8667, United States)

Link to the Ovid Full Text or citation:
Click here for full text options

Link to the External Link Resolver:
SFX Link

59.

Imaging Methods for Ultrasound Contrast Agents.
Averkiou M.A., Bruce M.F., Powers J.E., Sheeran P.S., Burns P.N.
Embase
Ultrasound in Medicine and Biology. 46 (3) (pp 498-517), 2020. Date of Publication: March 2020.
[Review]
AN: 2004120958
Publisher
Elsevier USA

Link to the Ovid Full Text or citation:
Click here for full text options

Link to the External Link Resolver:
SFX Link

60.

Wearable Sensors Reveal Menses-Driven Changes in Physiology and Enable Prediction of the Fertile Window: Observational Study.
Goodale B.M., Shilaih M., Falco L., Dammeier F., Hamvas G., Leeners B.
Embase
Journal of medical Internet research. 21 (4) (pp e13404), 2019. Date of Publication: 18 Apr 2019.
[Article]
AN: 627477057
Publisher
NLM (Medline)

Link to the Ovid Full Text or citation:
Click here for full text options

Link to the External Link Resolver:
SFX Link

61.

Decreased skeletal muscle, but not increased fat is independently associated with coronary microvascular dysfunction and adverse cardiovascular outcomes.
Souza A.C.D., Rosenthal M., Divakaran S., Osborne M.T., Bajaj N.S., Bibbo C.F., Hainer J., Blankstein R., Dorbala S., Di Carli M.F., Taquet V.R.
Embase
Circulation. Conference: American Heart Association Scientific Sessions, AHA 2019. United States. 140 (Supplement 1) (no pagination), 2019. Date of Publication: 2019.
[Conference Abstract]
AN: 630921245
Publisher
Lippincott Williams and Wilkins

Link to the Ovid Full Text or citation:
Click here for full text options

Link to the External Link Resolver:
SFX Link

62.

Application of data mining in a cohort of Italian subjects undergoing myocardial perfusion imaging at an academic medical center.
Ricciardi C., Cantoni V., Improta G., Iuppariello L., Latessa I., Cesarelli M., Triassi M., Cuocolo A.
Embase
Computer Methods and Programs in Biomedicine. 189 (no pagination), 2020. Article Number: 105343. Date of Publication: June 2020.
[Article]
AN: 2004681112
Publisher
Elsevier Ireland Ltd

Link to the Ovid Full Text or citation:
[Click here for full text options]

Link to the External Link Resolver:
[SFX Link]

63.

Accuracy, uncertainty, and adaptability of automatic myocardial ASL segmentation using deep CNN.
Do H.P., Guo Y., Yoon A.J., Nayak K.S.
Embase
Magnetic Resonance in Medicine. 83 (5) (pp 1863-1874), 2020. Date of Publication: 01 May 2020.
[Article]
AN: 2003639425
Publisher
John Wiley and Sons Inc. (P.O.Box 18667, Newark NJ 07191-8667, United States)

Link to the Ovid Full Text or citation:
[Click here for full text options]

Link to the External Link Resolver:
[SFX Link]

64.

Quantitative perfusion mapping in Fabry disease.
Knott K., Augusto J.B., Nordin S., Kozor R., Camaioni C., Xue H., Hughes R.K., Manisty C., Brown L.A.E., Ramaswami U., Hughes D., Kellman P., Plein S., Moon J.C.
Embase
European Heart Journal Cardiovascular Imaging. Conference: 16th International Congress on Cardiovascular Magnetic Resonance, EuroCMR 2019. Italy. 20 (Supplement 2) (pp ii224-ii225), 2019. Date of Publication: June 2019.
[Conference Abstract]

AN: 630690585
Publisher
Oxford University Press

Link to the Ovid Full Text or citation:
Click here for full text options

Link to the External Link Resolver:
SFX Link

65.

Predicting Hemodynamic Shock from Thermal Images using Machine Learning.
Nagori A., Dhingra L.S., Bhatnagar A., Lodha R., Sethi T.
Embase
Scientific reports. 9 (1) (pp 91), 2019. Date of Publication: 14 Jan 2019.
[Article]
AN: 625943668
Publisher
NLM (Medline)

Link to the Ovid Full Text or citation:
Click here for full text options

Link to the External Link Resolver:
SFX Link

66.

SLICR super-voxel algorithm for fast, robust quantification of myocardial blood flow by dynamic computed tomography myocardial perfusion imaging.
Wu H., Eck B.L., Levi J., Fares A., Li Y., Wen D., Bezerra H.G., Muzic R.F., Wilson D.L.
Embase
Journal of Medical Imaging. 6 (4) (no pagination), 2019. Article Number: 046001. Date of Publication: 01 Oct 2019.
[Article]
AN: 630451551
Publisher
SPIE (E-mail: spie@spie.org)

Link to the Ovid Full Text or citation:
Click here for full text options

Link to the External Link Resolver:
SFX Link

67.

Canadian Cardiovascular Congress 2019.
Anonymous
Embase
Canadian Journal of Cardiology. Conference: Canadian Cardiovascular Congress 2019. Canada. 35 (10 Supplement) (pp A1-A18), 2019. Date of Publication: October 2019.
[Conference Review]
AN: 2003291445
Publisher
Elsevier Inc.

Link to the Ovid Full Text or citation:
Click here for full text options

Link to the External Link Resolver:
SFX Link

68.

Nursing research on patients with hypertensive intracerebral hemorrhage based on fuzzy neural network related algorithm under postural adjustment.
Qu D., Ding C.
Embase
Journal of Medical Imaging and Health Informatics. 10 (2) (pp 422-427), 2020. Date of Publication: 2020.
[Article]
AN: 2004327897
Publisher
American Scientific Publishers (E-mail: order@aspbs.com)

Link to the Ovid Full Text or citation:
Click here for full text options

Link to the External Link Resolver:
SFX Link

69.

Deep Learning in Quantitative PET Myocardial Perfusion Imaging: A Study on Cardiovascular Event Prediction.
Juarez-Orozco L.E., Martinez-Manzanera O., van der Zant F.M., Knol R.J.J., Knuuti J.
Embase
JACC: Cardiovascular Imaging. Part 1. 13 (1) (pp 180-182), 2020. Date of Publication: January 2020.
[Letter]
AN: 2003313041

Publisher
Elsevier Inc. (E-mail: usjcs@elsevier.com)

Link to the Ovid Full Text or citation:
[Click here for full text options]

Link to the External Link Resolver:
[SFX Link]

70.

CT based arterial and venous small vessel volume and right ventricular dysfunction in acute submassive pulmonary embolism.
Rahaghi F.N., Nardelli P., Minhas J., Hassan S., Ash S., Gonzalez G., Ross J., Hunsaker A., Piazza G., Washko G.R., San Jose Estepar R.
Embase
American Journal of Respiratory and Critical Care Medicine. Conference: 2019 International Conference of the American Thoracic Society , ATS 2019. United States. 199 (9) (no pagination), 2019. Date of Publication: May 2019.
[Conference Abstract]
AN: 630352741
Publisher
American Thoracic Society

Link to the Ovid Full Text or citation:
[Click here for full text options]

Link to the External Link Resolver:
[SFX Link]

71.

Optimizing conduit vessel removal from perfusion quantification using arterial spin labeling magnetic response imaging via in silico modeling.
Addo D.A., Elliot A.R., Thielmann R., Niese A., Darquenne C., Prisk G.K., Tawhai M.H., Burrowes K.S.
Embase
American Journal of Respiratory and Critical Care Medicine. Conference: 2019 International Conference of the American Thoracic Society , ATS 2019. United States. 199 (9) (no pagination), 2019. Date of Publication: May 2019.
[Conference Abstract]
AN: 630347507
Publisher
American Thoracic Society

Link to the Ovid Full Text or citation:
[Click here for full text options]

Link to the External Link Resolver:

SFX Link

72.

Intelligent Imaging: Radiomics and Artificial Neural Networks in Heart Failure.
Currie G., Iqbal B., Kiat H.
Embase
Journal of Medical Imaging and Radiation Sciences. 50 (4) (pp 571-574), 2019. Date of Publication: December 2019.
[Article]
AN: 2003180995
Publisher
Elsevier Inc. (E-mail: usjcs@elsevier.com)

Link to the Ovid Full Text or citation:
Click here for full text options

Link to the External Link Resolver:
SFX Link

73.

Long-term prognostic value of quantitative myocardial perfusion in patients with chest pain and normal coronary arteries.
Monroy-Gonzalez A.G., Tio R.A., de Groot J.C., Boersma H.H., Prakken N.H., De Jongste M.J.L., Alexanderson-Rosas E., Slart R.H.J.A.
Embase
Journal of Nuclear Cardiology. 26 (6) (pp 1844-1852), 2019. Date of Publication: 01 Dec 2019.
[Article]
AN: 624271999
Publisher
Springer

Link to the Ovid Full Text or citation:
Click here for full text options

Link to the External Link Resolver:
SFX Link

74.

Progress in Cardiovascular Imaging.
Shaw L., Chandrashekhar Y.
Embase
JACC: Cardiovascular Imaging. 12 (12) (pp 2589-2610), 2019. Date of Publication: December 2019.

[Review]
AN: 2003911142
Publisher
Elsevier Inc. (E-mail: usjcs@elsevier.com)

Link to the Ovid Full Text or citation:
Click here for full text options

Link to the External Link Resolver:
SFX Link

75.

PET [11C]acetate is also a perfusion tracer for kidney evaluation purposes.
Normand G., Lemoine S., Le Bars D., Merida I., Irace Z., Troalen T., Costes N., Juillard L.
Embase
Nuclear Medicine and Biology. 76-77 (pp 10-14), 2019. Date of Publication: September - October 2019.
[Article]
AN: 2003556176
Publisher
Elsevier Inc. (E-mail: usjcs@elsevier.com)

Link to the Ovid Full Text or citation:
Click here for full text options

Link to the External Link Resolver:
SFX Link

76.

Quantitative myocardial perfusion in coronary artery disease: A perfusion mapping study.
Knott K.D., Camaioni C., Ramasamy A., Augusto J.A., Bhuva A.N., Xue H., Manisty C., Hughes R.K., Brown L.A.E., Amersey R., Bourantas C., Kellman P., Plein S., Moon J.C.
Embase
Journal of Magnetic Resonance Imaging. 50 (3) (pp 756-762), 2019. Date of Publication: 2019.
[Article]
AN: 626129340
Publisher
John Wiley and Sons Inc. (P.O.Box 18667, Newark NJ 07191-8667, United States)

Link to the Ovid Full Text or citation:
Click here for full text options

Link to the External Link Resolver:

SFX Link

77.

Unmasking the Hypovolemic Shock Continuum: The Compensatory Reserve.
Suresh M.R., Chung K.K., Schiller A.M., Holley A.B., Howard J.T., Convertino V.A.
Embase
Journal of Intensive Care Medicine. 34 (9) (pp 696-706), 2019. Date of Publication: 01 Sep 2019.
[Review]
AN: 623636519
Publisher
SAGE Publications Inc. (E-mail: claims@sagepub.com)

Link to the Ovid Full Text or citation:
Click here for full text options

Link to the External Link Resolver:
SFX Link

78.

The Current Role of Viability Imaging to Guide Revascularization and Therapy Decisions in Patients With Heart Failure and Reduced Left Ventricular Function.
Kandolin R.M., Wiefels C.C., Mesquita C.T., Chong A.-Y., Boland P., Glineur D., Sun L., Beanlands R.S., Mielniczuk L.M.
Embase
Canadian Journal of Cardiology. 35 (8) (pp 1015-1029), 2019. Date of Publication: August 2019.
[Review]
AN: 2002440910
Publisher
Elsevier Inc. (E-mail: usjcs@elsevier.com)

Link to the Ovid Full Text or citation:
Click here for full text options

Link to the External Link Resolver:
SFX Link

79.

Automatic myocardial ischemic lesion detection on magnetic resonance perfusion weighted imaging prior perfusion quantification: A pre-modeling strategy.
Daviller C., Grenier T., Ratiney H., Sdika M., Croisille P., Viallon M.
Embase
Computers in Biology and Medicine. 110 (pp 108-119), 2019. Date of Publication:

July 2019.
[Article]
AN: 2002034711
Publisher
Elsevier Ltd

Link to the Ovid Full Text or citation:
Click here for full text options

Link to the External Link Resolver:
SFX Link

80.

Future of Echocardiography in Australia.
Marwick T.H.
Embase
Heart Lung and Circulation. 28 (9) (pp 1307-1309), 2019. Date of Publication: September 2019.
[Editorial]
AN: 2001597791
Publisher
Elsevier Ltd

Link to the Ovid Full Text or citation:
Click here for full text options

Link to the External Link Resolver:
SFX Link

81.

Prognostic value of CT myocardial perfusion imaging and CT-derived fractional flow reserve for major adverse cardiac events in patients with coronary artery disease.
van Assen M., De Cecco C.N., Eid M., von Knebel Doeberitz P., Scarabello M., Lavra F., Bauer M.J., Mastrodicasa D., Duguay T.M., Zaki B., Lo G.G., Choe Y.H., Wang Y., Sahbaee P., Tesche C., Oudkerk M., Vliegenthart R., Schoepf U.J.
Embase
Journal of Cardiovascular Computed Tomography. 13 (3) (pp 26-33), 2019. Date of Publication: May - June 2019.
[Article]
AN: 2001590739
Publisher
Elsevier Inc. (E-mail: usjcs@elsevier.com)

Link to the Ovid Full Text or citation:
Click here for full text options

Link to the External Link Resolver:

SFX Link

82.

Impact of non-specific normal databases on perfusion quantification of low-dose myocardial SPECT studies.
Scabbio C., Zoccarato O., Malaspina S., Lucignani G., Del Sole A., Lecchi M.
Embase
Journal of Nuclear Cardiology. 26 (3) (pp 775-785), 2019. Date of Publication: 15 Jun 2019.
[Article]
AN: 618797132
Publisher
Springer New York LLC (E-mail: barbara.b.bertram@gsk.com)

Link to the Ovid Full Text or citation:
Click here for full text options

Link to the External Link Resolver:
SFX Link

83.

EVCMR: A tool for the quantitative evaluation and visualization of cardiac MRI data.
Kim Y.-C., Kim K.R., Choi K., Kim M., Chung Y., Choe Y.H.
Embase
Computers in Biology and Medicine. 111 (no pagination), 2019. Article Number: 103334. Date of Publication: August 2019.
[Article]
AN: 2002223893
Publisher
Elsevier Ltd

Link to the Ovid Full Text or citation:
Click here for full text options

Link to the External Link Resolver:
SFX Link

84.

The Future of Cardiovascular Computed Tomography: Advanced Analytics and Clinical Insights.
Nicol E.D., Norgaard B.L., Blanke P., Ahmadi A., Weir-McCall J., Horvat P.M., Han K., Bax J.J., Leipsic J.
Embase
JACC: Cardiovascular Imaging. 12 (6) (pp 1058-1072), 2019. Date of Publication:

June 2019.
[Review]
AN: 2002013544
Publisher
Elsevier Inc. (E-mail: usjcs@elsevier.com)

Link to the Ovid Full Text or citation:
Click here for full text options

Link to the External Link Resolver:
SFX Link

85.

Nuclear cardiology in the literature: A selection of recent, original research papers.
Malhotra S.
Embase
Journal of Nuclear Cardiology. 26 (2) (pp 363-365), 2019. Date of Publication: 15 Apr 2019.
[Review]
AN: 626254997
Publisher
Springer New York LLC (E-mail: barbara.b.bertram@gsk.com)

Link to the Ovid Full Text or citation:
Click here for full text options

Link to the External Link Resolver:
SFX Link

86.

Quantitative myocardial perfusion in Fabry disease.
Knott K.D., Augusto J.B., Nordin S., Kozor R., Camaioni C., Xue H., Hughes R.K., Manisty C., Brown L.A.E., Kellman P., Ramaswami U., Hughes D., Plein S., Moon J.C.
Embase
Circulation: Cardiovascular Imaging. 12 (7) (no pagination), 2019. Article Number: e008872. Date of Publication: 01 Jul 2019.
[Editorial]
AN: 629638892
Publisher
Lippincott Williams and Wilkins (E-mail: kathiest.clai@apta.org)

Link to the Ovid Full Text or citation:
Click here for full text options

Link to the External Link Resolver:
SFX Link

87.

Machine learning for nuclear cardiology: The way forward.
Shrestha S., Sengupta P.P.
Embase
Journal of Nuclear Cardiology. 26 (5) (pp 1755-1758), 2019. Date of Publication: 01 Oct 2019.
[Editorial]
AN: 621778629
Publisher
Springer New York LLC (E-mail: barbara.b.bertram@gsk.com)

Link to the Ovid Full Text or citation:
Click here for full text options

Link to the External Link Resolver:
SFX Link

88.

Prediction of cardiac death after adenosine myocardial perfusion SPECT based on machine learning.
Haro Alonso D., Wernick M.N., Yang Y., Germano G., Berman D.S., Slomka P.
Embase
Journal of Nuclear Cardiology. 26 (5) (pp 1746-1754), 2019. Date of Publication: 01 Oct 2019.
[Article]
AN: 621252175
Publisher
Springer New York LLC (E-mail: barbara.b.bertram@gsk.com)

Link to the Ovid Full Text or citation:
Click here for full text options

Link to the External Link Resolver:
SFX Link

89.

FFR-CT and CT Myocardial Perfusion Imaging: Friends or Foes?.
Schoepf U.J., van Assen M.
Embase
JACC: Cardiovascular Imaging. 12 (12) (pp 2472-2474), 2019. Date of Publication: December 2019.
[Editorial]
AN: 2003911125

Publisher
Elsevier Inc. (E-mail: usjcs@elsevier.com)

Link to the Ovid Full Text or citation:
Click here for full text options

Link to the External Link Resolver:
SFX Link

90.

Hemodynamically significant coronary stenosis: Detection with CT Myocardial Perfusion Imaging versus Machine Learning Coronary CT Fractional Flow Reserve.
Loewe C.
Embase
Radiology. 293 (2) (pp 315-316), 2019. Date of Publication: 2019.
[Editorial]
AN: 2003834658
Publisher
Radiological Society of North America Inc. (820 Jorie Boulevard, Oak Brook IL 60523-2251, United States)

Link to the Ovid Full Text or citation:
Click here for full text options

Link to the External Link Resolver:
SFX Link

91.

Detection of hemodynamically significant coronary stenosis: CT Myocardial Perfusion versus Machine Learning CT Fractional Flow Reserve.
Li Y., Yu M., Dai X., Lu Z., Shen C., Wang Y., Lu B., Zhang J.
Embase
Radiology. 293 (2) (pp 305-314), 2019. Date of Publication: 2019.
[Article]
AN: 2003834657
Publisher
Radiological Society of North America Inc. (820 Jorie Boulevard, Oak Brook IL 60523-2251, United States)

Link to the Ovid Full Text or citation:
Click here for full text options

Link to the External Link Resolver:
SFX Link

92.

Advances in MRI Applications to Diagnose and Manage Cardiomyopathies.
Vajapey R., Eck B., Tang W., Kwon D.H.
Embase
Current Treatment Options in Cardiovascular Medicine. 21 (11) (no pagination), 2019. Article Number: 74. Date of Publication: 01 Nov 2019.
[Review]
AN: 2003752514
Publisher
Springer

Link to the Ovid Full Text or citation:
Click here for full text options

Link to the External Link Resolver:
SFX Link

93.

Leveraging latest computer science tools to advance nuclear cardiology.
Slomka P.
Embase
Journal of Nuclear Cardiology. 26 (5) (pp 1501-1504), 2019. Date of Publication: 01 Oct 2019.
[Editorial]
AN: 2003489264
Publisher
Springer New York LLC

Link to the Ovid Full Text or citation:
Click here for full text options

Link to the External Link Resolver:
SFX Link

94.

Is there value for artificial intelligence applications in molecular imaging and nuclear medicine?.
Porenta G.
Embase
Journal of Nuclear Medicine. 60 (10) (pp 1347-1349), 2019. Date of Publication: 01 Oct 2019.
[Short Survey]
AN: 2003127936
Publisher
Society of Nuclear Medicine Inc. (E-mail: subscriptions@snmmi.org)

Link to the Ovid Full Text or citation:
Click here for full text options

Link to the External Link Resolver:
SFX Link

95.

Statement on imaging and pulmonary hypertension from the Pulmonary Vascular Research Institute (PVRI).
Kiely D.G., Levin D.L., Hassoun P.M., Ivy D., Jone P.-N., Bwika J., Kawut S.M., Lordan J., Lungu A., Mazurek J.A., Moledina S., Olschewski H., Peacock A.J., Puri G.D., Rahaghi F.N., Schafer M., Schiebler M., Screaton N., Tawhai M., van Beek E.J.R., Vonk-Noordegraaf A., Vandepool R., Wort S.J., Zhao L., Wild J.M., Vogel-Claussen J., Swift A.J.
Embase
Pulmonary Circulation. 9 (3) (no pagination), 2019. Date of Publication: 2019.
[Article]
AN: 2002865281
Publisher
SAGE Publications Ltd (E-mail: info@sagepub.co.uk)

Link to the Ovid Full Text or citation:
Click here for full text options

Link to the External Link Resolver:
SFX Link

96.

Neuroimaging and Machine Learning for Dementia Diagnosis: Recent Advancements and Future Prospects.
Ahmed M.R., Zhang Y., Feng Z., Lo B., Inan O.T., Liao H.
Embase
IEEE Reviews in Biomedical Engineering. 12 (pp 19-33), 2019. Article Number: 8572804. Date of Publication: 2019.
[Review]
AN: 625526511
Publisher
Institute of Electrical and Electronics Engineers

Link to the Ovid Full Text or citation:
Click here for full text options

Link to the External Link Resolver:
SFX Link

97.

Improved diagnostic accuracy for myocardial perfusion imaging using artificial neural networks on different input variables including clinical and quantification data.
Precision diagnostica mejorada para la imagen de perfusion miocardica usando redes neuronales artificiales en diferentes variables de entrada incluyendo datos clinicos y de cuantificacion <Precision diagnostica mejorada para la imagen de perfusion miocardica usando redes neuronales artificiales en diferentes variables de entrada incluyendo datos clinicos y de cuantificacion.>
Rahmani R., Niazi P., Naseri M., Neishabouri M., Farzanefar S., Eftekhari M., Derakhshan F., Mollazadeh R., Meysami A., Abbasi M.
Embase
Revista Espanola de Medicina Nuclear e Imagen Molecular. 38 (5) (pp 275-279), 2019. Date of Publication: September - October 2019.
[Article]
AN: 2002511334
Publisher
Ediciones Doyma, S.L.

Link to the Ovid Full Text or citation:
Click here for full text options

Link to the External Link Resolver:
SFX Link

98.

Artificial intelligence in nuclear cardiology.
Gomez J., Doukky R.
Embase
Journal of Nuclear Medicine. 60 (8) (pp 1042-1043), 2019. Date of Publication: 01 Aug 2019.
[Short Survey]
AN: 2002452830
Publisher
Society of Nuclear Medicine Inc. (E-mail: subscriptions@snmmi.org)

Link to the Ovid Full Text or citation:
Click here for full text options

Link to the External Link Resolver:
SFX Link

99.

Simulation of the perfusion of contrast agent used in cardiac magnetic resonance: A Step Toward Non-invasive Cardiac Perfusion Quantification.
Alves J.R., De Queiroz R.A.B., Bar M., Dos Santos R.W.
Embase

Frontiers in Physiology. 10 (MAR) (no pagination), 2019. Article Number: 177. Date of Publication: 2019.
[Article]
AN: 627944777
Publisher
Frontiers Media S.A. (E-mail: info@frontiersin.org)

Link to the Ovid Full Text or citation:
Click here for full text options

Link to the External Link Resolver:
SFX Link

100.

Accuracy of an artificial neural network for detecting a regional abnormality in myocardial perfusion SPECT.
Shibutani T., Nakajima K., Wakabayashi H., Mori H., Matsuo S., Yoneyama H., Konishi T., Okuda K., Onoguchi M., Kinuya S.
Embase
Annals of Nuclear Medicine. 33 (2) (pp 86-92), 2019. Date of Publication: 25 Feb 2019.
[Article]
AN: 624434220
Publisher
Springer Tokyo (E-mail: orders@springer.jp)

Link to the Ovid Full Text or citation:
Click here for full text options

Link to the External Link Resolver:
SFX Link

101.

Deep learning analysis of upright-supine high-efficiency SPECT myocardial perfusion imaging for prediction of obstructive coronary artery disease: A multicenter study.
Betancur J., Hu L.-H., Commandeur F., Sharir T., Einstein A.J., Fish M.B., Ruddy T.D., Kaufmann P.A., Sinusas A.J., Miller E.J., Bateman T.M., Dorbala S., Carli M.D., Germano G., Otaki Y., Liang J.X., Tamarappoo B.K., Dey D., Berman D.S., Slomka P.J.
Embase
Journal of Nuclear Medicine. 60 (5) (pp 664-670), 2019. Date of Publication: 01 May 2019.
[Article]
AN: 2002061114
Publisher
Society of Nuclear Medicine Inc. (E-mail: MemberInfo@snm.org)

Link to the Ovid Full Text or citation:
Click here for full text options

Link to the External Link Resolver:
SFX Link

102.

Artificial Intelligence in Nuclear Cardiology: Adding Value to Prognostication.
Seetharam K., Shresthra S., Mills J.D., Sengupta P.P.
Embase
Current Cardiovascular Imaging Reports. 12 (5) (no pagination), 2019. Article Number: 14. Date of Publication: 01 May 2019.
[Review]
AN: 626826458
Publisher
Current Medicine Group LLC 1 (E-mail: info@phl.cursci.com)

Link to the Ovid Full Text or citation:
Click here for full text options

Link to the External Link Resolver:
SFX Link

103.

Application of speCtraL computed tomogrAphy to impRove specIficity of cardiac compuTed tomographY (CLARITY study): Rationale and design.
Van Hamersvelt R.W., Isgum I., De Jong P.A., Cramer M.J.M., Leenders G.E.H., Willemink M.J., Voskuil M., Leiner T.
Embase
BMJ Open. 9 (3) (no pagination), 2019. Article Number: e025793. Date of Publication: 01 Mar 2019.
[Article]
AN: 626568370
Publisher
BMJ Publishing Group (E-mail: subscriptions@bmjgroup.com)

Link to the Ovid Full Text or citation:
Click here for full text options

Link to the External Link Resolver:
SFX Link

104.

Impact of low-dose SPECT imaging on normal databases and myocardial perfusion

scores.
Scabbio C., Malaspina S., Capozza A., Selvaggi C., Matheoud R., Del Sole A., Lecchi M.
Embase
Physica Medica. 59 (pp 163-169), 2019. Date of Publication: March 2019.
[Article]
AN: 2001698247
Publisher
Associazione Italiana di Fisica Medica

Link to the Ovid Full Text or citation:
Click here for full text options

Link to the External Link Resolver:
SFX Link

105.

Machine Learning in the Evaluation of Myocardial Ischemia Through Nuclear Cardiology.
Juarez-Orozco L.E., Martinez-Manzanera O., Storti A.E., Knuuti J.
Embase
Current Cardiovascular Imaging Reports. 12 (2) (no pagination), 2019. Article Number: 5. Date of Publication: 01 Feb 2019.
[Review]
AN: 626285863
Publisher
Current Medicine Group LLC 1 (E-mail: info@phl.cursci.com)

Link to the Ovid Full Text or citation:
Click here for full text options

Link to the External Link Resolver:
SFX Link

106.

Automatic in-line quantitative myocardial perfusion mapping: Processing algorithm and implementation.
Xue H., Brown L.A.E., Nielles-Vallespin S., Plein S., Kellman P.
Embase
Magnetic Resonance in Medicine. (no pagination), 2019. Date of Publication: 2019.
[Article]
AN: 2002609102
Publisher
John Wiley and Sons Inc. (P.O.Box 18667, Newark NJ 07191-8667, United States)

Link to the Ovid Full Text or citation:
Click here for full text options

Link to the External Link Resolver:
SFX Link

107.

Head-to-head comparison of diagnostic accuracy of stress-only myocardial perfusion imaging with conventional and cadmium-zinc telluride single-photon emission computed tomography in women with suspected coronary artery disease.
Mannarino T., Assante R., Ricciardi C., Zampella E., Nappi C., Gaudieri V., Mainolfi C.G., Di Vaia E., Petretta M., Cesarelli M., Cuocolo A., Acampa W.
Embase
Journal of Nuclear Cardiology. (no pagination), 2019. Date of Publication: 2019.
[Article]
AN: 628321941
Publisher
Springer New York LLC (E-mail: barbara.b.bertram@gsk.com)

Link to the Ovid Full Text or citation:
Click here for full text options

Link to the External Link Resolver:
SFX Link

108.

Personalized Models for Injected Activity Levels in SPECT Myocardial Perfusion Imaging.
Juan Ramon A., Yang Y., Pretorius P.H., Johnson K.L., King M.A., Wernick M.N.
Embase
IEEE Transactions on Medical Imaging. 38 (6) (pp 1466-1476), 2019. Article Number: 8565970. Date of Publication: June 2019.
[Article]
AN: 625394482
Publisher
Institute of Electrical and Electronics Engineers Inc.

Link to the Ovid Full Text or citation:
Click here for full text options

Link to the External Link Resolver:
SFX Link

109.

PREDICTING MAJOR ADVERSE CARDIAC EVENTS WITH COX NEURAL NETWORKS: RESULTS FROM THE REFINE SPECT REGISTRY.

Slomka P., Betancur J., Otaki Y., Commandeur F., Sharir T., Einstein A., Fish M., Ruddy T., Kaufmann P.A., Sinusas A., Miller E., Bateman T., Dorbala S., Di Carli M., Diniz M., Germano G., Dey D., Cooper L., Berman D.
Embase
Journal of the American College of Cardiology. Conference: 68th Annual Scientific Session of the American College of Cardiology: ACC.19. United States. 73 (9 Supplement 1) (pp 1432), 2019. Date of Publication: 12 March 2019.
[Conference Abstract]
AN: 2001641989
Publisher
Elsevier USA

Link to the Ovid Full Text or citation:
Click here for full text options

Link to the External Link Resolver:
SFX Link

110.

UTILIZING RUBIDIUM-82 CARDIAC POSITRON EMISSION TOMOGRAPHY REGIONAL MYOCARDIAL BLOOD FLOW TO DEVELOP NAIVE BAYES CLASSIFIER FOR CORONARY ARTERY DISEASE PREDICTION.
Rao H.S., Guerraty M., Dubroff J., Rader D.
Embase
Journal of the American College of Cardiology. Conference: 68th Annual Scientific Session of the American College of Cardiology: ACC.19. United States. 73 (9 Supplement 1) (pp 1484), 2019. Date of Publication: 12 March 2019.
[Conference Abstract]
AN: 2001640360
Publisher
Elsevier USA

Link to the Ovid Full Text or citation:
Click here for full text options

Link to the External Link Resolver:
SFX Link

111.

Corrigendum to "Automatic myocardial ischemic lesion detection on magnetic resonance perfusion weighted imaging prior perfusion quantification: A pre-modeling strategy" [Comput. Biol. Med. 110 (2019) 108-119](S0010482519301519)(10.1016/j.compbiomed.2019.05.001).
Daviller C., Grenier T., Ratiney H., Sdika M., Croisille P., Viallon M.
Embase
Computers in Biology and Medicine. 114 (no pagination), 2019. Article Number: 103455. Date of Publication: November 2019.
[Erratum]

AN: 2003136227
Publisher
Elsevier Ltd

Link to the Ovid Full Text or citation:
[Click here for full text options]

Link to the External Link Resolver:
[SFX Link]

112.

Novel Metric for Evaluating Ischemic Stress in Electrograms Using a Data-Driven Approach.
Good W.W., Erem B., Coll-Font J., Brooks D.H., MacLeod R.S.
Embase
Journal of Electrocardiology. Conference: ISCE 35th Annual Meeting. United States. 57 (Supplement) (pp S112-S113), 2019. Date of Publication: November - December 2019.
[Conference Abstract]
AN: 2003794177
Publisher
Churchill Livingstone Inc.

Link to the Ovid Full Text or citation:
[Click here for full text options]

Link to the External Link Resolver:
[SFX Link]

113.

ESC Congress 2019 together with World Congress of Cardiology.
Anonymous
Embase
European Heart Journal. Conference: European Society of Cardiology Congress, ESC 2019. France. 40 (Supplement 1) (no pagination), 2019. Date of Publication: October 2019.
[Conference Review]
AN: 630052086
Publisher
Oxford University Press

Link to the Ovid Full Text or citation:
[Click here for full text options]

Link to the External Link Resolver:
[SFX Link]

114.

Cost-saving diagnosis approach by artificial intelligence tool in patients with suspected coronary artery disease. the co-operative ARTICA registry database.
Mazzanti M., Shirka E., Gjergo H., Pugliese F., Goda A.
Embase
European Heart Journal. Conference: European Society of Cardiology Congress, ESC 2019. France. 40 (Supplement 1) (pp 3145), 2019. Date of Publication: October 2019.
[Conference Abstract]
AN: 630052329
Publisher
Oxford University Press

Link to the Ovid Full Text or citation:
Click here for full text options

Link to the External Link Resolver:
SFX Link

115.

Lifetime cost-effectiveness of diagnostic artificial intelligence tool for evaluating individuals with stable chest pain. The co-operative ARTICA registry database.
Mazzanti M., Shirka E., Gjergo H., Pugliese F., Goda A.
Embase
European Heart Journal. Conference: European Society of Cardiology Congress, ESC 2019. France. 40 (Supplement 1) (pp 439), 2019. Date of Publication: October 2019.
[Conference Abstract]
AN: 630050674
Publisher
Oxford University Press

Link to the Ovid Full Text or citation:
Click here for full text options

Link to the External Link Resolver:
SFX Link

116.

Deep learning survival analysis enhances the value of hybrid PET/CT for long-term cardiovascular event prediction.
Juarez-Orozco L.E., Benjamins J.W., Maaniitty T., Saraste A., Van Der Harst P., Knuuti J.
Embase

European Heart Journal. Conference: European Society of Cardiology Congress, ESC 2019. France. 40 (Supplement 1) (pp 675), 2019. Date of Publication: October 2019.
[Conference Abstract]
AN: 630050573
Publisher
Oxford University Press

Link to the Ovid Full Text or citation:
Click here for full text options

Link to the External Link Resolver:
SFX Link

117.

Prognostic safety of automatic cancellation of rest myocardial perfusion scan by machine learning: A report from multicenter REFINE SPECT registry of new generation SPECT.
Hu L., Sharir T., Fish M.B., Ruddy T.D., Di Carli M., Dorbala S., Einstein A.J., Betancur J., Eisenberg E., Commandeur F., Germano G., Damini D., Berman D., Slomka P.J.
Embase
European Heart Journal. Conference: European Society of Cardiology Congress, ESC 2019. France. 40 (Supplement 1) (pp 3), 2019. Date of Publication: October 2019.
[Conference Abstract]
AN: 630046216
Publisher
Oxford University Press

Link to the Ovid Full Text or citation:
Click here for full text options

Link to the External Link Resolver:
SFX Link

118.

Machine learning approach for prdiction of postinfarction myocardial recovery using echocardiographic myocardial texture.
Michalski B.W., Skonieczka S., Strzelecki M., Simiera M., Szymczyk E., Wejner-Mik P., Lipiec P., Wierzbowska-Drabik K., Kasprzak J.D.
Embase
European Heart Journal. Conference: European Society of Cardiology Congress, ESC 2019. France. 40 (Supplement 1) (pp 2616), 2019. Date of Publication: October 2019.
[Conference Abstract]
AN: 630049512
Publisher

Oxford University Press

Link to the Ovid Full Text or citation:
[Click here for full text options]

Link to the External Link Resolver:
[SFX Link]

119.

Artificial Intelligence Tools for Postinfarction Myocardial Recovery Prediction Based on Analysis of Echocardiographic Myocardial Texture.
Michalski B.W., Skonieczka S., Strzelecki M., Simiera M., Szymczyk E., Wejner-Mik P., Lipiec P., Wierzbowska-Drabik K., Kasprzak J.
Embase
Journal of the American Society of Echocardiography. Conference: 34th IEEE/ACM International Conference on Automated Software Engineering (ASE 2019). United States. 32 (6) (pp B121), 2019. Date of Publication: June 2019.
[Conference Abstract]
AN: 2003682982
Publisher
Mosby Inc.

Link to the Ovid Full Text or citation:
[Click here for full text options]

Link to the External Link Resolver:
[SFX Link]

120.

Ability of artificial intelligence to diagnose coronary artery stenosis using hybrid images of coronary computed tomography angiography and myocardial perfusion SPECT.
Yoneyama H., Nakajima K., Taki J., Wakabayashi H., Konishi T., Okuda K., Shiburani T., Onoguchi M., Kinuya S.
Embase
European Journal of Nuclear Medicine and Molecular Imaging. Conference: 32nd Annual Congress of the European Association of Nuclear Medicine, EANM 2019. Spain. 46 (1 Supplement 1) (pp S103-S104), 2019. Date of Publication: October 2019.
[Conference Abstract]
AN: 629701008
Publisher
Springer Berlin Heidelberg

Link to the Ovid Full Text or citation:
[Click here for full text options]

Link to the External Link Resolver:
SFX Link

121.

Automatic alignment of CZT myocardial perfusion SPECT and external non-contrast CT by deep-learning model and dynamic data generation.
Ko C.-L., Cheng M.-F., Yen R.-F., Chen C.-M., Lee W.-J., Wang T.-D.
Embase
Journal of Nuclear Medicine. Conference: 2019 Annual Meeting of the Society of Nuclear Medicine and Molecular Imaging, SNMMI 2019. United States. 60 (Supplement 1) (no pagination), 2019. Date of Publication: May 2019.
[Conference Abstract]
AN: 629437253
Publisher
Society of Nuclear Medicine Inc.

Link to the Ovid Full Text or citation:
Click here for full text options

Link to the External Link Resolver:
SFX Link

122.

Artificial Intelligence tools for the evaluation of myocardial perfusion imaging.
De Souza-Filho E.M., De Amorim Fernandes F., Seixas F.L., Gismondi R.A., Mesquita C.
Embase
Journal of Nuclear Medicine. Conference: 2019 Annual Meeting of the Society of Nuclear Medicine and Molecular Imaging, SNMMI 2019. United States. 60 (Supplement 1) (no pagination), 2019. Date of Publication: May 2019.
[Conference Abstract]
AN: 629437560
Publisher
Society of Nuclear Medicine Inc.

Link to the Ovid Full Text or citation:
Click here for full text options

Link to the External Link Resolver:
SFX Link

123.

Development of a deep learning-based interpretation model for brain perfusion SPECT leveraging unstructured reading reports.

Ryoo H.G., Choi H., Wong T.H., Kang S.K., Lee J.S., Lee D.S.
Embase
Journal of Nuclear Medicine. Conference: 2019 Annual Meeting of the Society of Nuclear Medicine and Molecular Imaging, SNMMI 2019. United States. 60 (Supplement 1) (no pagination), 2019. Date of Publication: May 2019.
[Conference Abstract]
AN: 629438495
Publisher
Society of Nuclear Medicine Inc.

Link to the Ovid Full Text or citation:
[Click here for full text options]

Link to the External Link Resolver:
[SFX Link]

124.

Deep learning analysis of Tl-201 myocardial perfusion imaging for improve diagnostic accuracy.
Chiu C.-H., Cheng C.-Y., Tseng T.-W., Chen I.-J., Yin Y.-P.
Embase
Journal of Nuclear Medicine. Conference: 2019 Annual Meeting of the Society of Nuclear Medicine and Molecular Imaging, SNMMI 2019. United States. 60 (Supplement 1) (no pagination), 2019. Date of Publication: May 2019.
[Conference Abstract]
AN: 629438253
Publisher
Society of Nuclear Medicine Inc.

Link to the Ovid Full Text or citation:
[Click here for full text options]

Link to the External Link Resolver:
[SFX Link]

125.

Development of transfer learning datasets using realistic simulation of myocardial perfusion SPECT images for a deep learning model.
Lee T.-S., Xu J., Tsui B.
Embase
Journal of Nuclear Medicine. Conference: 2019 Annual Meeting of the Society of Nuclear Medicine and Molecular Imaging, SNMMI 2019. United States. 60 (Supplement 1) (no pagination), 2019. Date of Publication: May 2019.
[Conference Abstract]
AN: 629438603
Publisher
Society of Nuclear Medicine Inc.

Link to the Ovid Full Text or citation:
Click here for full text options

Link to the External Link Resolver:
SFX Link

126.

Predicting cerebrovascular reserve: A brain stress test without drugs.
Chen D.Y., Ishii Y., Fan A.P., Zaharchuk G.
Embase
Journal of Cerebral Blood Flow and Metabolism. Conference: 29th International Symposium on Cerebral Blood Flow, Metabolism and Function and the 14th International Conference on Quantification of Brain Function with PET. Japan. 39 (1 Supplement) (pp 611-612), 2019. Date of Publication: July 2019.
[Conference Abstract]
AN: 629098226
Publisher
SAGE Publications Ltd

Link to the Ovid Full Text or citation:
Click here for full text options

Link to the External Link Resolver:
SFX Link

127.

Multiplatform molecular profiling reveals intratumor heterogeneity in ependymoma.
John Liu S., Magill S., Vasudevan H., Hilz S., Daggubati V., Villanueva-Meyer J., Choudhury A., Ferris S., Orr B., Bush N.A.O., Bollen A., McDermott M., Costello J., Raleigh D.
Embase
Neuro-Oncology. Conference: 5th Pediatric Neuro-Oncology Basic and Translational Research Conference. United States. 21 (Supplement 2) (pp AA77-AA78), 2019. Date of Publication: April 2019.
[Conference Abstract]
AN: 628911098
Publisher
Oxford University Press

Link to the Ovid Full Text or citation:
Click here for full text options

Link to the External Link Resolver:
SFX Link

128.

Cardiac SPECT data analysis using graph-based convolutional neural networks.
Spier N., Rischpler C., Rupprecht C., Navab N., Baust M., Nekolla S.G.
Embase
NuklearMedizin. Conference: 57. Jahrestagung der Deutschen Gesellschaft fur Nuklearmedizin. Germany. 58 (2) (pp 117-118), 2019. Date of Publication: March 2019.
[Conference Abstract]
AN: 628679723
Publisher
Georg Thieme Verlag

Link to the Ovid Full Text or citation:
Click here for full text options

Link to the External Link Resolver:
SFX Link

129.

Machine learning allows easy predictor ranking in clinical stroke models.
Livne M., Madai V.I., Zihni E., Kossen T., Akay E.M., Galinovic I., Fiebach J.B., Frey D.
Embase
European Stroke Journal. Conference: 5th European Stroke Organisation Conference, ESOC 2019. Italy. 4 (Supplement 1) (pp 404), 2019. Date of Publication: May 2019.
[Conference Abstract]
AN: 628560731
Publisher
SAGE Publications Ltd

Link to the Ovid Full Text or citation:
Click here for full text options

Link to the External Link Resolver:
SFX Link

130.

Deep learning-based dual-energy computed tomography imaging.
Lv T., Zhao W., Zhao Q., Xing L., Zhang L., Chen Y.
Embase
International Journal of Computer Assisted Radiology and Surgery. Conference: 33rd International Congress and Exhibition of the Computer Assisted Radiology and Surgery, CARS 2019. France. 14 (Supplement 1) (pp S98-S99), 2019. Date of Publication: June 2019.
[Conference Abstract]

AN: 628423273
Publisher
Springer Verlag

Link to the Ovid Full Text or citation:
[Click here for full text options]

Link to the External Link Resolver:
[SFX Link]

131.

Uncovering hidden patterns in biological datasets to identify metabolic alterations caused by acute and sub-chronic DOX treatments.
Pino R., Cunha-Oliveira T., Carvalho F., Garcia R., Burgeiro A., Carvalho R.A., Oliveira P.J., Lourenco N.
Embase
European Journal of Clinical Investigation. Conference: 53rd Annual Scientific Meeting of the European Society for Clinical Investigation. Portugal. 49 (Supplement 1) (pp 85), 2019. Date of Publication: April 2019.
[Conference Abstract]
AN: 627696330
Publisher
Blackwell Publishing Ltd

Link to the Ovid Full Text or citation:
Click here for full text options

Link to the External Link Resolver:
SFX Link

132.

Improving myocardial ischaemia diagnostic accuracy using myocardial perfusion and CT fusion imaging based 3D-OSEM data.
Nakamura Y.
Embase
Journal of Medical Radiation Sciences. Conference: Australian Society of Medical Imaging and Radiation Therapy's 14th National Conference and 22nd Asia-Australasia Conference of Radiological Technologists Conference, ASMIRT and AACRT 2019. Australia. 66 (Supplement 1) (pp 128), 2019. Date of Publication: March 2019.
[Conference Abstract]
AN: 626940075
Publisher
Wiley Blackwell

Link to the Ovid Full Text or citation:
Click here for full text options

Link to the External Link Resolver:
SFX Link

133.

Ees Estimation Using Machine Learning Approach during Ex Vivo Heart Perfusion to Predict Early Graft Survival: A Preliminary Study in Porcine Model.
Xin L., Xiao W., Yao W., Liu Y., Ribeiro R.V., Gellner B., Alvarez J., Yu F., Paradiso E., Adamson M., Bissoondath V., Hiansen J., Meineri M., Sun Y., Badiwala M.
Embase
Journal of Heart and Lung Transplantation. Conference: ISHLT 39th Annual Meeting and Scientific Sessions. United States. 38 (4 Supplement) (pp S242), 2019. Date of Publication: April 2019.
[Conference Abstract]
AN: 2001696005
Publisher
Elsevier USA

Link to the Ovid Full Text or citation:
Click here for full text options

Link to the External Link Resolver:
SFX Link

134.

Deep learning in PET myocardial perfusion imaging: A study on cardiovascular event prediction.
Eduardo J.-O.L., Knol R.J.J., Octavio M.-M., Van Der Zant F.M., Knuuti J.
Embase
Revista Mexicana de Cardiologia. Conference: Congreso Anual de Cardiologia, CADECI 2019. Mexico. 30 (Supplement 1) (pp S9), 2019. Date of Publication: January - March 2019.
[Conference Abstract]
AN: 626707959
Publisher
Asociacion Nacional de Cardiologos de Mexico

Link to the Ovid Full Text or citation:
Click here for full text options

Link to the External Link Resolver:
SFX Link

135.

Machine learning in cardiovascular magnetic resonance: Basic concepts and applications.
Leiner T., Rueckert D., Suinesiaputra A., Baessler B., Nezafat R., Isgum I., Young A.A.
Embase
Journal of Cardiovascular Magnetic Resonance. 21 (1) (no pagination), 2019. Article Number: 61. Date of Publication: 07 Oct 2019.
[Review]
AN: 629512009
Publisher
BioMed Central Ltd. (E-mail: info@biomedcentral.com)

Link to the Ovid Full Text or citation:
[Click here for full text options]

Link to the External Link Resolver:
[SFX Link]

136.

Seeing a tree through the forest: Precision medicine tools can enhance donor allocation in heart transplantation.
Vasanthan V., Fedak P.W.M.
Embase
Journal of Thoracic and Cardiovascular Surgery. 155 (4) (pp 1591-1592), 2018. Date of Publication: April 2018.
[Editorial]
AN: 620081905
Publisher
Mosby Inc. (E-mail: customerservice@mosby.com)

Link to the Ovid Full Text or citation:
[Click here for full text options]

Link to the External Link Resolver:
[SFX Link]

137.

Can a Machine Learn Better Than Humans? *.
Shaw L.J.
Embase
JACC: Cardiovascular Imaging. 11 (7) (pp 1010-1011), 2018. Date of Publication: July 2018.
[Editorial]
AN: 618870327
Publisher
Elsevier Inc. (E-mail: usjcs@elsevier.com)

Link to the Ovid Full Text or citation:
Click here for full text options

Link to the External Link Resolver:
SFX Link

138.

Fully automated analysis of perfusion data: The rise of the machines.
Sanghani R.M., Doukky R.
Embase
Journal of Nuclear Cardiology. 25 (4) (pp 1361-1363), 2018. Date of Publication: 01 Aug 2018.
[Editorial]
AN: 615666316
Publisher
Springer New York LLC (E-mail: barbara.b.bertram@gsk.com)

Link to the Ovid Full Text or citation:
Click here for full text options

Link to the External Link Resolver:
SFX Link

139.

Automatic determination of cardiovascular risk by CT attenuation correction maps in Rb-82 PET/CT.
Isgum I., de Vos B.D., Wolterink J.M., Dey D., Berman D.S., Rubeaux M., Leiner T., Slomka P.J.
Embase
Journal of Nuclear Cardiology. 25 (6) (pp 2133-2142), 2018. Date of Publication: 15 Dec 2018.
[Article]
AN: 615238320
Publisher
Springer New York LLC (E-mail: barbara.b.bertram@gsk.com)

Link to the Ovid Full Text or citation:
Click here for full text options

Link to the External Link Resolver:
SFX Link

140.

Prognostic Value of Combined Clinical and Myocardial Perfusion Imaging Data Using

Machine Learning.
Betancur J., Otaki Y., Motwani M., Fish M.B., Lemley M., Dey D., Gransar H., Tamarappoo B., Germano G., Sharir T., Berman D.S., Slomka P.J.
Embase
JACC: Cardiovascular Imaging. 11 (7) (pp 1000-1009), 2018. Date of Publication: July 2018.
[Article]
AN: 618870700
Publisher
Elsevier Inc. (E-mail: usjcs@elsevier.com)

Link to the Ovid Full Text or citation:
Click here for full text options

Link to the External Link Resolver:
SFX Link

141.

Incremental role of resting myocardial computed tomography perfusion for predicting physiologically significant coronary artery disease: A machine learning approach.
Han D., Lee J.H., Rizvi A., Gransar H., Baskaran L., Schulman-Marcus J., o Hartaigh B., Lin F.Y., Min J.K.
Embase
Journal of Nuclear Cardiology. 25 (1) (pp 223-233), 2018. Date of Publication: 01 Feb 2018.
[Article]
AN: 614917133
Publisher
Springer New York LLC (E-mail: barbara.b.bertram@gsk.com)

Link to the Ovid Full Text or citation:
Click here for full text options

Link to the External Link Resolver:
SFX Link

142.

Towards an automated multimodal clinical decision support system at the post anesthesia care unit.
Olsen R.M., Aasvang E.K., Meyhoff C.S., Dissing Sorensen H.B.
Embase
Computers in Biology and Medicine. 101 (pp 15-21), 2018. Date of Publication: 1 October 2018.
[Article]
AN: 2001005577
Publisher
Elsevier Ltd

Link to the Ovid Full Text or citation:
Click here for full text options

Link to the External Link Resolver:
SFX Link

143.

CT coronary imaging-a fast evolving world.
Rajiah P., Abbara S.
Embase
QJM. 111 (9) (pp 595-604), 2018. Date of Publication: 2018.
[Review]
AN: 626456368
Publisher
Oxford University Press

Link to the Ovid Full Text or citation:
Click here for full text options

Link to the External Link Resolver:
SFX Link

144.

Hemodynamic Instability and Cardiovascular Events after Traumatic Brain Injury Predict Outcome after Artifact Removal with Deep Belief Network Analysis.
Kim H., Lee S.-B., Son Y., Czosnyka M., Kim D.-J.
Embase
Journal of Neurosurgical Anesthesiology. 30 (4) (pp 347-353), 2018. Date of Publication: 01 Oct 2018.
[Article]
AN: 624146738
Publisher
Lippincott Williams and Wilkins (E-mail: kathiest.clai@apta.org)

Link to the Ovid Full Text or citation:
Click here for full text options

Link to the External Link Resolver:
SFX Link

145.

Raising awareness: A year for multi-modality cardiac imaging.
Reyes E.

Embase
Journal of Nuclear Cardiology. 25 (4) (pp 1061-1062), 2018. Date of Publication: 01 Aug 2018.
[Editorial]
AN: 622666785
Publisher
Springer New York LLC (E-mail: barbara.b.bertram@gsk.com)

Link to the Ovid Full Text or citation:
Click here for full text options

Link to the External Link Resolver:
SFX Link

146.

Myocardial perfusion quantification using simultaneously acquired 13NH3-ammonia PET and dynamic contrast-enhanced MRI in patients at rest and stress.
Kunze K.P., Nekolla S.G., Rischpler C., Zhang S.H., Hayes C., Langwieser N., Ibrahim T., Laugwitz K.-L., Schwaiger M.
Embase
Magnetic Resonance in Medicine. 80 (6) (pp 2641-2654), 2018. Date of Publication: December 2018.
[Article]
AN: 621803121
Publisher
John Wiley and Sons Inc. (P.O.Box 18667, Newark NJ 07191-8667, United States)

Link to the Ovid Full Text or citation:
Click here for full text options

Link to the External Link Resolver:
SFX Link

147.

Deep Learning for Prediction of Obstructive Disease From Fast Myocardial Perfusion SPECT: A Multicenter Study.
Betancur J., Commandeur F., Motlagh M., Sharir T., Einstein A.J., Bokhari S., Fish M.B., Ruddy T.D., Kaufmann P., Sinusas A.J., Miller E.J., Bateman T.M., Dorbala S., Di Carli M., Germano G., Otaki Y., Tamarappoo B.K., Dey D., Berman D.S., Slomka P.J.
Embase
JACC: Cardiovascular Imaging. 11 (11) (pp 1654-1663), 2018. Date of Publication: November 2018.
[Article]
AN: 621224319
Publisher
Elsevier Inc. (E-mail: usjcs@elsevier.com)

Link to the Ovid Full Text or citation:
Click here for full text options

Link to the External Link Resolver:
SFX Link

148.

Temporal Performance of Laplacian Eigenmaps and 3D Conduction Velocity in Detecting Ischemic Stress.
Good W.W., Erem B., Zenger B., Coll-Font J., Brooks D.H., MacLeod R.S.
Embase
Journal of Electrocardiology. 51 (6 Supplement) (pp S116-S120), 2018. Date of Publication: November - December 2018.
[Article]
AN: 2001030952
Publisher
Churchill Livingstone Inc.

Link to the Ovid Full Text or citation:
Click here for full text options

Link to the External Link Resolver:
SFX Link

149.

Central nervous system neuroplasticity and the sensitization of hypertension.
Johnson A.K., Xue B.
Embase
Nature Reviews Nephrology. 14 (12) (pp 750-766), 2018. Date of Publication: 01 Dec 2018.
[Review]
AN: 624544534
Publisher
Nature Publishing Group (Houndmills, Basingstoke, Hampshire RG21 6XS, United Kingdom)

Link to the Ovid Full Text or citation:
Click here for full text options

Link to the External Link Resolver:
SFX Link

150.

Could Deep Learning Change Our Working Lives?*.
Sabharwal N.K.
Embase
JACC: Cardiovascular Imaging. 11 (11) (pp 1664-1665), 2018. Date of Publication: November 2018.
[Editorial]
AN: 621224293
Publisher
Elsevier Inc. (E-mail: usjcs@elsevier.com)

Link to the Ovid Full Text or citation:
Click here for full text options

Link to the External Link Resolver:
SFX Link

151.

COPD biomarkers and phenotypes: Opportunities for better outcomes with precision imaging.
Washko G.R., Parraga G.
Embase
European Respiratory Journal. 52 (5) (no pagination), 2018. Article Number: 1801570. Date of Publication: 2018.
[Article]
AN: 2002034434
Publisher
European Respiratory Society (E-mail: info@ersnet.org)

Link to the Ovid Full Text or citation:
Click here for full text options

Link to the External Link Resolver:
SFX Link

152.

Cardiovascular assessment by imaging photoplethysmography-a review.
Zaunseder S., Trumpp A., Wedekind D., Malberg H.
Embase
Biomedizinische Technik. 63 (5) (pp 529-535), 2018. Date of Publication: 25 Oct 2018.
[Article]
AN: 622660556
Publisher
De Gruyter (E-mail: peter.golla@degruyter.com)

Link to the Ovid Full Text or citation:
Click here for full text options

Link to the External Link Resolver:
SFX Link

153.

Quantitative myocardial first-pass cardiovascular magnetic resonance perfusion imaging using hyperpolarized [1-13C] pyruvate.
Fuetterer M., Busch J., Traechtler J., Wespi P., Peereboom S.M., Sauer M., Lipiski M., Fleischmann T., Cesarovic N., Stoeck C.T., Kozerke S.
Embase
Journal of Cardiovascular Magnetic Resonance. 20 (1) (no pagination), 2018. Article Number: 73. Date of Publication: 12 Nov 2018.
[Article]
AN: 624812291
Publisher
BioMed Central Ltd. (E-mail: info@biomedcentral.com)

Link to the Ovid Full Text or citation:
Click here for full text options

Link to the External Link Resolver:
SFX Link

154.

Fully automatic myocardial segmentation of contrast echocardiography sequence using random forests guided by shape model.
Li Y., Ho C.P., Toulemonde M., Chahal N., Senior R., Tang M.-X.
Embase
IEEE Transactions on Medical Imaging. 37 (5) (pp 1081-1091), 2018. Date of Publication: May 2018.
[Article]
AN: 618697308
Publisher
Institute of Electrical and Electronics Engineers Inc.

Link to the Ovid Full Text or citation:
Click here for full text options

Link to the External Link Resolver:
SFX Link

155.

Automated interpretation of myocardial perfusion images with multilayer perceptron network as a decision support system.

Eftekhari M., Abbasi M., Tarafdari A., Emami-Ardekani A., Farzanefar S., Kalantari F., Fallahi B., Fard-Esfahani A., Beiki D., Naseri M., Saghari M.
Embase
Journal of Medical Imaging and Health Informatics. 8 (9) (pp 1844-1849), 2018. Date of Publication: December 2018.
[Article]
AN: 625911821
Publisher
American Scientific Publishers (E-mail: order@aspbs.com)

Link to the Ovid Full Text or citation:
Click here for full text options

Link to the External Link Resolver:
SFX Link

156.

Cardiovascular imaging 2017 in the International Journal of Cardiovascular Imaging.
Reiber J.H.C., Alaiti A., Bezerra H.G., De Sutter J., Schoenhagen P., Stillman A.E., Van de Veire N.R.L.
Embase
International Journal of Cardiovascular Imaging. 34 (6) (pp 833-848), 2018. Date of Publication: 01 Jun 2018.
[Editorial]
AN: 621664646
Publisher
Springer Netherlands (E-mail: rbk@louisiana.edu)

Link to the Ovid Full Text or citation:
Click here for full text options

Link to the External Link Resolver:
SFX Link

157.

Diagnostic performance of an artificial intelligence-driven cardiac-structured reporting system for myocardial perfusion SPECT imaging.
Garcia E.V., Klein J.L., Moncayo V., Cooke C.D., Del'Aune C., Folks R., Moreiras L.V., Esteves F.
Embase
Journal of Nuclear Cardiology. (no pagination), 2018. Date of Publication: 2018.
[Article In Press]
AN: 623929613
Publisher
Springer New York LLC (E-mail: barbara.b.bertram@gsk.com)

Link to the Ovid Full Text or citation:

Click here for full text options

Link to the External Link Resolver:
SFX Link

158.

Artificial neural network retrained to detect myocardial ischemia using a Japanese multicenter database.
Nakajima K., Okuda K., Watanabe S., Matsuo S., Kinuya S., Toth K., Edenbrandt L.
Embase
Annals of Nuclear Medicine. 32 (5) (pp 303-310), 2018. Date of Publication: 01 Jun 2018.
[Article]
AN: 621089016
Publisher
Springer Tokyo (E-mail: orders@springer.jp)

Link to the Ovid Full Text or citation:
Click here for full text options

Link to the External Link Resolver:
SFX Link

159.

Qualitative angiographic and quantitative myocardial perfusion assessment using fluorescent cardiac imaging during graded coronary artery bypass stenosis.
Detter C., Russ D., Kersten J.F., Reichenspurner H., Wipper S.
Embase
International Journal of Cardiovascular Imaging. 34 (2) (pp 159-167), 2018. Date of Publication: 01 Feb 2018.
[Article]
AN: 617342194
Publisher
Springer Netherlands (E-mail: rbk@louisiana.edu)

Link to the Ovid Full Text or citation:
Click here for full text options

Link to the External Link Resolver:
SFX Link

160.

Scope of physiological and behavioural pain assessment techniques in children - A review.

Subramaniam S.D., Doss B., Chanderasekar L.D., Madhavan A., Rosary A.M.
Embase
Healthcare Technology Letters. 5 (4) (pp 124-129), 2018. Date of Publication: 2018.
[Article]
AN: 623636896
Publisher
Institution of Engineering and Technology (E-mail: journals@theiet.org)

Link to the Ovid Full Text or citation:
Click here for full text options

Link to the External Link Resolver:
SFX Link

161.

Application of Artificial Intelligence in Coronary Computed Tomography Angiography.

Selvarajah A., Bennamoun M., Playford D., Chow B.J.W., Dwivedi G.
Embase
Current Cardiovascular Imaging Reports. 11 (6) (no pagination), 2018. Article Number: 12. Date of Publication: 01 Jun 2018.
[Review]
AN: 622827104
Publisher
Current Medicine Group LLC 1 (E-mail: info@phl.cursci.com)

Link to the Ovid Full Text or citation:
Click here for full text options

Link to the External Link Resolver:
SFX Link

162.

Radionuclide Imaging in Decision-Making for Coronary Revascularization in Stable Ischemic Heart Disease.
Wiefels C., Erthal F., deKemp R.A., Chong A.Y., Mielniczuk L.M., Mesquita C.T., Beanlands R.S.B., Promislow S.
Embase
Current Cardiovascular Imaging Reports. 11 (8) (no pagination), 2018. Article Number: 20. Date of Publication: 01 Aug 2018.
[Review]
AN: 622791471
Publisher
Current Medicine Group LLC 1 (E-mail: info@phl.cursci.com)

Link to the Ovid Full Text or citation:
Click here for full text options

Link to the External Link Resolver:
SFX Link

163.

PET myocardial perfusion quantification: anatomy of a spreading functional technique.
Juarez-Orozco L.E., Cruz-Mendoza J.R., Guinto-Nishimura G.Y., Walls-Laguarda L., Casares-Echeverria L.J., Meave-Gonzalez A., Knuuti J., Alexanderson E.
Embase
Clinical and Translational Imaging. 6 (1) (pp 47-60), 2018. Date of Publication: 01 Feb 2018.
[Review]
AN: 620888937
Publisher
Springer-Verlag Italia s.r.l. (E-mail: springer@springer.it)

Link to the Ovid Full Text or citation:
Click here for full text options

Link to the External Link Resolver:
SFX Link

164.

New Trends in Quantitative Nuclear Cardiology Methods.
Gomez J., Doukky R., Germano G., Slomka P.
Embase
Current Cardiovascular Imaging Reports. 11 (1) (no pagination), 2018. Article Number: 1. Date of Publication: 01 Jan 2018.
[Review]
AN: 620250858
Publisher
Current Medicine Group LLC 1 (E-mail: info@phl.cursci.com)

Link to the Ovid Full Text or citation:
Click here for full text options

Link to the External Link Resolver:
SFX Link

165.

Wearable technology for compensatory reserve to sense hypovolemia.
Convertino V.A., Sawka M.N.
Embase

Journal of applied physiology (Bethesda, Md. : 1985). 124 (2) (pp 442-451), 2018.
Date of Publication: 01 Feb 2018.
[Review]
AN: 629414127
Publisher
NLM (Medline)

Link to the Ovid Full Text or citation:
[Click here for full text options]

Link to the External Link Resolver:
[SFX Link]

166.

High-Frame-Rate Contrast Echocardiography Using Diverging Waves: Initial In Vitro and In Vivo Evaluation.
Toulemonde M., Li Y., Lin S., Cordonnier F., Butler M., Duncan W.C., Eckersley R.J., Sboros V., Tang M.-X.
Embase
IEEE transactions on ultrasonics, ferroelectrics, and frequency control. 65 (12) (pp 2212-2221), 2018. Date of Publication: 01 Dec 2018.
[Article]
AN: 625617441
Publisher
NLM (Medline)

Link to the Ovid Full Text or citation:
[Click here for full text options]

Link to the External Link Resolver:
[SFX Link]

167.

World Congress of Cardiology & Cardiovascular Health 2018.
Anonymous
Embase
Global Heart. Conference: World Congress of Cardiology & Cardiovascular Health 2018. United Arab Emirates. 13 (4) (no pagination), 2018. Date of Publication: December 2018.
[Conference Review]
AN: 2001540459
Publisher
Elsevier B.V.

Link to the Ovid Full Text or citation:
[Click here for full text options]

Link to the External Link Resolver:
SFX Link

168.

Reprogramming the stem cell switch for cardiac regeneration-experimental and clinical analysis of aberrant LNK/SH2B3.
Steinhoff G., Wolfien M., Asahara T.
Embase
Circulation Research. Conference: American Heart Association's Scientific Sessions, AHA 2018. United States. 123 (12) (pp e80), 2018. Date of Publication: December 2018.
[Conference Abstract]
AN: 627914248
Publisher
Lippincott Williams and Wilkins

Link to the Ovid Full Text or citation:
Click here for full text options

Link to the External Link Resolver:
SFX Link

169.

Machine learning improves the long-term prognostic value of sequential cardiac PET/CT.
Juarez-Orozco L.E., Maaniitty T., Martinez-Manzanera O., Saraste A., Knuuti J.
Embase
European Heart Journal. Conference: European Society of Cardiology Congress, ESC 2018. Germany. 39 (Supplement 1) (pp 627), 2018. Date of Publication: August 2018.
[Conference Abstract]
AN: 627247699
Publisher
Oxford University Press

Link to the Ovid Full Text or citation:
Click here for full text options

Link to the External Link Resolver:
SFX Link

170.

The prognostic value of deep learning in PET myocardial perfusion for cardiovascular events.

Juarez-Orozco L.E., Knol R.J.J., Martinez-Manzanera O., Van Der Zant F.M., Knuuti J.
Embase
European Heart Journal. Conference: European Society of Cardiology Congress, ESC 2018. Germany. 39 (Supplement 1) (pp 269), 2018. Date of Publication: August 2018.
[Conference Abstract]
AN: 627214819
Publisher
Oxford University Press

Link to the Ovid Full Text or citation:
Click here for full text options

Link to the External Link Resolver:
SFX Link

171.

Pulse deficit as a new, innovative, non-invasive measure to risk-stratify atrial fibrillation patients.
Portnoy Y.-E.M., Richardson L.D.
Embase
Circulation. Conference: 2018 American Heart Association Scientific Sessions. United States. 138 (Supplement 1) (no pagination), 2018. Date of Publication: November 2018.
[Conference Abstract]
AN: 626955819
Publisher
Lippincott Williams and Wilkins

Link to the Ovid Full Text or citation:
Click here for full text options

Link to the External Link Resolver:
SFX Link

172.

Absolute myocardial perfusion and fow reserve with 99mTc-teboroxime dynamic SPECT in a porcine model.
Degtiarova G., Claus P., Verberne H., Wu M., Vanbilloen B., Bormans G., Gheysens O.
Embase
European Journal of Nuclear Medicine and Molecular Imaging. Conference: 31st Annual Congress of the European Association of Nuclear Medicine, EANM 2018. Germany. 45 (Supplement 1) (pp S636), 2018. Date of Publication: October 2018.
[Conference Abstract]
AN: 626435524
Publisher

Springer Berlin Heidelberg

Link to the Ovid Full Text or citation:
Click here for full text options

Link to the External Link Resolver:
SFX Link

173.

Effects of re-training on neural network diagnosis to detect ischemia in myocardial perfusion imaging: Comparison of two modes of trainings.
Nakajima K., Okuda K., Watanabe S., Matsuo S., Wakabayashi H., Kinuya S., Edenbrandt L.
Embase
European Journal of Nuclear Medicine and Molecular Imaging. Conference: 31st Annual Congress of the European Association of Nuclear Medicine, EANM 2018. Germany. 45 (Supplement 1) (pp S93), 2018. Date of Publication: October 2018.
[Conference Abstract]
AN: 626433721
Publisher
Springer Berlin Heidelberg

Link to the Ovid Full Text or citation:
Click here for full text options

Link to the External Link Resolver:
SFX Link

174.

Creation of specific normal databases for perfusion quantification of low-dose myocardial SPECT studies.
Scabbio C., Sole A.D., Capozza A., Malaspina S., Selvaggi C., Lucignani G., Lecchi M.
Embase
Physica Medica. Conference: 10th National Congress of the Associazione Italiana di Fisica Medica. Italy. 56 (Supplement 2) (pp 112-113), 2018. Date of Publication: December 2018.
[Conference Abstract]
AN: 2001384524
Publisher
Associazione Italiana di Fisica Medica

Link to the Ovid Full Text or citation:
Click here for full text options

Link to the External Link Resolver:
SFX Link

175.

Identifying Mediate Adverse Events Through Deep Learning and PET Myocardial Perfusion Imaging.
Juarez-Orozco L.E., Knol R.J., Martinez-Manzanera O., van der Zant F.M., Knuuti J.
Embase
Global Heart. Conference: World Congress of Cardiology & Cardiovascular Health 2018. United Arab Emirates. 13 (4) (pp 373), 2018. Date of Publication: December 2018.
[Conference Abstract]
AN: 2001223503
Publisher
Elsevier B.V.

Link to the Ovid Full Text or citation:
Click here for full text options

Link to the External Link Resolver:
SFX Link

176.

Externally validated deep learning improves per-vessel prediction of obstructive coronary artery disease from upright and supine spect MPI: A multicenter study.
Betancur J.A., Hu L., Sharir T., Einstein A.J., Fish M.B., Ruddy T.D., Kaufmann P., Sinusas A.J., Bateman T.M., Dorbala S., Berman D.S., Slomka P.J.
Embase
Journal of Nuclear Cardiology. Conference: 23rd Annual Scientific Session of the American Society of Nuclear Cardiology, ASNC 2018. United States. 25 (4) (pp 1458), 2018. Date of Publication: August 2018.
[Conference Abstract]
AN: 623902043
Publisher
Springer New York LLC

Link to the Ovid Full Text or citation:
Click here for full text options

Link to the External Link Resolver:
SFX Link

177.

Automatic classification of myocardial perfusion abnormalities from nuclear cardiac spect images using a neural network model.
Zhang Y., Cheng K., Chen P., Liu Y.

Embase
Journal of Nuclear Cardiology. Conference: 23rd Annual Scientific Session of the American Society of Nuclear Cardiology, ASNC 2018. United States. 25 (4) (pp 1456), 2018. Date of Publication: August 2018.
[Conference Abstract]
AN: 623902018
Publisher
Springer New York LLC

Link to the Ovid Full Text or citation:
Click here for full text options

Link to the External Link Resolver:
SFX Link

178.

Beyond stenosis: Plaque and ischemia assessments by coronary cta.
Berman D.S., Otaki Y., Friedman J., Hayes S., Thomson L., Eisenberg E., Rozanski A., Slomka P., Dey D., Tamarappoo B.K.
Embase
Cardiology (Switzerland). Conference: 23rd World Congress on Heart Disease International Academy of Cardiology Annual Scientific Sessions 2018. United States. 140 (Supplement 1) (pp 23), 2018. Date of Publication: 2018.
[Conference Abstract]
AN: 623414766
Publisher
S. Karger AG

Link to the Ovid Full Text or citation:
Click here for full text options

Link to the External Link Resolver:
SFX Link

179.

Radiomics analysis of clinical myocardial perfusion spect to predict coronary artery calcification.
Ashrafinia S., Dalaie P., Yan R., Ghazi P., Marcus C., Taghipour M., Huang P., Pomper M., Schindler T., Rahmim A.
Embase
Journal of Nuclear Medicine. Conference: Society of Nuclear Medicine and Molecular Imaging Annual Meeting, SNMMI 2018. United States. 59 (Supplement 1) (no pagination), 2018. Date of Publication: May 2018.
[Conference Abstract]
AN: 623022211
Publisher
Society of Nuclear Medicine Inc.

Link to the Ovid Full Text or citation:
Click here for full text options

Link to the External Link Resolver:
SFX Link

180.

Machine learning predicts early coronary revascularization after fast myocardial SPECT: Results from multicenter REFINE SPECT registry.
Hu L.-H., Betancur J., Sharir T., Einstein A., Fish M., Ruddy T., Kaufmann P., Sinusas A., Miller E., Bateman T., Dorbala S., Carli M., Germano G., Otaki Y., Tamarappoo B., Dey D., Berman D., Slomka P.
Embase
Journal of Nuclear Medicine. Conference: Society of Nuclear Medicine and Molecular Imaging Annual Meeting, SNMMI 2018. United States. 59 (Supplement 1) (no pagination), 2018. Date of Publication: May 2018.
[Conference Abstract]
AN: 623022140
Publisher
Society of Nuclear Medicine Inc.

Link to the Ovid Full Text or citation:
Click here for full text options

Link to the External Link Resolver:
SFX Link

181.

The diagnostic accuracy of machine learning from stress only fast-MPS.
Eisenberg E., Betancur J., Hu L.-H., Sharir T., Einstein A., Ruddy T., Kaufmann P., Sinusas A., Miller E., Bateman T., Dorbala S., Di Carli M., Germano G., Otaki Y., Tamarappoo B., Dey D., Berman D., Slomka P.
Embase
Journal of Nuclear Medicine. Conference: Society of Nuclear Medicine and Molecular Imaging Annual Meeting, SNMMI 2018. United States. 59 (Supplement 1) (no pagination), 2018. Date of Publication: May 2018.
[Conference Abstract]
AN: 623022009
Publisher
Society of Nuclear Medicine Inc.

Link to the Ovid Full Text or citation:
Click here for full text options

Link to the External Link Resolver:
SFX Link

182.

Use of deep convolutional neural network-based features for detection of cardiac sarcoidosis from polar map.
Togo R., Hirata K., Manabe O., Ohira H., Tsujino I., Ogawa T., Haseyama M., Shiga T.
Embase
Journal of Nuclear Medicine. Conference: Society of Nuclear Medicine and Molecular Imaging Annual Meeting, SNMMI 2018. United States. 59 (Supplement 1) (no pagination), 2018. Date of Publication: May 2018.
[Conference Abstract]
AN: 623021931
Publisher
Society of Nuclear Medicine Inc.

Link to the Ovid Full Text or citation:
Click here for full text options

Link to the External Link Resolver:
SFX Link

183.

Automatic deep learning analysis of upright-supine high-speed SPECT myocardial perfusion imaging for prediction of obstructive coronary artery disease: A multicenter study.
Betancur J., Hu L.-H., Commandeur F., Sharir T., Einstein A., Bokhari S., Mathews F., Ruddy T., Kaufmann P., Sinusas A., Miller E., Bateman T., Dorbala S., Di Carli M., Germano G., Otaki Y., Tamarappoo B., Dey D., Berman D., Slomka P.
Embase
Journal of Nuclear Medicine. Conference: Society of Nuclear Medicine and Molecular Imaging Annual Meeting, SNMMI 2018. United States. 59 (Supplement 1) (no pagination), 2018. Date of Publication: May 2018.
[Conference Abstract]
AN: 623021917
Publisher
Society of Nuclear Medicine Inc.

Link to the Ovid Full Text or citation:
Click here for full text options

Link to the External Link Resolver:
SFX Link

184.

Improved myocardial perfusion PET imaging using artificial neural networks.

Wang X., Yang B., Gao X., Tang J.
Embase
Journal of Nuclear Medicine. Conference: Society of Nuclear Medicine and Molecular Imaging Annual Meeting, SNMMI 2018. United States. 59 (Supplement 1) (no pagination), 2018. Date of Publication: May 2018.
[Conference Abstract]
AN: 623021719
Publisher
Society of Nuclear Medicine Inc.

Link to the Ovid Full Text or citation:
Click here for full text options

Link to the External Link Resolver:
SFX Link

185.

Artificial neural network for prediction of coronary artery disease in patients with abnormal myocardial perfusion.
Kuznetsov V.A., Yaroslavskaya E.I., Krinochkin D.V., Teffenberg D.V., Dyachkov S.M.
Embase
European Journal of Heart Failure. Conference: Heart Failure 2018 and the 5th World Congress on Acute Heart Failure. Austria. 20 (Supplement 1) (pp 484), 2018. Date of Publication: May 2018.
[Conference Abstract]
AN: 622651723
Publisher
John Wiley and Sons Ltd

Link to the Ovid Full Text or citation:
Click here for full text options

Link to the External Link Resolver:
SFX Link

186.

Diagnostic accuracy of an artificial neural network compared with statistical quantitation of myocardial perfusion images: A Japanese multicenter study.
Nakajima K., Kudo T., Nakata T., Kiso K., Kasai T., Taniguchi Y., Matsuo S., Momose M., Nakagawa M., Sarai M., Hida S., Tanaka H., Yokoyama K., Okuda K., Edenbrandt L.
Embase
European Journal of Nuclear Medicine and Molecular Imaging. 44 (13) (pp 2280-2289), 2017. Date of Publication: 2017.
[Article]
AN: 621182750
Publisher

Springer Berlin Heidelberg (E-mail: service@springer.de)

Link to the Ovid Full Text or citation:
Click here for full text options

Link to the External Link Resolver:
SFX Link

187.

Neural network imaging to characterize brain injury in cardiac procedures: the emerging utility of connectomics.
Indja B., Fanning J.P., Maller J.J., Fraser J.F., Bannon P.G., Vallely M., Grieve S.M.
Embase
British Journal of Anaesthesia. 118 (5) (pp 680-688), 2017. Date of Publication: May 2017.
[Review]
AN: 2000794951
Publisher
Elsevier Ltd

Link to the Ovid Full Text or citation:
Click here for full text options

Link to the External Link Resolver:
SFX Link

188.

Validation of myocardial perfusion quantification by dynamic CT in an ex-vivo porcine heart model.
Pelgrim G.J., Das M., van Tuijl S., van Assen M., Prinzen F.W., Stijnen M., Oudkerk M., Wildberger J.E., Vliegenthart R.
Embase
International Journal of Cardiovascular Imaging. 33 (11) (pp 1821-1830), 2017. Date of Publication: 01 Nov 2017.
[Article]
AN: 616352496
Publisher
Springer Netherlands

Link to the Ovid Full Text or citation:
Click here for full text options

Link to the External Link Resolver:
SFX Link

189.

The effect of high count rates on cardiac perfusion quantification in a simultaneous PET-MR system using a cardiac perfusion phantom.
O' Doherty J., Chalampalakis Z., Schleyer P., Nazir M.S., Chiribiri A., Marsden P.K.
Embase
EJNMMI Physics. 4 (1) (no pagination), 2017. Article Number: 31. Date of Publication: 01 Dec 2017.
[Article]
AN: 620935549
Publisher
Springer International Publishing

Link to the Ovid Full Text or citation:
Click here for full text options

Link to the External Link Resolver:
SFX Link

190.

Machine Learning in Cardiac CT.
Landreth S.P., Spearman J.V.
Embase
Current Radiology Reports. 5 (10) (no pagination), 2017. Article Number: 49. Date of Publication: 01 Oct 2017.
[Review]
AN: 617642297
Publisher
Springer New York LLC (E-mail: barbara.b.bertram@gsk.com)

Link to the Ovid Full Text or citation:
Click here for full text options

Link to the External Link Resolver:
SFX Link

191.

More hemodynamic monitoring for personalized treatment in circulatory failure.
Van Der Horst I.C.C., Scheeren T.W.L.
Embase
Current Opinion in Critical Care. 23 (4) (pp 291-292), 2017. Date of Publication: 01 Aug 2017.
[Review]
AN: 617185255
Publisher
Lippincott Williams and Wilkins (E-mail: kathiest.clai@apta.org)

Link to the Ovid Full Text or citation:
Click here for full text options

Link to the External Link Resolver:
SFX Link

192.

What is the optimal anatomic location for coronary artery pressure measurement at CT-derived FFR?.
Solecki M., Kruk M., Demkow M., Schoepf U.J., Reynolds M.A., Wardziak L., Dzielinska Z., Spiewak M., Milosz-Wieczorek B., Malek L., Marczak M., Kepka C.
Embase
Journal of Cardiovascular Computed Tomography. 11 (5) (pp 397-403), 2017. Date of Publication: September 2017.
[Article]
AN: 618020438
Publisher
Elsevier Inc. (E-mail: usjcs@elsevier.com)

Link to the Ovid Full Text or citation:
Click here for full text options

Link to the External Link Resolver:
SFX Link

193.

Robust universal nonrigid motion correction framework for first-pass cardiac MR perfusion imaging.
Benovoy M., Jacobs M., Cheriet F., Dahdah N., Arai A.E., Hsu L.-Y.
Embase
Journal of Magnetic Resonance Imaging. 46 (4) (pp 1060-1072), 2017. Date of Publication: October 2017.
[Article]
AN: 614524460
Publisher
John Wiley and Sons Inc. (P.O.Box 18667, Newark NJ 07191-8667, United States)

Link to the Ovid Full Text or citation:
Click here for full text options

Link to the External Link Resolver:
SFX Link

194.

Multicenter evaluation of stress-first myocardial perfusion image triage by nuclear technologists and automated quantification.
Chaudhry W., Hussain N., Ahlberg A.W., Croft L.B., Fernandez A.B., Parker M.W., Swales H.H., Slomka P.J., Henzlova M.J., Duvall W.L.
Embase
Journal of Nuclear Cardiology. 24 (3) (pp 809-820), 2017. Date of Publication: 01 Jun 2017.
[Article]
AN: 606916226
Publisher
Springer New York LLC (E-mail: barbara.b.bertram@gsk.com)

Link to the Ovid Full Text or citation:
Click here for full text options

Link to the External Link Resolver:
SFX Link

195.

Myocardial perfusion cardiovascular magnetic resonance: optimized dual sequence and reconstruction for quantification.
Kellman P., Hansen M.S., Nielles-Vallespin S., Nickander J., Themudo R., Ugander M., Xue H.
Embase
Journal of Cardiovascular Magnetic Resonance. 19 (1) (no pagination), 2017. Article Number: 43. Date of Publication: 07 Apr 2017.
[Article]
AN: 615547541
Publisher
BioMed Central Ltd. (E-mail: info@biomedcentral.com)

Link to the Ovid Full Text or citation:
Click here for full text options

Link to the External Link Resolver:
SFX Link

196.

An empirical method for reducing variability and complexity of myocardial perfusion quantification by dual bolus cardiac MRI.
Chatterjee N., Benefield B.C., Harris K.R., Fluckiger J.U., Carroll T., Lee D.C.
Embase
Magnetic Resonance in Medicine. 77 (6) (pp 2347-2355), 2017. Date of Publication: June 2017.
[Article]
AN: 612977912
Publisher

John Wiley and Sons Inc. (P.O.Box 18667, Newark NJ 07191-8667, United States)

Link to the Ovid Full Text or citation:
Click here for full text options

Link to the External Link Resolver:
SFX Link

197.

Effect of Bayesian-penalized likelihood reconstruction on [13N]-NH3 rest perfusion quantification.
O' Doherty J., McGowan D.R., Abreu C., Barrington S.
Embase
Journal of Nuclear Cardiology. 24 (1) (pp 282-290), 2017. Date of Publication: 01 Feb 2017.
[Article]
AN: 611321033
Publisher
Springer New York LLC (E-mail: barbara.b.bertram@gsk.com)

Link to the Ovid Full Text or citation:
Click here for full text options

Link to the External Link Resolver:
SFX Link

198.

Coronary CT angiography-derived fractional flow reserve.
Tesche C., De Cecco C.N., Albrecht M.H., Duguay T.M., Bayer R.R., Litwin S.E., Steinberg D.H., Schoepf U.J.
Embase
Radiology. 285 (1) (pp 17-33), 2017. Date of Publication: October 2017.
[Article]
AN: 618419502
Publisher
Radiological Society of North America Inc. (820 Jorie Boulevard, Oak Brook IL 60523-2251, United States)

Link to the Ovid Full Text or citation:
Click here for full text options

Link to the External Link Resolver:
SFX Link

199.

Comprehensive Multi-Dimensional MRI for the Simultaneous Assessment of Cardiopulmonary Anatomy and Physiology.
Cheng J.Y., Zhang T., Alley M.T., Uecker M., Lustig M., Pauly J.M., Vasanawala S.S.

Embase
Scientific reports. 7 (1) (pp 5330), 2017. Date of Publication: 13 Jul 2017.
[Article]
AN: 625918899
Publisher
NLM (Medline)

Link to the Ovid Full Text or citation:
Click here for full text options

Link to the External Link Resolver:
SFX Link

200.

Automatic valve plane localization in myocardial perfusion SPECT/CT by machine learning: Anatomic and clinical validation.
Betancur J., Rubeaux M., Fuchs T.A., Otaki Y., Arnson Y., Slipczuk L., Benz D.C., Germano G., Dey D., Lin C.-J., Berman D.S., Kaufmann P.A., Slomka P.J.
Embase
Journal of Nuclear Medicine. 58 (6) (pp 961-967), 2017. Date of Publication: 01 Jun 2017.
[Article]
AN: 616623735
Publisher
Society of Nuclear Medicine Inc. (E-mail: MemberInfo@snm.org)

Link to the Ovid Full Text or citation:
Click here for full text options

Link to the External Link Resolver:
SFX Link

201.

Automatic localization of anatomical landmarks in cardiac MR perfusion using random forests.
Kim Y.-C., Chung Y., Choe Y.H.
Embase
Biomedical Signal Processing and Control. 38 (pp 370-378), 2017. Date of Publication: September 2017.
[Article]
AN: 617527077
Publisher

Elsevier Ltd

Link to the Ovid Full Text or citation:
Click here for full text options

Link to the External Link Resolver:
SFX Link

202.

Cardiac imaging: working towards fully-automated machine analysis & interpretation.
Slomka P.J., Dey D., Sitek A., Motwani M., Berman D.S., Germano G.
Embase
Expert Review of Medical Devices. 14 (3) (pp 197-212), 2017. Date of Publication: 04 Mar 2017.
[Review]
AN: 614828998
Publisher
Taylor and Francis Ltd (E-mail: info@expert-reviews.com)

Link to the Ovid Full Text or citation:
Click here for full text options

Link to the External Link Resolver:
SFX Link

203.

An integrated anti-arrhythmic target network of a Chinese medicine compound, Wenxin Keli, revealed by combined machine learning and molecular pathway analysis.
Wang T., Lu M., Du Q., Yao X., Zhang P., Chen X., Xie W., Li Z., Ma Y., Zhu Y.
Embase
Molecular bioSystems. 13 (5) (pp 1018-1030), 2017. Date of Publication: 02 May 2017.
[Article]
AN: 620630214

Link to the Ovid Full Text or citation:
Click here for full text options

Link to the External Link Resolver:
SFX Link

204.

Prediction of cardiac death after pharmacologicalmyocardial perfusion imaging using machine learning techniques.
Al Mallah M.H., Amjad A., Elshawi R., Sakr S.
Embase
European Heart Journal Cardiovascular Imaging. Conference: International Conference on Nuclear Cardiology and Cardiac CT, ICNC 2017. Austria. 18 (Supplement 1) (pp i51), 2017. Date of Publication: May 2017.
[Conference Abstract]
AN: 625228061
Publisher
Oxford University Press

Link to the Ovid Full Text or citation:
[Click here for full text options]

Link to the External Link Resolver:
[SFX Link]

205.

Prognostic value of quantitative absolutemyocardial stress perfusion in patients with chest pain and normal coronary arteries: A nitrogen-13 ammonia PET study.
Monroy Gonzalez A.G., Tio R.A., Alexanderson-Rosas E., Slart R.H.J.A.
Embase
European Heart Journal Cardiovascular Imaging. Conference: International Conference on Nuclear Cardiology and Cardiac CT, ICNC 2017. Austria. 18 (Supplement 1) (pp i47), 2017. Date of Publication: May 2017.
[Conference Abstract]
AN: 625227891
Publisher
Oxford University Press

Link to the Ovid Full Text or citation:
[Click here for full text options]

Link to the External Link Resolver:
[SFX Link]

206.

Myocardial stress perfusion with nitrogen-13 ammonia PET in patients with cardiac syndrome x and subsequent improve of symptoms in patients with transcutaneous electrical nerve stimulation.
Monroy Gonzalez A.G., Alexanderson-Rosas E.A., De Jongste M.J.L., Slart R.H.J.A., Tio R.A.
Embase
European Heart Journal Cardiovascular Imaging. Conference: International Conference on Nuclear Cardiology and Cardiac CT, ICNC 2017. Austria. 18 (Supplement 1) (pp i47), 2017. Date of Publication: May 2017.
[Conference Abstract]

AN: 625227869
Publisher
Oxford University Press

Link to the Ovid Full Text or citation:
Click here for full text options

Link to the External Link Resolver:
SFX Link

207.

The prognostic value of PETmyocardial perfusion reserve: A meta-analysis.
Juarez-Orozco L.E., Tio R.A., Alexanderson E., Dweck M., Vliegenthart R., El Moumni M., Gonzalez-Godinez I., Slart R.H.J.A.
Embase
European Heart Journal Cardiovascular Imaging. Conference: International Conference on Nuclear Cardiology and Cardiac CT, ICNC 2017. Austria. 18 (Supplement 1) (pp i46-i47), 2017. Date of Publication: May 2017.
[Conference Abstract]
AN: 625227848
Publisher
Oxford University Press

Link to the Ovid Full Text or citation:
Click here for full text options

Link to the External Link Resolver:
SFX Link

208.

Artificial neural network for coronary artery disease prediction in patients with abnormalmyocardial single photon emission computed tomography.
Yaroslavskaya E.I., Kuznetsov V.A., Teffenberg D.V., Krinochkin D.V., Dyachkov S.M.
Embase
European Heart Journal Cardiovascular Imaging. Conference: International Conference on Nuclear Cardiology and Cardiac CT, ICNC 2017. Austria. 18 (Supplement 1) (pp i92), 2017. Date of Publication: May 2017.
[Conference Abstract]
AN: 625227603
Publisher
Oxford University Press

Link to the Ovid Full Text or citation:
Click here for full text options

Link to the External Link Resolver:

SFX Link

209.

High-performance artificial neural network system for detecting ischemia usingmyocardial perfusion imaging: A multicenter project in Japan.
Nakajima K., Kiso K., Kudo T., Kasai T., Taniguchi Y., Matsuo S., Nakagawa M., Nakata T., Hida S., Tanaka H., Sarai M., Yokoyama K., Kondo C., Okuda K., Edenbrandt L.
Embase
European Heart Journal Cardiovascular Imaging. Conference: International Conference on Nuclear Cardiology and Cardiac CT, ICNC 2017. Austria. 18 (Supplement 1) (pp i40), 2017. Date of Publication: May 2017.
[Conference Abstract]
AN: 625227543
Publisher
Oxford University Press

Link to the Ovid Full Text or citation:
Click here for full text options

Link to the External Link Resolver:
SFX Link

210.

Transcription factors of the RPE play a role in choroid vascularization.
Cohen Y., Cohen H., Elkon R., Blinder P., Idelson M., Reubinoff B., Itzkovitz S., Ashery-Padan R.
Embase
Investigative Ophthalmology and Visual Science. Conference: 2017 Annual Meeting of the Association for Research in Vision and Ophthalmology, ARVO 2017. United States. 58 (8) (no pagination), 2017. Date of Publication: June 2017.
[Conference Abstract]
AN: 621487965
Publisher
Association for Research in Vision and Ophthalmology Inc.

Link to the Ovid Full Text or citation:
Click here for full text options

Link to the External Link Resolver:
SFX Link

211.

Improving the value of clinical variables in the assessment of cardiovascular risk

using Artificial Neural Networks.
Juarez-Orozco L.E., Knol R.J.J., Sanchez-Catasus C.A., Van Der Zant F.M., Knuuti J.
Embase
European Heart Journal. Conference: European Society of Cardiology, ESC Congress 2017. Spain. 38 (Supplement 1) (pp 227-228), 2017. Date of Publication: August 2017.
[Conference Abstract]
AN: 621235083
Publisher
Oxford University Press

Link to the Ovid Full Text or citation:
Click here for full text options

Link to the External Link Resolver:
SFX Link

212.

Combined FDG-PET and ultrafast ultrasound imaging of healthy and ischemic rat hearts in vivo.
Berthon B.
Embase
Molecular Imaging and Biology. Conference: 2017 World Molecular Imaging Congress, WMIC 2017. United States. 19 (1 Supplement 1) (pp S93), 2017. Date of Publication: 2017.
[Conference Abstract]
AN: 619448025
Publisher
Springer New York LLC

Link to the Ovid Full Text or citation:
Click here for full text options

Link to the External Link Resolver:
SFX Link

213.

Evaluation of diagnostic ability of an artificial neural network for detecting ischemia in myocardial perfusion imaging.
Yoneyama T., Nakajima K., Tsuji S., Yokoyama K., Michigishi T.
Embase
European Journal of Nuclear Medicine and Molecular Imaging. Conference: 30th Annual Congress of the European Association of Nuclear Medicine, EANM 2017. Austria. 44 (2 Supplement 1) (pp S566), 2017. Date of Publication: 2017.
[Conference Abstract]
AN: 618910471
Publisher

Springer Berlin Heidelberg

Link to the Ovid Full Text or citation:
[Click here for full text options]

Link to the External Link Resolver:
[SFX Link]

214.

Diagnostic performance of artificial neural network for the localization of coronary artery disease.
Shimoyama H., Nakayama S., Kotake Y., Shimamoto S., Futai R.
Embase
European Journal of Nuclear Medicine and Molecular Imaging. Conference: 30th Annual Congress of the European Association of Nuclear Medicine, EANM 2017. Austria. 44 (2 Supplement 1) (pp S338), 2017. Date of Publication: 2017.
[Conference Abstract]
AN: 618910225
Publisher
Springer Berlin Heidelberg

Link to the Ovid Full Text or citation:
[Click here for full text options]

Link to the External Link Resolver:
[SFX Link]

215.

AMMO-X: A cross-comparison study of 13N-ammonia PET MPQ software tools.
Nesterov S.V., Juarez-Orozco L.E., Knol R.J., Sciagra R., Slomka P., Alessio A., Van Der Zant F., Han C., Kartiosuo N., Knuuti J.M.
Embase
European Journal of Nuclear Medicine and Molecular Imaging. Conference: 30th Annual Congress of the European Association of Nuclear Medicine, EANM 2017. Austria. 44 (2 Supplement 1) (pp S299-S300), 2017. Date of Publication: 2017.
[Conference Abstract]
AN: 618910205
Publisher
Springer Berlin Heidelberg

Link to the Ovid Full Text or citation:
[Click here for full text options]

Link to the External Link Resolver:
[SFX Link]

216.

Artificial intelligence for myocardial perfusion imaging compared with expert interpretation.
Nakajima K., Kiso K., Kudo T., Taniguchi Y., Matsuo S., Nakagawa M., Nakata T., Hida S., Tanaka H., Sarai M., Yokoyama K., Momose M., Okuda K., Edenbrandt L.
Embase
European Journal of Nuclear Medicine and Molecular Imaging. Conference: 30th Annual Congress of the European Association of Nuclear Medicine, EANM 2017. Austria. 44 (2 Supplement 1) (pp S337-S338), 2017. Date of Publication: 2017.
[Conference Abstract]
AN: 618910172
Publisher
Springer Berlin Heidelberg

Link to the Ovid Full Text or citation:
Click here for full text options

Link to the External Link Resolver:
SFX Link

217.

Prognostic value of quantitative absolute myocardial stress perfusion in patients with chest pain and normal coronary arteries: A Nitrogen-13 Ammonia PET study.
Monroy-Gonzalez A.G., Tio R.A., Alexanderson-Rosas E., Slart R.H.J.A.
Embase
European Journal of Nuclear Medicine and Molecular Imaging. Conference: 30th Annual Congress of the European Association of Nuclear Medicine, EANM 2017. Austria. 44 (2 Supplement 1) (pp S300-S301), 2017. Date of Publication: 2017.
[Conference Abstract]
AN: 618908707
Publisher
Springer Berlin Heidelberg

Link to the Ovid Full Text or citation:
Click here for full text options

Link to the External Link Resolver:
SFX Link

218.

Quantitative myocardial stress perfusion in patients with chest pain and normal coronary arteries to assess subsequent improvement of symptoms with transcutaneous electrical nerve stimulation.
Monroy-Gonzalez A.G., De Jongste M.J.L., Alexanderson-Rosas E., Tio R.A., Slart

R.H.J.A.
Embase
European Journal of Nuclear Medicine and Molecular Imaging. Conference: 30th Annual Congress of the European Association of Nuclear Medicine, EANM 2017. Austria. 44 (2 Supplement 1) (pp S236), 2017. Date of Publication: 2017.
[Conference Abstract]
AN: 618908695
Publisher
Springer Berlin Heidelberg

Link to the Ovid Full Text or citation:
Click here for full text options

Link to the External Link Resolver:
SFX Link

219.

Possibility of software program provided artificial neural network (ANN) analysis supporting interpretation of medical staffs in myocardial perfusion SPECT.
Koyama K., Yamada H., Ogura T., Kanou M., Maehara K., Ino T., Hoshizaki H., Oshima S., Toyama T.
Embase
European Journal of Nuclear Medicine and Molecular Imaging. Conference: 30th Annual Congress of the European Association of Nuclear Medicine, EANM 2017. Austria. 44 (2 Supplement 1) (pp S571-S572), 2017. Date of Publication: 2017.
[Conference Abstract]
AN: 618908557
Publisher
Springer Berlin Heidelberg

Link to the Ovid Full Text or citation:
Click here for full text options

Link to the External Link Resolver:
SFX Link

220.

Analysis of raw polar maps from myocardial perfusion SPECT by gender-adjusted deep learning improves automatic prediction of obstructive coronary disease.
Betancur J.A., Commandeur F., Sharir T., Fish M., Ruddy T., Kaufmann P., Bateman T., Dorbala S., Germano G., Berman D., Dey D., Slomka P.
Embase
Journal of Nuclear Cardiology. Conference: 22nd Annual Scientific Session of the American Society of Nuclear Cardiology. United States. 24 (4) (pp 1492-1493), 2017. Date of Publication: July-August 2017.
[Conference Abstract]
AN: 617902218
Publisher

Springer New York LLC

Link to the Ovid Full Text or citation:
Click here for full text options

Link to the External Link Resolver:
SFX Link

221.

13th National Congress of the Italian Association of Nuclear Medicine and Molecular Imaging, AIMN 2017.
Anonymous
Embase
Clinical and Translational Imaging. Conference: 13th National Congress of the Italian Association of Nuclear Medicine and Molecular Imaging, AIMN 2017. Italy. 5 (Supplement 1) (no pagination), 2017. Date of Publication: April 2017.
[Conference Review]
AN: 617718691
Publisher
Springer-Verlag Italia s.r.l.

Link to the Ovid Full Text or citation:
Click here for full text options

Link to the External Link Resolver:
SFX Link

222.

Impact of non-specific normal databases on perfusion quantification of low-dose myocardial SPECT studies.
Scabbio C., Lecchi M., Del Sole A., Lucignani G.
Embase
Clinical and Translational Imaging. Conference: 13th National Congress of the Italian Association of Nuclear Medicine and Molecular Imaging, AIMN 2017. Italy. 5 (Supplement 1) (pp S138), 2017. Date of Publication: April 2017.
[Conference Abstract]
AN: 617718349
Publisher
Springer-Verlag Italia s.r.l.

Link to the Ovid Full Text or citation:
Click here for full text options

Link to the External Link Resolver:
SFX Link

223.

Improving clinical decisions in PAH management: Simulation in continuing education.

Spyropoulos J., Hanley K., Warters M., Blevins D.
Embase
American Journal of Respiratory and Critical Care Medicine. Conference: American Thoracic Society International Conference, ATS 2017. United States. 195 (no pagination), 2017. Date of Publication: 2017.
[Conference Abstract]
AN: 617707656
Publisher
American Thoracic Society

Link to the Ovid Full Text or citation:
Click here for full text options

Link to the External Link Resolver:
SFX Link

224.

Rest scan does not improve automatic machine learning prediction of major adverse coronary events after high speed myocardial perfusion imaging.
Betancur J.A., Otaki Y., Fish M., Lemley M., Dey D., Tamarappoo B., Germano G., Berman D., Slomka P.
Embase
Journal of the American College of Cardiology. Conference: 66th Annual Scientific Session of the American College of Cardiology and i2 Summit: Innovation in Intervention, ACC.17. United States. 69 (11 Supplement 1) (pp 1590), 2017. Date of Publication: 21 Mar 2017.
[Conference Abstract]
AN: 617219977
Publisher
Elsevier USA

Link to the Ovid Full Text or citation:
Click here for full text options

Link to the External Link Resolver:
SFX Link

225.

North American Society of Cardiovascular Imaging, NASCI AHA Young Investigator Finalists 2016.
Anonymous

Embase
International Journal of Cardiovascular Imaging. Conference: 2016 Annual Meeting of the North American Society of Cardiovascular Imaging, NASCI 2016. United States. 33 (3) (no pagination), 2017. Date of Publication: March 2017.
[Conference Review]
AN: 614835701
Publisher
Springer Netherlands

Link to the Ovid Full Text or citation:
Click here for full text options

Link to the External Link Resolver:
SFX Link

226.

ATLS Hypovolemic Shock Classification by Prediction of Blood Loss in Rats Using Regression Models.
Choi S.B., Choi J.Y., Park J.S., Kim D.W.
Embase
Shock. 46 (1) (pp 92-98), 2016. Date of Publication: 01 Jul 2016.
[Article]
AN: 607997727
Publisher
Lippincott Williams and Wilkins (E-mail: kathiest.clai@apta.org)

Link to the Ovid Full Text or citation:
Click here for full text options

Link to the External Link Resolver:
SFX Link

227.

Cardiovascular imaging 2015 in the International Journal of Cardiovascular Imaging.
Bezerra H.G., Costa R.A., Reiber J.H.C., Schoenhagen P., Stillman A.A., De Sutter J., Van de Veire N.R.L.
Embase
International Journal of Cardiovascular Imaging. 32 (5) (pp 697-709), 2016. Date of Publication: 01 May 2016.
[Editorial]
AN: 609979034
Publisher
Springer Netherlands

Link to the Ovid Full Text or citation:
Click here for full text options

Link to the External Link Resolver:
SFX Link

228.

Automated Quantitative Nuclear Cardiology Methods.
Motwani M., Berman D.S., Germano G., Slomka P.
Embase
Cardiology Clinics. 34 (1) (pp 47-57), 2016. Date of Publication: February 2016.
[Review]
AN: 606989786
Publisher
W.B. Saunders

Link to the Ovid Full Text or citation:
Click here for full text options

Link to the External Link Resolver:
SFX Link

229.

Letter by Baumann et al regarding article, "Fractional flow reserve and coronary computed tomographic angiography: A review and critical analysis".
Baumann S., Tesche C., Schoepf U.J., Akin I., Borggrefe M., Renker M.
Embase
Circulation Research. 119 (6) (pp e106-e107), 2016. Date of Publication: 02 Sep 2016.
[Letter]
AN: 612018152
Publisher
Lippincott Williams and Wilkins (E-mail: kathiest.clai@apta.org)

Link to the Ovid Full Text or citation:
Click here for full text options

Link to the External Link Resolver:
SFX Link

230.

EComment. SPECT perfusion quantification for chronic total occlusion.
Hudorovic N., Visnja V.-H.
Embase
Interactive Cardiovascular and Thoracic Surgery. 23 (1) (pp 149), 2016. Date of Publication: 01 Jul 2016.
[Note]

AN: 611321399
Publisher
Oxford University Press (E-mail: jnl.info@oup.co.uk)

Link to the Ovid Full Text or citation:
Click here for full text options

Link to the External Link Resolver:
SFX Link

231.

Robust semi-automated quantification of cardiac MR perfusion using level set: Application to hypertrophic cardiomyopathy patient data.
Kim Y.-C., Kim S.M., Choe Y.H.
Embase
Computers in Biology and Medicine. 71 (pp 162-173), 2016. Date of Publication: April 01, 2016.
[Article]
AN: 608753410
Publisher
Elsevier Ltd

Link to the Ovid Full Text or citation:
Click here for full text options

Link to the External Link Resolver:
SFX Link

232.

Artificial neural network modeling enhances risk stratification and can reduce downstream testing for patients with suspected acute coronary syndromes, negative cardiac biomarkers, and normal ECGs.
Isma'eel H.A., Cremer P.C., Khalaf S., Almedawar M.M., Elhajj I.H., Sakr G.E., Jaber W.A.
Embase
International Journal of Cardiovascular Imaging. 32 (4) (pp 687-696), 2016. Date of Publication: 01 Apr 2016.
[Article]
AN: 607222592
Publisher
Springer Netherlands

Link to the Ovid Full Text or citation:
Click here for full text options

Link to the External Link Resolver:
SFX Link

233.

Normal Databases for the Relative Quantification of Myocardial Perfusion.
Rubeaux M., Xu Y., Germano G., Berman D.S., Slomka P.J.
Embase
Current Cardiovascular Imaging Reports. 9 (8) (no pagination), 2016. Article Number: 22. Date of Publication: 01 Aug 2016.
[Review]
AN: 611041678
Publisher
Current Medicine Group LLC 1 (E-mail: info@phl.cursci.com)

Link to the Ovid Full Text or citation:
Click here for full text options

Link to the External Link Resolver:
SFX Link

234.

Perfusion quantification of vascular malformations using contrast-enhanced ultrasound (CEUS) with time intensity curve analysis before and after treatment: First results.
Wiesinger I., Schreml S., Wohlgemuth W.A., Stroszczynski C., Jung E.M.
Embase
Clinical Hemorheology and Microcirculation. 62 (4) (pp 283-290), 2016. Date of Publication: 13 May 2016.
[Article]
AN: 610580806
Publisher
IOS Press (Nieuwe Hemweg 6B, Amsterdam 1013 BG, Netherlands)

Link to the Ovid Full Text or citation:
Click here for full text options

Link to the External Link Resolver:
SFX Link

235.

Quantitative myocardial perfusion with dynamic contrast-enhanced imaging in MRI and CT: Theoretical models and current implementation.
Pelgrim G.J., Handayani A., Dijkstra H., Prakken N.H.J., Slart R.H.J.A., Oudkerk M., Van Ooijen P.M.A., Vliegenthart R., Sijens P.E.
Embase
BioMed Research International. 2016 (no pagination), 2016. Article Number:

1734190. Date of Publication: 2016.
[Review]
AN: 609230918
Publisher
Hindawi Publishing Corporation (410 Park Avenue, 15th Floor, 287 pmb, New York NY 10022, United States)

Link to the Ovid Full Text or citation:
Click here for full text options

Link to the External Link Resolver:
SFX Link

236.

The optimum hematocrit.
Reinhart W.H.
Embase
Clinical Hemorheology and Microcirculation. 64 (4) (pp 575-585), 2016. Date of Publication: 2016.
[Article]
AN: 614321534
Publisher
IOS Press (Nieuwe Hemweg 6B, Amsterdam 1013 BG, Netherlands)

Link to the Ovid Full Text or citation:
Click here for full text options

Link to the External Link Resolver:
SFX Link

237.

Automatic valve plane localization in solidstate myocardial perfusion spect images by machine learning: Anatomical and diagnostic validation.
Betancur J.A., Rubeaux M., Fuchs T., Otaki Y., Germano G., Berman D., Kaufmann P., Slomka P.
Embase
Journal of Nuclear Cardiology. Conference: 21st Annual Scientific Session of the American Society of Nuclear Cardiolog. United States. 23 (4) (pp 920-921), 2016. Date of Publication: August 2016.
[Conference Abstract]
AN: 611620826
Publisher
Springer New York LLC

Link to the Ovid Full Text or citation:
Click here for full text options

Link to the External Link Resolver:
[SFX Link]

238.

A simple and effective model for prediction of cardiac death after adenosine myocardial perfusion spect based on machine learning.
Haro Alonso D., Wernick M., Yang Y., Germano G., Berman D.S., Slomka P.
Embase
Journal of Nuclear Cardiology. Conference: 21st Annual Scientific Session of the American Society of Nuclear Cardiolog. United States. 23 (4) (pp 915-916), 2016.
Date of Publication: August 2016.
[Conference Abstract]
AN: 611620811
Publisher
Springer New York LLC

Link to the Ovid Full Text or citation:
[Click here for full text options]

Link to the External Link Resolver:
[SFX Link]

239.

Machine learning optimally predicts major adverse cardiac events after high-speed myocardial perfusion SPECT.
Otaki Y., Betancur J., Fish M.B., Lemley M., Dey D., Germano G., Berman D.S., Slomka P.J.
Embase
Journal of Nuclear Cardiology. Conference: 21st Annual Scientific Session of the American Society of Nuclear Cardiolog. United States. 23 (4) (pp 924-925), 2016.
Date of Publication: August 2016.
[Conference Abstract]
AN: 611620756
Publisher
Springer New York LLC

Link to the Ovid Full Text or citation:
[Click here for full text options]

Link to the External Link Resolver:
[SFX Link]

240.

Resting myocardial computed tomography perfusion provides incremental value

beyond coronary stenosis and atherosclerotic plaque characteristics for predicting lesion ischemia: A machine learning approach.
Lee J.H., Xiong G., Han D., Rizvi A., Gransar H., Elmore K., Hartaigh B.O., Lin F.Y., Min J.K.
Embase
Circulation. Conference: American Heart Association's 2016 Scientific Sessions and Resuscitation Science Symposium. United States. 134 (Supplement 1) (no pagination), 2016. Date of Publication: November 2016.
[Conference Abstract]
AN: 619220835
Publisher
Lippincott Williams and Wilkins

Link to the Ovid Full Text or citation:
Click here for full text options

Link to the External Link Resolver:
SFX Link

241.

Neuronal network analysis of myocardial texture in resting native or contrast-enhanced echocardiographic images predicts left ventricular recovery one year after myocardial infarction.
Kasprzak J.D., Skonieczka S., Simiera M., Michalski B.W., Szymczyk E., Wejner-Mik P., Lipiec P., Wierzbowska-Drabik K., Strzelecki M.
Embase
European Heart Journal. Conference: European Society of Cardiology, ESC Congress 2016. Italy. 37 (Supplement 1) (pp 655), 2016. Date of Publication: August 2016.
[Conference Abstract]
AN: 612282844
Publisher
Oxford University Press

Link to the Ovid Full Text or citation:
Click here for full text options

Link to the External Link Resolver:
SFX Link

242.

Predicting hemodynamics from metabolic measurements using support vector regression in an isolated ex-vivo fourchamber working heart model.
Gaasedelen E.N., Seewald M.S., Illes T.L., Mattison L.M., Mattson A.R., Schmidt M.M., Iaizzo P.A.
Embase
European Heart Journal: Acute Cardiovascular Care. Conference: Acute Cardiovascular Care 2016. Portugal. 5 (Supplement 1) (pp 378-379), 2016. Date of

Publication: October 2016.
[Conference Abstract]
AN: 613191927
Publisher
SAGE Publications Inc.

Link to the Ovid Full Text or citation:
Click here for full text options

Link to the External Link Resolver:
SFX Link

243.

Relationship between Tc-99m MIBI GATED SPECT myocardial perfusion, quantified functional analysis, and dyssynchrony assessment in patients with left bundle branch block (LBBB) and no evidence of Ischemic heart disease-An approach to the real facts.
Villoria G.A., RodadoMarina S., Catalan P., Castro-Beiras J., Lorente B., Ramirez Y., Dominguez Gadea L., Gomez M.
Embase
European Journal of Nuclear Medicine and Molecular Imaging. Conference: 29th Annual Congress of the European Association of Nuclear Medicine, EANM 2016. Spain. 43 (1 Supplement 1) (pp S546-S547), 2016. Date of Publication: 2016.
[Conference Abstract]
AN: 613165101
Publisher
Springer Berlin

Link to the Ovid Full Text or citation:
Click here for full text options

Link to the External Link Resolver:
SFX Link

244.

SPECT myocardial perfusion imaging quantification in obese subjects: Influence of adipose tissue in the attenuation correction with computed tomography attenuation maps.
Melo I., Stakhiv O., Jessop M., Begley P., Dizdarevic S., Sousa E.
Embase
European Journal of Nuclear Medicine and Molecular Imaging. Conference: 29th Annual Congress of the European Association of Nuclear Medicine, EANM 2016. Spain. 43 (1 Supplement 1) (pp S143-S144), 2016. Date of Publication: 2016.
[Conference Abstract]
AN: 613165100
Publisher
Springer Berlin

Link to the Ovid Full Text or citation:
Click here for full text options

Link to the External Link Resolver:
SFX Link

245.

Simulation to solve the artifacts and pitfalls myocardial SPECT using data mining.
Asakura K., Kazuhiro S., Miho K., Akihito O.
Embase
European Journal of Nuclear Medicine and Molecular Imaging. Conference: 29th Annual Congress of the European Association of Nuclear Medicine, EANM 2016. Spain. 43 (1 Supplement 1) (pp S661), 2016. Date of Publication: 2016.
[Conference Abstract]
AN: 613164446
Publisher
Springer Berlin

Link to the Ovid Full Text or citation:
Click here for full text options

Link to the External Link Resolver:
SFX Link

246.

Impact of non-specific normal databases on perfusion quantification of low-time myocardial SPECTstudies in overweight or obese patients.
Scabbio C., Lecchi M., Del Sole A., Lucignani G.
Embase
European Journal of Nuclear Medicine and Molecular Imaging. Conference: 29th Annual Congress of the European Association of Nuclear Medicine, EANM 2016. Spain. 43 (1 Supplement 1) (pp S504), 2016. Date of Publication: 2016.
[Conference Abstract]
AN: 613164242
Publisher
Springer Berlin

Link to the Ovid Full Text or citation:
Click here for full text options

Link to the External Link Resolver:
SFX Link

247.

Impact of cardiac motion on myocardial blood flow (MBF) using Dynamic SPECT-CZT imaging in patients with coronary artery disease.
Nganoa C., Maguet M., Manrique A., Roule V., Plane A., Peyronnet D., Baavour R., Roth N., Beygui F., Agostini D.
Embase
European Journal of Nuclear Medicine and Molecular Imaging. Conference: 29th Annual Congress of the European Association of Nuclear Medicine, EANM 2016. Spain. 43 (1 Supplement 1) (pp S557-S558), 2016. Date of Publication: 2016.
[Conference Abstract]
AN: 613164202
Publisher
Springer Berlin

Link to the Ovid Full Text or citation:
Click here for full text options

Link to the External Link Resolver:
SFX Link

248.

Assessment of Myocardial Flow Reserve (MFR) by Dynamic SPECT using CZT camera and Comparison with invasive Fractional Flow Reserve (FFR) Measurements in Patients with Stable Angina. Preliminary Results of Waterday Study.
Agostini D., Roule V., Nganoa C., Vigne J., Salomon T., Wain-Hobson J., Maguet M., Baavour R., Roth N., Beygui F., Manrique A.
Embase
European Journal of Nuclear Medicine and Molecular Imaging. Conference: 29th Annual Congress of the European Association of Nuclear Medicine, EANM 2016. Spain. 43 (1 Supplement 1) (pp S168), 2016. Date of Publication: 2016.
[Conference Abstract]
AN: 613163681
Publisher
Springer Berlin

Link to the Ovid Full Text or citation:
Click here for full text options

Link to the External Link Resolver:
SFX Link

249.

First validation of myocardial flow reserve (MFR) by Dynamic SPECT acquisitions using CZT camera in Comparison with O15-water PET in patients with stable angina. Preliminary results of waterday study.
Agostini D., Roule V., Nganoa C., Wain-Hobson J., Peyronnet D., Maguet M., Baavour R., Roth N., Beygui F., Manrique A.
Embase

European Journal of Nuclear Medicine and Molecular Imaging. Conference: 29th Annual Congress of the European Association of Nuclear Medicine, EANM 2016. Spain. 43 (1 Supplement 1) (pp S168), 2016. Date of Publication: 2016.
[Conference Abstract]
AN: 613163651
Publisher
Springer Berlin

Link to the Ovid Full Text or citation:
Click here for full text options

Link to the External Link Resolver:
SFX Link

250.

The impact of seed variation on the measured accuracy of machine learning algorithms (AUC) for several common model validation techniques in nuclear medicine.
Singh V.R., Slomka P.
Embase
Journal of Investigative Medicine. Conference: American Federation for Medical Research Western Regional Meeting, AFMR 2016. United States. 64 (1) (pp 236-237), 2016. Date of Publication: January 2016.
[Conference Abstract]
AN: 612648304
Publisher
Lippincott Williams and Wilkins

Link to the Ovid Full Text or citation:
Click here for full text options

Link to the External Link Resolver:
SFX Link

251.

Detection of acute myocardial ischemia using resting F18-DG PET-CT imaging.
Ghilardi A., Medolago G., Lorenza P., Brambati N., Iampietro E., Bianchi C., Bruno A.

Embase
Journal of Nuclear Medicine. Conference: Society of Nuclear Medicine and Molecular Imaging Annual Meeting, SNMMI 2016. San Diego, CA United States. Conference Publication: (var.pagings). 57 (SUPPL. 2) (no pagination), 2016. Date of Publication: May 2016.
[Conference Abstract]
AN: 72336406
Publisher
Society of Nuclear Medicine Inc.

Link to the Ovid Full Text or citation:
Click here for full text options

Link to the External Link Resolver:
SFX Link

252.

Automatic valve plane localization in myocardial perfusion SPECT images using machine learning.
Betancur J., Rubeaux M., Fuchs T., Slipczuk L., Germano G., Dey D., Berman D., Kaufmann P., Slomka P.
Embase
Journal of Nuclear Medicine. Conference: Society of Nuclear Medicine and Molecular Imaging Annual Meeting, SNMMI 2016. San Diego, CA United States. Conference Publication: (var.pagings). 57 (SUPPL. 2) (no pagination), 2016. Date of Publication: May 2016.
[Conference Abstract]
AN: 72334955
Publisher
Society of Nuclear Medicine Inc.

Link to the Ovid Full Text or citation:
Click here for full text options

Link to the External Link Resolver:
SFX Link

253.

Computer-assisted cardiopulmonary bypass: The possible way to improve safety.
Semenova A., Agapov V., Shigaev M.
Embase
Journal of Cardiothoracic and Vascular Anesthesia. Conference: 31st Annual Meeting of the European Association of Cardiothoracic Anaesthesiologists, EACTA 2016. Basel Switzerland. Conference Publication: (var.pagings). 30 (SUPPL. 1) (pp S10), 2016. Date of Publication: May 2016.
[Conference Abstract]
AN: 72329015
Publisher
W.B. Saunders

Link to the Ovid Full Text or citation:
Click here for full text options

Link to the External Link Resolver:
SFX Link

254.

Incremental benefit of computed tomography derived resting myocardial perfusion using machine learning method and computed tomography derived fractional flow reserve for predicting lesion ischemia.
Lee J.H., Rizvi A., Han D., Hartaigh B.O., Gransar H., Lin F., Min J., Xiong G.
Embase
Journal of the American College of Cardiology. Conference: 65th Annual Scientific Session of the American College of Cardiology and i2 Summit: Innovation in Intervention, ACC.16. Chicago, IL United States. Conference Publication: (var.pagings). 67 (13 SUPPL. 1) (pp 1677), 2016. Date of Publication: 05 Apr 2016.
[Conference Abstract]
AN: 72243180
Publisher
Elsevier USA

Link to the Ovid Full Text or citation:
Click here for full text options

Link to the External Link Resolver:
SFX Link

255.

Incremental role of resting myocardial computed tomography perfusion for predicting physiologically significant coronary artery disease: A machine learning method approach.
Han D., Lee J.H., Rizvi A., Elmore K., Gransar H., Hartaigh B.O., Lin F., Min J., Xiong G.
Embase
Journal of the American College of Cardiology. Conference: 65th Annual Scientific Session of the American College of Cardiology and i2 Summit: Innovation in Intervention, ACC.16. Chicago, IL United States. Conference Publication: (var.pagings). 67 (13 SUPPL. 1) (pp 1671), 2016. Date of Publication: 05 Apr 2016.
[Conference Abstract]
AN: 72243174
Publisher
Elsevier USA

Link to the Ovid Full Text or citation:
Click here for full text options

Link to the External Link Resolver:
SFX Link

256.

Predicting acute ischemic stroke tissue fate using deep learning on source perfusion MRI.
Ho K.C., El-Saden S., Scalzo F., Bui A.A., Arnold C.W.
Embase
Stroke. Conference: American Heart Association/American Stroke Association 2016 International Stroke Conference and State-of-the-Science Stroke Nursing Symposium. Los Angeles, CA United States. Conference Publication: (var.pagings). 47 (SUPPL. 1) (no pagination), 2016. Date of Publication: February 2016.
[Conference Abstract]
AN: 72211264
Publisher
Lippincott Williams and Wilkins

Link to the Ovid Full Text or citation:
[Click here for full text options]

Link to the External Link Resolver:
[SFX Link]

257.

Reducing variability in dual bolus cardiac MRI by using empirical contrast ratios.
Chatterjee N., Benefield B.C., Lee D.C., Carroll T.
Embase
Journal of Cardiovascular Magnetic Resonance. Conference: 19th Annual SCMR Scientific Sessions. Los Angeles, CA United States. Conference Publication: (var.pagings). 18 (SUPPL. 1) (no pagination), 2016. Date of Publication: 27 Jan 2016.
[Conference Abstract]
AN: 72183362
Publisher
BioMed Central Ltd.

Link to the Ovid Full Text or citation:
[Click here for full text options]

Link to the External Link Resolver:
[SFX Link]

258.

Correlations and validations of dual-bolus and dual-sequence quantification of first-pass myocardial perfusion CMR in humans and canines.
Hsu L.-Y., Kellman P., Gatehouse P., Conn H.M., Benovoy M., Jacobs M., Arai A.E.
Embase
Journal of Cardiovascular Magnetic Resonance. Conference: 19th Annual SCMR Scientific Sessions. Los Angeles, CA United States. Conference Publication: (var.pagings). 18 (SUPPL. 1) (no pagination), 2016. Date of Publication: 27 Jan 2016.
[Conference Abstract]

AN: 72183258
Publisher
BioMed Central Ltd.

Link to the Ovid Full Text or citation:
Click here for full text options

Link to the External Link Resolver:
SFX Link

259.

Quantitative myocardial perfusion imaging using a step arterial-input function.
Thompson R.B., Grenier J., Sonnex E., Coulden R.
Embase
Journal of Cardiovascular Magnetic Resonance. Conference: 19th Annual SCMR Scientific Sessions. Los Angeles, CA United States. Conference Publication: (var.pagings). 18 (SUPPL. 1) (no pagination), 2016. Date of Publication: 27 Jan 2016.
[Conference Abstract]
AN: 72182756
Publisher
BioMed Central Ltd.

Link to the Ovid Full Text or citation:
Click here for full text options

Link to the External Link Resolver:
SFX Link

260.

Myocardial perfusion analysis in cardiac computed tomography angiographic images at rest.
Xiong G., Kola D., Heo R., Elmore K., Cho I., Min J.K.
Embase
Medical Image Analysis. 24 (1) (pp 77-89), 2015. Date of Publication: May 01, 2015.
[Article]
AN: 604789296
Publisher
Elsevier B.V.

Link to the Ovid Full Text or citation:
Click here for full text options

Link to the External Link Resolver:
SFX Link

261.

Percutaneous intramyocardial delivery of mesenchymal stem cells induces superior improvement in regional left ventricular function compared with bone marrow mononuclear cells in porcine myocardial infarcted heart.
Tao B., Cui M., Wang C., Ma S., Wu F., Yi F., Qin X., Liu J., Wang H., Wang Z., Ma X., Tian J., Chen Y., Wang J., Cao F.
Embase
Theranostics. 5 (2) (pp 196-205), 2015. Date of Publication: 2015.
[Article]
AN: 602683962
Publisher
Ivyspring International Publisher (E-mail: info@ivyspring.com)

Link to the Ovid Full Text or citation:
Click here for full text options

Link to the External Link Resolver:
SFX Link

262.

Influence of spatial resolution on the accuracy of quantitative myocardial perfusion in first pass stress perfusion CMR.
Zarinabad N., Chiribiri A., Hautvast G.L.T.F., Breeuwer M., Nagel E.
Embase
Magnetic Resonance in Medicine. 73 (4) (pp 1623-1631), 2015. Date of Publication: 01 Apr 2015.
[Article]
AN: 53146420
Publisher
John Wiley and Sons Inc. (P.O.Box 18667, Newark NJ 07191-8667, United States)

Link to the Ovid Full Text or citation:
Click here for full text options

Link to the External Link Resolver:
SFX Link

263.

Quantifying Plaque Burden and Morphology Using Coronary Computed Tomography Angiography to Predict Coronary Physiology: Helpful.But Is It Sufficient?.
Di Carli M.F., Blankstein R.
Embase
Circulation: Cardiovascular Imaging. 8 (10) (no pagination), 2015. Article Number: e004058. Date of Publication: 01 Oct 2015.

[Editorial]
AN: 606699548
Publisher
Lippincott Williams and Wilkins (E-mail: kathiest.clai@apta.org)

Link to the Ovid Full Text or citation:
Click here for full text options

Link to the External Link Resolver:
SFX Link

264.

Tracer kinetic modeling in myocardial perfusion quantification using MRI.
Schwab F., Ingrisch M., Marcus R., Bamberg F., Hildebrandt K., Adrion C., Gliemi C., Nikolaou K., Reiser M., Theisen D.
Embase
Magnetic Resonance in Medicine. 73 (3) (pp 1206-1215), 2015. Date of Publication: 01 Mar 2015.
[Article]
AN: 53084681
Publisher
John Wiley and Sons Inc. (P.O.Box 18667, Newark NJ 07191-8667, United States)

Link to the Ovid Full Text or citation:
Click here for full text options

Link to the External Link Resolver:
SFX Link

265.

Myocardial perfusion SPECT: Perfusion quantification.
Kakhki V.R.D.
Embase
Iranian Journal of Nuclear Medicine. 23 (1) (pp 49-52), 2015. Date of Publication: 2015.
[Review]
AN: 601010935
Publisher
Tehran University of Medical Sciences (E-mail: afotouhi@tums.ac.ir afotouhi@tums.ac.ir)

Link to the Ovid Full Text or citation:
Click here for full text options

Link to the External Link Resolver:
SFX Link

266.

Computerized decision making in myocardial perfusion SPECT: The new era in nuclear cardiology?.
Tragardh E., Carlsson M., Edenbrandt L.
Embase
Journal of Nuclear Cardiology. 22 (5) (pp 885-887), 2015. Date of Publication: 11 Dec 2014.
[Editorial]
AN: 600766746
Publisher
Springer New York LLC (E-mail: barbara.b.bertram@gsk.com)

Link to the Ovid Full Text or citation:
Click here for full text options

Link to the External Link Resolver:
SFX Link

267.

A quantitative high resolution voxel-wise assessment of myocardial blood flow from contrast-enhanced first-pass magnetic resonance perfusion imaging: microsphere validation in a magnetic resonance compatible free beating explanted pig heart model.
Schuster A., Sinclair M., Zarinabad N., Ishida M., Van Den Wijngaard J.P.H.M., Paul M., Van Horssen P., Hussain S.T., Perera D., Schaeffter T., Spaan J.A.E., Siebes M., Nagel E., Chiribiri A.
Embase
European Heart Journal Cardiovascular Imaging. 16 (10) (pp 1082-1092), 2015. Date of Publication: 2015.
[Article]
AN: 607709734
Publisher
Oxford University Press (E-mail: jnl.info@oup.co.uk)

Link to the Ovid Full Text or citation:
Click here for full text options

Link to the External Link Resolver:
SFX Link

268.

Diagnostics of significant coronary stenoses in patients with myocardial perfusion disorders by the data of monofoton emission computed tomography of myocardium using mathematic instrument of artificial neuronal networks.

Kuznetsov V.A., Yaroslavskaya E.I., Krinochkin D.V., Teffenberg D.V., Kutrunov V.N., Diachkov S.M.
Embase
Russian Journal of Cardiology. 128 (12) (pp 14-19), 2015. Date of Publication: 01 Jan 2015.
[Article]
AN: 608718058
Publisher
Silicea-Poligraf

Link to the Ovid Full Text or citation:
Click here for full text options

Link to the External Link Resolver:
SFX Link

269.

Spectrotemporal CT data acquisition and reconstruction at low dose.
Clark D.P., Lee C.-L., Kirsch D.G., Badea C.T.
Embase
Medical Physics. 42 (11) (pp 6317-6336), 2015. Date of Publication: 01 Nov 2015.
[Article]
AN: 606327353
Publisher
AAPM - American Association of Physicists in Medicine

Link to the Ovid Full Text or citation:
Click here for full text options

Link to the External Link Resolver:
SFX Link

270.

Prediction of revascularization after myocardial perfusion SPECT by machine learning in a large population.
Arsanjani R., Dey D., Khachatryan T., Shalev A., Hayes S.W., Fish M., Nakanishi R., Germano G., Berman D.S., Slomka P.
Embase
Journal of Nuclear Cardiology. 22 (5) (pp 877-884), 2015. Date of Publication: 06 Dec 2014.
[Article]
AN: 600751245
Publisher
Springer New York LLC (E-mail: journals@springer-sbm.com)

Link to the Ovid Full Text or citation:
Click here for full text options

Link to the External Link Resolver:
SFX Link

271.

Diagnostic Performance of Artificial Neural Network for Detecting Ischemia in Myocardial Perfusion Imaging.
Nakajima K., Matsuo S., Wakabayashi H., Yokoyama K., Bunko H., Okuda K., Kinuya S., Nystrom K., Edenbrandt L.
Embase
Circulation journal : official journal of the Japanese Circulation Society. 79 (7) (pp 1549-1556), 2015. Date of Publication: 2015.
[Article]
AN: 609965654

Link to the Ovid Full Text or citation:
Click here for full text options

Link to the External Link Resolver:
SFX Link

272.

Influence of attenuation correction in the MPI image reconstructed by the evolution for cardiacTM software.
Ribeiro D.F.F., Vilaca J., Nunes C., Freire L., Pinheiro M., Cantinho G., Carolino E., Sousa E.
Embase
European Journal of Nuclear Medicine and Molecular Imaging. Conference: 28th Annual Congress of the European Association of Nuclear Medicine, EANM 2015. Hamburg Germany. Conference Publication: (var.pagings). 42 (1 SUPPL. 1) (pp S812), 2015. Date of Publication: October 2015.
[Conference Abstract]
AN: 72076166
Publisher
springer berlin

Link to the Ovid Full Text or citation:
Click here for full text options

Link to the External Link Resolver:
SFX Link

273.

SPECT myocardial perfusion imaging quantification in obese subjects: Influence of

attenuation correction with computed tomography attenuation maps.
Stakhiv O., Clarke M., Aplin M., Singh N., Day K., Dizdarevic S., Jessop M., Sousa E.

Embase
European Journal of Nuclear Medicine and Molecular Imaging. Conference: 28th Annual Congress of the European Association of Nuclear Medicine, EANM 2015. Hamburg Germany. Conference Publication: (var.pagings). 42 (1 SUPPL. 1) (pp S167-S168), 2015. Date of Publication: October 2015.
[Conference Abstract]
AN: 72074873
Publisher
springer berlin

Link to the Ovid Full Text or citation:
Click here for full text options

Link to the External Link Resolver:
SFX Link

274.

Detection of myocardial ischemia using artificial neural network: Different characteristics from conventional defect scoring methods.
Nakajima K., Matsuo S., Okuda K., Yokoyama K., Bunko H., Kinuya S., Edenbrandt L.
Embase
European Journal of Nuclear Medicine and Molecular Imaging. Conference: 28th Annual Congress of the European Association of Nuclear Medicine, EANM 2015. Hamburg Germany. Conference Publication: (var.pagings). 42 (1 SUPPL. 1) (pp S73-S74), 2015. Date of Publication: October 2015.
[Conference Abstract]
AN: 72074704
Publisher
springer berlin

Link to the Ovid Full Text or citation:
Click here for full text options

Link to the External Link Resolver:
SFX Link

275.

AQUA-5: Four software tools for 15O-Water PET myocardial perfusion quantification exhibit good intraobserver reproducibility.
Nesterov S.V., Yoshinaga K., Tomiyama Y., Han C., Sciagra R., Berti V., Knappen P., Danad I., Knuuti J.M.
Embase
European Journal of Nuclear Medicine and Molecular Imaging. Conference: 28th Annual Congress of the European Association of Nuclear Medicine, EANM 2015.

Hamburg Germany. Conference Publication: (var.pagings). 42 (1 SUPPL. 1) (pp S55), 2015. Date of Publication: October 2015.
[Conference Abstract]
AN: 72074668
Publisher
springer berlin

Link to the Ovid Full Text or citation:
Click here for full text options

Link to the External Link Resolver:
SFX Link

276.

Three-dimensional placental perfusion imaging using velocity-selective arterial spin labeled MRI: Preliminary results.
Zun Z., Shankaranarayanan A., Bulas D., Du Plessis A.J., Limperopoulos C.
Embase
Prenatal Diagnosis. Conference: 19th International Conference on Prenatal Diagnosis and Theraphy, ISPD 2015. Washington, DC United States. Conference Publication: (var.pagings). 35 (SUPPL. 1) (pp 3-4), 2015. Date of Publication: July 2015.
[Conference Abstract]
AN: 72072180
Publisher
John Wiley and Sons Ltd

Link to the Ovid Full Text or citation:
Click here for full text options

Link to the External Link Resolver:
SFX Link

277.

Global myocardial perfusion quantified in mild to severe systemic sclerosis; Novel insights from MR stress imaging of coronary sinus flow.
Gyllenhammar T., Kanski M., Engblom H., Wuttge D., Carlsson M., Hesselstrand R., Arheden H.
Embase
European Heart Journal. Conference: European Society of Cardiology, ESC Congress 2015. London United Kingdom. Conference Publication: (var.pagings). 36 (SUPPL. 1) (pp 307), 2015. Date of Publication: 01 Aug 2015.
[Conference Abstract]
AN: 72019921
Publisher
Oxford University Press

Link to the Ovid Full Text or citation:
Click here for full text options

Link to the External Link Resolver:
SFX Link

278.

Artificial neural network modelling enhances risk stratification for patients with suspected acute coronary syndromes, negative cardiac biomarkers, and normal ECGs.
Cremer P., Ismaeel H., Sakr G., Elhajj I., Khalaf S., Jaber W.
Embase
European Heart Journal. Conference: European Society of Cardiology, ESC Congress 2015. London United Kingdom. Conference Publication: (var.pagings). 36 (SUPPL. 1) (pp 79), 2015. Date of Publication: 01 Aug 2015.
[Conference Abstract]
AN: 72019084
Publisher
Oxford University Press

Link to the Ovid Full Text or citation:
Click here for full text options

Link to the External Link Resolver:
SFX Link

279.

Automated perfusion computer axial tomography predicts acute stroke deficits.
Rorden C., Fridriksson J., Thors H., Hillis A., Krebs K., Fridriksson J.F., Graham C.B., Hubbard I., Hanayik T., Sen S.
Embase
Stroke. Conference: American Heart Association/American Stroke Association 2015 International Stroke Conference and State-of-the-Science Stroke Nursing Symposium. Nashville, TN United States. Conference Publication: (var.pagings). 46 (SUPPL. 1) (no pagination), 2015. Date of Publication: February 2015.
[Conference Abstract]
AN: 71819007
Publisher
Lippincott Williams and Wilkins

Link to the Ovid Full Text or citation:
Click here for full text options

Link to the External Link Resolver:
SFX Link

280.

Automation and quantification of the angiographic capillary blush in patients with acute ischemic stroke undergoing endovascular intervention.
Tarpley J., Scalzo F., Alger J.R., Aghaebrahim A., Liang C., Sheth S., Noorian R., Ng K., Jovin T., Duckwiler G., Liebeskind D.S.
Embase
Stroke. Conference: American Heart Association/American Stroke Association 2015 International Stroke Conference and State-of-the-Science Stroke Nursing Symposium. Nashville, TN United States. Conference Publication: (var.pagings). 46 (SUPPL. 1) (no pagination), 2015. Date of Publication: February 2015.
[Conference Abstract]
AN: 71818489
Publisher
Lippincott Williams and Wilkins

Link to the Ovid Full Text or citation:
Click here for full text options

Link to the External Link Resolver:
SFX Link

281.

Cardiac magnetic resonance dual bolus myocardial perfusion quantification superior to the single bolus analysis method.
Goetschalckx K., Claus P., Bogaert J., Toth A., Merkely B., Janssens S., Rademakers F.E.
Embase
Journal of Cardiovascular Magnetic Resonance. Conference: 18th Annual SCMR Scientific Sessions. Nice France. Conference Publication: (var.pagings). 17 (SUPPL. 1) (no pagination), 2015. Date of Publication: 03 Feb 2015.
[Conference Abstract]
AN: 71794927
Publisher
BioMed Central Ltd.

Link to the Ovid Full Text or citation:
Click here for full text options

Link to the External Link Resolver:
SFX Link

282.

Shared versus non-shared prepulse perfusion MR sequence in absolute myocardial perfusion quantification.
Goetschalckx K., Rademakers F.E., Bogaert J., Toth A., Merkely B., Janssens S., Claus P.

Embase
Journal of Cardiovascular Magnetic Resonance. Conference: 18th Annual SCMR Scientific Sessions. Nice France. Conference Publication: (var.pagings). 17 (SUPPL. 1) (no pagination), 2015. Date of Publication: 03 Feb 2015.
[Conference Abstract]
AN: 71794912
Publisher
BioMed Central Ltd.

Link to the Ovid Full Text or citation:
Click here for full text options

Link to the External Link Resolver:
SFX Link

283.

Myocardial Blood Flow Quantification from MRI - an Image Analysis Perspective.
Zarinabad N., Chiribiri A., Breeuwer M.
Embase
Current Cardiovascular Imaging Reports. 7 (1) (pp 1-9), 2014. Article Number: 9246. Date of Publication: 2014.
[Review]
AN: 372245939
Publisher
Current Medicine Group LLC 1

Link to the Ovid Full Text or citation:
Click here for full text options

Link to the External Link Resolver:
SFX Link

284.

Free breathing myocardial perfusion data sets for performance analysis of motion compensation algorithms.
Wollny G., Kellman P.
Embase
GigaScience. 3 (1) (no pagination), 2014. Article Number: 23. Date of Publication: November 11, 2014.
[Article]
AN: 603871329
Publisher
BioMed Central Ltd. (E-mail: jnl.info@oup.co.uk)

Link to the Ovid Full Text or citation:
Click here for full text options

Link to the External Link Resolver:
SFX Link

285.

Effects of tracer arrival time on the accuracy of high-resolution (Voxel-Wise) myocardial perfusion maps from contrast-enhanced first-pass perfusion magnetic resonance.
Zarinabad N., Hautvast G.L.T.F., Sammut E., Arujuna A., Breeuwer M., Nagel E., Chiribiri A.
Embase
IEEE Transactions on Biomedical Engineering. 61 (9) (pp 2499-2506), 2014. Article Number: 6813677. Date of Publication: September 2014.
[Article]
AN: 373823372
Publisher
IEEE Computer Society (E-mail: help@computer.org)

Link to the Ovid Full Text or citation:
Click here for full text options

Link to the External Link Resolver:
SFX Link

286.

Gleaning knowledge from data in the intensive care unit.
Pinsky M.R., Dubrawski A.
Embase
American Journal of Respiratory and Critical Care Medicine. 190 (6) (pp 606-610), 2014. Date of Publication: 15 Sep 2014.
[Review]
AN: 604384330
Publisher
American Thoracic Society (E-mail: malexander@thoracic.org)

Link to the Ovid Full Text or citation:
Click here for full text options

Link to the External Link Resolver:
SFX Link

287.

Clinical decision support systems in myocardial perfusion imaging.
Garcia E.V., Klein J.L., Taylor A.T.
Embase

Journal of Nuclear Cardiology. 21 (3) (pp 427-439), 2014. Date of Publication: June 2014.
[Review]
AN: 52985177
Publisher
Springer New York LLC (E-mail: journals@springer-sbm.com)

Link to the Ovid Full Text or citation:
Click here for full text options

Link to the External Link Resolver:
SFX Link

288.

Myocardial perfusion quantification using the T1-based FAIR-ASL method: The influence of heart anatomy, cardiopulmonary blood flow and look-locker readout.
Kampf T., Helluy X., Gutjahr F.T., Winter P., Meyer C.B., Jakob P.M., Bauer W.R., Ziener C.H.
Embase
Magnetic Resonance in Medicine. 71 (5) (pp 1784-1797), 2014. Date of Publication: May 2014.
[Article]
AN: 52675044
Publisher
John Wiley and Sons Inc. (P.O.Box 18667, Newark NJ 07191-8667, United States)

Link to the Ovid Full Text or citation:
Click here for full text options

Link to the External Link Resolver:
SFX Link

289.

An eog-based vigilance estimation method applied for driver fatigue detection.
Ma J.-X., Shi L.-C., Lu B.-L.
Embase
Neuroscience and Biomedical Engineering. 2 (1) (pp 41-51), 2014. Date of Publication: 01 Jan 2014.
[Article]
AN: 603565729
Publisher
Bentham Science Publishers B.V. (P.O. Box 294, Bussum 1400 AG, Netherlands)

Link to the Ovid Full Text or citation:
Click here for full text options

Link to the External Link Resolver:

SFX Link

290.

A spatially-distributed computational model to quantify behaviour of contrast agents in MR perfusion imaging.
Cookson A.N., Lee J., Michler C., Chabiniok R., Hyde E., Nordsletten D., Smith N.P.
Embase
Medical Image Analysis. 18 (7) (pp 1200-1216), 2014. Date of Publication: October 2014.
[Article]
AN: 373688618

Link to the Ovid Full Text or citation:
Click here for full text options

Link to the External Link Resolver:
SFX Link

291.

Numerical surrogates for human observers in myocardial motion evaluation from SPECT images.
Marin T., Kalayeh M.M., Parages F.M., Brankov J.G.
Embase
IEEE Transactions on Medical Imaging. 33 (1) (pp 38-47), 2014. Article Number: 6584807. Date of Publication: January 2014.
[Article]
AN: 372114765
Publisher
Institute of Electrical and Electronics Engineers Inc. (445 Hoes Lane / P.O. Box 1331, Piscataway NJ 08855-1331, United States)

Link to the Ovid Full Text or citation:
Click here for full text options

Link to the External Link Resolver:
SFX Link

292.

Prognostic value of blood flow measurements using arterial spin labeling in gliomas.
Furtner J., Katsaros V.K., Bender B., Braun C.B., Schittenhelm J., Skardelly M., Bisdas S.
Embase
Neuroradiology. Conference: 20th Symposium Neuroradiologicum 2014. Istanbul Turkey. Conference Publication: (var.pagings). 56 (SUPPL. 1) (pp 294-295), 2014.

Date of Publication: September 2014.
[Conference Abstract]
AN: 71812804
Publisher
Springer Verlag

Link to the Ovid Full Text or citation:
[Click here for full text options]

Link to the External Link Resolver:
[SFX Link]

293.

Prediction of contractile reserve based on neuronal network analysis of myocardial texture in resting native and contrast-enhanced echocardiographic images.
Kasprzak J.D., Michalski B., Skonieczka S., Szymczyk E., Wejner-Mik P., Lipiec P., Strzelecki M.
Embase
European Heart Journal. Conference: European Society of Cardiology, ESC Congress 2014. Barcelona Spain. Conference Publication: (var.pagings). 35 (SUPPL. 1) (pp 283), 2014. Date of Publication: 01 Sep 2014.
[Conference Abstract]
AN: 71647791
Publisher
Oxford University Press

Link to the Ovid Full Text or citation:
[Click here for full text options]

Link to the External Link Resolver:
[SFX Link]

294.

Efficacy of terutroban in preventing delayed cerebral ischemia after subarachnoid haemorrhage: A functional isotope imaging study on a rat model.
Lagier D., Velly L., Bruder N., Alessi M.C., Guillet B.
Embase
European Journal of Anaesthesiology. Conference: European Anaesthesiology Congress, EUROANAESTHESIA 2014. Stockholm Sweden. Conference Publication: (var.pagings). 31 (SUPPL. 52) (pp 109), 2014. Date of Publication: June 2014.
[Conference Abstract]
AN: 71638160
Publisher
Lippincott Williams and Wilkins

Link to the Ovid Full Text or citation:
[Click here for full text options]

Link to the External Link Resolver:
SFX Link

295.

Comparison between automated perfusion quantification of 99MTC myocardial perfusion spect and late gadolinium enhancement of cardiac MRI.
Suzuki Y., Matsumoto N., Masuoka K., Ashida T., Yoda S., Nagao K., Hirayama A.
Embase
Journal of Nuclear Cardiology. Conference: 19th Annual Scientific Session of the American Society of Nuclear Cardiology, ASNC 2014. Boston, MA United States. Conference Publication: (var.pagings). 21 (4) (pp 773), 2014. Date of Publication: August 2014.
[Conference Abstract]
AN: 71620873
Publisher
Springer New York

Link to the Ovid Full Text or citation:
Click here for full text options

Link to the External Link Resolver:
SFX Link

296.

Efficacy of terutroban in preventing delayed cerebral ischemia after subarachnoid hemorrhage: A functional isotope imaging study on a rat model.
Lagier D., Guillet B., Velly L., Bruder N., Alessi M.
Embase
Critical Care. Conference: 34th International Symposium on Intensive Care and Emergency Medicine. Brussels Belgium. Conference Publication: (var.pagings). 18 (SUPPL. 1) (pp S166), 2014. Date of Publication: 17 Mar 2014.
[Conference Abstract]
AN: 71507175
Publisher
BioMed Central Ltd.

Link to the Ovid Full Text or citation:
Click here for full text options

Link to the External Link Resolver:
SFX Link

297.

Relationship between Pixel-wise Quantification of Regadenoson Stress CMR Endocardial to Epicardial Myocardial Perfusion Ratios versus Quantitative Coronary Angiography.
Winkler S., Allison T., Conn H., Bandettini P., Shanbhag S., Kellman P., Hsu L.Y., Arai A.E.
Embase
European Heart Journal Cardiovascular Imaging. Conference: 12th Annual Meeting of the Cardiovascular Magnetic Resonance Section of the European Association of Cardiovascular Imaging, EuroCMR 2014. Vienna Austria. Conference Publication: (var.pagings). 15 (SUPPL. 1) (pp i4), 2014. Date of Publication: May 2014.
[Conference Abstract]
AN: 71506616
Publisher
Oxford University Press

Link to the Ovid Full Text or citation:
[Click here for full text options]

Link to the External Link Resolver:
[SFX Link]

298.

Improved accuracy of myocardial perfusion spect for prediction of revascularization by machine learning in a large population.
Arsanjani R., Dey D., Shalev A., Khachatryan T., Hayes S., Fish M., Berman D., Germano G., Slomka P.
Embase
Journal of the American College of Cardiology. Conference: 63rd Annual Scientific Session of the American College of Cardiology and i2 Summit: Innovation in Intervention, ACC.14. Washington, DC United States. Conference Publication: (var.pagings). 63 (12 SUPPL. 1) (pp A1229), 2014. Date of Publication: 01 Apr 2014.
[Conference Abstract]
AN: 71407253
Publisher
Elsevier USA

Link to the Ovid Full Text or citation:
[Click here for full text options]

Link to the External Link Resolver:
[SFX Link]

299.

Impact of errors in determining the start of myocardial enhancement during quantitative first pass perfusion cardiovascular magnetic resonance imaging.
Ta A.D., Hsu L.-Y., Miller C.A., Conn H., Winkler S., Kellman P., Nguyen K.-L., Shanbhag S.M.
Embase

Journal of Cardiovascular Magnetic Resonance. Conference: 17th Annual SCMR Scientific Sessions. New Orleans, LA United States. Conference Publication: (var.pagings). 16 (SUPPL. 1) (no pagination), 2014. Date of Publication: 16 Jan 2014.
[Conference Abstract]
AN: 71330516
Publisher
BioMed Central Ltd.

Link to the Ovid Full Text or citation:
Click here for full text options

Link to the External Link Resolver:
SFX Link

300.

Quantification of perfusion defects with high resolution 2D and 3D adenosine stress perfusion 3.0 Tesla CMR.
McDiarmid A.K., Motwani M., Ripley D.P., Mohee K., Kozerke S., Greenwood J.P., Plein S.
Embase
Journal of Cardiovascular Magnetic Resonance. Conference: 17th Annual SCMR Scientific Sessions. New Orleans, LA United States. Conference Publication: (var.pagings). 16 (SUPPL. 1) (no pagination), 2014. Date of Publication: 16 Jan 2014.
[Conference Abstract]
AN: 71330349
Publisher
BioMed Central Ltd.

Link to the Ovid Full Text or citation:
Click here for full text options

Link to the External Link Resolver:
SFX Link

301.

Registration of coronary MRA to DCE-MRI myocardial perfusion series improves diagnostic accuracy through the computation of patientspecific coronary supply territories: A CE-MARC sub-study.
Zakkaroff C., Radjenovic A., Biglands J.D., Plein S., Greenwood J.P., Magee D.R.
Embase
Journal of Cardiovascular Magnetic Resonance. Conference: 17th Annual SCMR Scientific Sessions. New Orleans, LA United States. Conference Publication: (var.pagings). 16 (SUPPL. 1) (no pagination), 2014. Date of Publication: 16 Jan 2014.
[Conference Abstract]
AN: 71330068

Publisher
BioMed Central Ltd.

Link to the Ovid Full Text or citation:
[Click here for full text options]

Link to the External Link Resolver:
[SFX Link]

302.

Automatic model-based contour detection of left ventricle myocardium from cardiac CT images.
Sugiura T., Takeguchi T., Sakata Y., Nitta S., Okazaki T., Matsumoto N., Fujisawa Y.

Embase
International Journal of Computer Assisted Radiology and Surgery. 8 (1) (pp 145-155), 2013. Date of Publication: January 2013.
[Article]
AN: 51997421
Publisher
Springer Verlag (E-mail: service@springer.de)

Link to the Ovid Full Text or citation:
[Click here for full text options]

Link to the External Link Resolver:
[SFX Link]

303.

Improved accuracy of myocardial perfusion SPECT for the detection of coronary artery disease using a support vector machine algorithm.
Arsanjani R., Xu Y., Dey D., Fish M., Dorbala S., Hayes S., Berman D., Germano G., Slomka P.
Embase
Journal of Nuclear Medicine. 54 (4) (pp 549-555), 2013. Date of Publication: 01 Apr 2013.
[Article]
AN: 368653972
Publisher
Society of Nuclear Medicine Inc. (1850 Samuel Morse Drive, Reston VA 22090-5316, United States)

Link to the Ovid Full Text or citation:
[Click here for full text options]

Link to the External Link Resolver:
[SFX Link]

304.

Computing ischemic regions in the heart with the bidomain model - First steps towards validation.
Nielsen B.F., Lysaker M., Grottum P.
Embase
IEEE Transactions on Medical Imaging. 32 (6) (pp 1085-1096), 2013. Article Number: 6484983. Date of Publication: 2013.
[Article]
AN: 369050855
Publisher
Institute of Electrical and Electronics Engineers Inc. (445 Hoes Lane / P.O. Box 1331, Piscataway NJ 08855-1331, United States)

Link to the Ovid Full Text or citation:
Click here for full text options

Link to the External Link Resolver:
SFX Link

305.

Multislice cardiac arterial spin labeling using improved myocardial perfusion quantification with simultaneously measured blood pool input function.
Campbell-Washburn A.E., Zhang H., Siow B.M., Price A.N., Lythgoe M.F., Ordidge R.J., Thomas D.L.
Embase
Magnetic Resonance in Medicine. 70 (4) (pp 1125-1136), 2013. Date of Publication: October 2013.
[Article]
AN: 52314158
Publisher
John Wiley and Sons Inc. (P.O.Box 18667, Newark NJ 07191-8667, United States)

Link to the Ovid Full Text or citation:
Click here for full text options

Link to the External Link Resolver:
SFX Link

306.

Dynamic CT Perfusion Imaging of the Myocardium: A Technical Note on Improvement of Image Quality.
Muenzel D., Kabus S., Gramer B., Leber V., Vembar M., Schmitt H., Wildgruber M., Fingerle A.A., Rummeny E.J., Huber A., Noel P.B.

Embase
PLoS ONE. 8 (10) (no pagination), 2013. Article Number: e75263. Date of Publication: 09 Oct 2013.
[Review]
AN: 369979886
Publisher
Public Library of Science (185 Berry Street, Suite 1300, San Francisco CA 94107, United States)

Link to the Ovid Full Text or citation:
Click here for full text options

Link to the External Link Resolver:
SFX Link

307.

Mortality prediction of rats in acute hemorrhagic shock using machine learning techniques.
Kim K.-A., Choi J.Y., Yoo T.K., Kim S.K., Chung K., Kim D.W.
Embase
Medical and Biological Engineering and Computing. 51 (9) (pp 1059-1067), 2013. Date of Publication: September 2013.
[Article]
AN: 52646109
Publisher
Springer Verlag (E-mail: service@springer.de)

Link to the Ovid Full Text or citation:
Click here for full text options

Link to the External Link Resolver:
SFX Link

308.

New perspectives in electronic fetal surveillance.
Hamilton E.F., Warrick P.A.
Embase
Journal of Perinatal Medicine. 41 (1) (pp 83-92), 2013. Date of Publication: January 2013.
[Review]
AN: 370372066
Publisher
Walter de Gruyter GmbH and Co. KG (Genthiner Strasse 13, Berlin D-10785, Germany)

Link to the Ovid Full Text or citation:
Click here for full text options

Link to the External Link Resolver:
SFX Link

309.

Improved accuracy of myocardial perfusion SPECT for detection of coronary artery disease by machine learning in a large population.
Arsanjani R., Xu Y., Dey D., Vahistha V., Shalev A., Nakanishi R., Hayes S., Fish M., Berman D., Germano G., Slomka P.J.
Embase
Journal of Nuclear Cardiology. 20 (4) (pp 553-562), 2013. Date of Publication: August 2013.
[Article]
AN: 52597476
Publisher
Springer New York (233 Spring Street, New York NY 10013-1578, United States)

Link to the Ovid Full Text or citation:
Click here for full text options

Link to the External Link Resolver:
SFX Link

310.

Cine-ASL: A steady-pulsed arterial spin labeling method for myocardial perfusion mapping in mice. Part II. Theoretical model and sensitivity optimization.
Capron T., Troalen T., Cozzone P.J., Bernard M., Kober F.
Embase
Magnetic Resonance in Medicine. 70 (5) (pp 1399-1408), 2013. Date of Publication: November 2013.
[Article]
AN: 52375176
Publisher
John Wiley and Sons Inc. (P.O.Box 18667, Newark NJ 07191-8667, United States)

Link to the Ovid Full Text or citation:
Click here for full text options

Link to the External Link Resolver:
SFX Link

311.

Contrast-enhanced ultrasound to evaluate changes in renal cortical perfusion around cardiac surgery: A pilot study.

Schneider A.G., Goodwin M.D., Schelleman A., Bailey M., Johnson L., Bellomo R.
Embase
Critical Care. 17 (4) (no pagination), 2013. Article Number: R138. Date of Publication: 12 Jul 2013.
[Article]
AN: 52683097
Publisher
BioMed Central Ltd. (Floor 6, 236 Gray's Inn Road, London WC1X 8HB, United Kingdom)

Link to the Ovid Full Text or citation:
Click here for full text options

Link to the External Link Resolver:
SFX Link

312.

Simultaneous dual-isotope imaging based on an artificial neural network for evaluating myocardial perfusion and fatty acid metabolism.
Hashimoto J., Ogawa K., Bai J., Kubo A., Imai Y.
Embase
Journal of Nuclear Cardiology. 20 (3) (pp 396-405), 2013. Date of Publication: June 2013.
[Article]
AN: 52471956
Publisher
Springer New York (233 Spring Street, New York NY 10013-1578, United States)

Link to the Ovid Full Text or citation:
Click here for full text options

Link to the External Link Resolver:
SFX Link

313.

Evaluation of the channelized Hotelling observer with an internal-noise model in a train-test paradigm for cardiac SPECT defect detection.
Brankov J.G.
Embase
Physics in medicine and biology. 58 (20) (pp 7159-7182), 2013. Date of Publication: 21 Oct 2013.
[Article]
AN: 603386280

Link to the Ovid Full Text or citation:
Click here for full text options

Link to the External Link Resolver:
SFX Link

314.

Measurement of plasma input functions using MRI.
Nicolay K.
Embase
Molecular Imaging and Biology. Conference: 2013 World Molecular Imaging Congress, WMIC 2013. Savannah, GA United States. Conference Publication: (var.pagings). 15 (1 SUPPL. 1) (pp S13-S14), 2013. Date of Publication: December 2013.
[Conference Abstract]
AN: 71373977
Publisher
Springer New York

Link to the Ovid Full Text or citation:
Click here for full text options

Link to the External Link Resolver:
SFX Link

315.

Project AQUA: Towards standardization of software tools for myocardial perfusion quantification with 15O-water PET.
Nesterov S.V., Harms H.J., Sciagra R., Berti V., Han C., Klein R., DeKemp R., Gwet K.L., Yoshinaga K., Katoh C., Knaapen P., Knuuti J.
Embase
European Journal of Nuclear Medicine and Molecular Imaging. Conference: 26th Annual Congress of the European Association of Nuclear Medicine, EANM 2013. Lyon France. Conference Publication: (var.pagings). 40 (SUPPL. 2) (pp S155), 2013. Date of Publication: October 2013.
[Conference Abstract]
AN: 71357494
Publisher
Springer Verlag

Link to the Ovid Full Text or citation:
Click here for full text options

Link to the External Link Resolver:
SFX Link

316.

Interobserver reproducibility of myocardial perfusion quantification with 13N-ammonia and 82Rb PET studied with Carimas.
Nesterov S.V., Deshayes E., Settimo L., Han C., Ryzhkova D.V., Kostina I.S., Maki M., Prior J.O., Sciagra R., Knuuti J.
Embase
European Journal of Nuclear Medicine and Molecular Imaging. Conference: 26th Annual Congress of the European Association of Nuclear Medicine, EANM 2013. Lyon France. Conference Publication: (var.pagings). 40 (SUPPL. 2) (pp S155), 2013. Date of Publication: October 2013.
[Conference Abstract]
AN: 71357493
Publisher
Springer Verlag

Link to the Ovid Full Text or citation:
[Click here for full text options]

Link to the External Link Resolver:
[SFX Link]

317.

3-D imaging and illustration of nerve-lesion association in the mouse tongue of experimental oral cancer.
Hua T.-E., Liu K.-J., Tang S.-C.
Embase
Cancer Research. Conference: 104th Annual Meeting of the American Association for Cancer Research, AACR 2013. Washington, DC United States. Conference Publication: (var.pagings). 73 (8 SUPPL. 1) (no pagination), 2013. Date of Publication: 15 Apr 2013.
[Conference Abstract]
AN: 71342141
Publisher
American Association for Cancer Research Inc.

Link to the Ovid Full Text or citation:
[Click here for full text options]

Link to the External Link Resolver:
[SFX Link]

318.

Classification of vasospasm after aneurysmal subarachnoid hemorrhage using datadriven machine learning techniques.
Roederer A., Holmes J.H., Lee I., Park S.
Embase
Neurocritical Care. Conference: 11th Annual Meeting of the Neurocritical Care Society. Philadelphia, PA United States. Conference Publication: (var.pagings). 19 (1 SUPPL. 1) (pp S266), 2013. Date of Publication: September 2013.

[Conference Abstract]
AN: 71194715
Publisher
Humana Press

Link to the Ovid Full Text or citation:
Click here for full text options

Link to the External Link Resolver:
SFX Link

319.

Comparative study on contrast media volume and scanning parameters of dual energy lung perfusion imaging.
Yang X., Liang X., Hou P., Liu J., Hu L.
Embase
Journal of Thoracic Imaging. Conference: 3rd World Congress of Thoracic Imaging and Diagnosis in Chest Disease, WCTI 2013. Seoul South Korea. Conference Publication: (var.pagings). 28 (5) (pp W111), 2013. Date of Publication: September 2013.
[Conference Abstract]
AN: 71181867
Publisher
Lippincott Williams and Wilkins

Link to the Ovid Full Text or citation:
Click here for full text options

Link to the External Link Resolver:
SFX Link

320.

Interpretation of ischemia in myocardial perfusion scintigraphy by two computer aided diagnosis systems.
Tragardh E., Lomsky M., Johansson L.B., Svensson S.E., Edenbrandt L.
Embase
Journal of Nuclear Cardiology. Conference: 11th International Conference of Non-Invasive Cardiovascular Imaging. Berlin Germany. Conference Publication: (var.pagings). 20 (1 SUPPL. 1) (pp S23), 2013. Date of Publication: June 2013.
[Conference Abstract]
AN: 71160050
Publisher
Springer New York

Link to the Ovid Full Text or citation:
Click here for full text options

Link to the External Link Resolver:
SFX Link

321.

Quantitative adverse plaque features from coronary CT angiography predict impaired myocardial flow reserve by 13NAmmonia-PET.
Dey D., Alexanderson E., Schuhback A., Otaki Y., Ding X., Orozco L., Meave A., Berman D.S., Achenbach S., Slomka P.
Embase
Journal of Cardiovascular Computed Tomography. Conference: 8th Annual Scientific Meeting of the Society of Cardiovascular Computed Tomography, SCCT 2013. Montreal, QC Canada. Conference Publication: (var.pagings). 7 (SUPPL. 3) (pp S58), 2013. Date of Publication: May-June 2013.
[Conference Abstract]
AN: 71148036
Publisher
Elsevier Inc.

Link to the Ovid Full Text or citation:
Click here for full text options

Link to the External Link Resolver:
SFX Link

322.

Endo-/epicardial perfusion and microvascular adaptation in a rabbit model of developing heart failure assessed with a 3D imaging cryomicrotome.
Van Lier M.G.J.T.B., Hakimzadeh N., Coronel R., Spaan J.A.E., Siebes M., Van Den Wijngaard J.P.H.M.
Embase
European Journal of Heart Failure. Conference: Heart Failure Congress 2013. Lisbon Portugal. Conference Publication: (var.pagings). 12 (SUPPL. 1) (pp S49), 2013. Date of Publication: May 2013.
[Conference Abstract]
AN: 71080016
Publisher
Oxford University Press

Link to the Ovid Full Text or citation:
Click here for full text options

Link to the External Link Resolver:
SFX Link

323.

Integrated quantitative measurement of adverse plaque features from coronary CT angiography to predict impaired myocardial flow reserve by 13N-ammonia-pet.
Dey D., Rosas E.A., Schuhbaeck A., Otaki Y., Orozco L.E.J., Nakazato R., Germano G., Meave A., Berman D., Achenbach S., Slomka P.
Embase
Journal of the American College of Cardiology. Conference: 62nd Annual Scientific Session of the American College of Cardiology and i2 Summit: Innovation in Intervention, ACC.13. San Francisco, CA United States. Conference Publication: (var.pagings). 61 (10 SUPPL. 1) (pp E1041), 2013. Date of Publication: 12 Mar 2013.

[Conference Abstract]
AN: 71020404
Publisher
Elsevier USA

Link to the Ovid Full Text or citation:
Click here for full text options

Link to the External Link Resolver:
SFX Link

324.

Improved accuracy of myocardial perfusion spect for detection of coronary artery disease by machine learning in a large population.
Arsanjani R., Xu Y., Dey D., Shalev A., Nakanishi R., Hayes S., Fish M., Berman D., Germano G., Slomka P.
Embase
Journal of the American College of Cardiology. Conference: 62nd Annual Scientific Session of the American College of Cardiology and i2 Summit: Innovation in Intervention, ACC.13. San Francisco, CA United States. Conference Publication: (var.pagings). 61 (10 SUPPL. 1) (pp E1006), 2013. Date of Publication: 12 Mar 2013.

[Conference Abstract]
AN: 71020369
Publisher
Elsevier USA

Link to the Ovid Full Text or citation:
Click here for full text options

Link to the External Link Resolver:
SFX Link

325.

Does expanded artificial intelligence improve the prognostic value of myocardial perfusion imaging? A report from the NHLBI-sponsored women's ischemia syndrome evaluation (WISE).

Doyle M., Pohost G.M., Shaw L.J., Vido D.A., Kelsey S.F., Johnson B.D., Rogers W.J., Rayarao G., Sharaf B.L., Pepine C.J., Merz N.B., Biederman R.W.
Embase
Journal of Cardiovascular Magnetic Resonance. Conference: 16th Annual SCMR Scientific Sessions. San Francisco, CA United States. Conference Publication: (var.pagings). 15 (SUPPL. 1) (pp 417), 2013. Date of Publication: 30 Jan 2013.
[Conference Abstract]
AN: 70994387
Publisher
BioMed Central Ltd.

Link to the Ovid Full Text or citation:
Click here for full text options

Link to the External Link Resolver:
SFX Link

326.

Normal myocardial perfusion values on high-resolution pixel-wise perfusion maps.
Chiribiri A., Morton G., Schuster A., Sammut E., Hautvast G., Breeuwer M., Zarinabad N., Nagel E.
Embase
Journal of Cardiovascular Magnetic Resonance. Conference: 16th Annual SCMR Scientific Sessions. San Francisco, CA United States. Conference Publication: (var.pagings). 15 (SUPPL. 1) (pp 219), 2013. Date of Publication: 30 Jan 2013.
[Conference Abstract]
AN: 70994138
Publisher
BioMed Central Ltd.

Link to the Ovid Full Text or citation:
Click here for full text options

Link to the External Link Resolver:
SFX Link

327.

The relationship between spatial resolution levels and quantitative myocardial perfusion.
Zarinabad N., Hautvast G., Breeuwer M., Nagel E., Chiribiri A.
Embase
Journal of Cardiovascular Magnetic Resonance. Conference: 16th Annual SCMR Scientific Sessions. San Francisco, CA United States. Conference Publication: (var.pagings). 15 (SUPPL. 1) (pp 177-178), 2013. Date of Publication: 30 Jan 2013.
[Conference Abstract]
AN: 70994087
Publisher
BioMed Central Ltd.

Link to the Ovid Full Text or citation:
Click here for full text options

Link to the External Link Resolver:
SFX Link

328.

Extraction of texture information using neuronal network predicts myocardial viability in native echocardiographic images.
Kasprzak J.D., Michalski B., Skonieczka S., Szymczyk E., Wejner-Mik P., Lipiec P., Strzelecki M.
Embase
European Heart Journal. Conference: European Society of Cardiology, ESC Congress 2013. Amsterdam Netherlands. Conference Publication: (var.pagings). 34 (SUPPL. 1) (pp 193), 2013. Date of Publication: August 2013.
[Conference Abstract]
AN: 71258228
Publisher
Oxford University Press

Link to the Ovid Full Text or citation:
Click here for full text options

Link to the External Link Resolver:
SFX Link

329.

Left ventricular dilation and functional impairment assessed by gated SPECT are indicators of cardiac allograft vasculopathy in heart transplant recipients.
Wenning C., Stypmann J., Papavassilis P., Sindermann J., Schober O., Hoffmeier A., Scheld H.H., Stegger L., Schafers M.
Embase
Journal of Heart and Lung Transplantation. 31 (7) (pp 719-728), 2012. Date of Publication: July 2012.
[Article]
AN: 51910830
Publisher
Elsevier USA (6277 Sea Harbor Drive, Orlando FL 32862 8239, United States)

Link to the Ovid Full Text or citation:
Click here for full text options

Link to the External Link Resolver:
SFX Link

330.

The vascular neural network - A new paradigm in stroke pathophysiology.
Zhang J.H., Badaut J., Tang J., Obenaus A., Hartman R., Pearce W.J.
Embase
Nature Reviews Neurology. 8 (12) (pp 711-716), 2012. Date of Publication: December 2012.
[Review]
AN: 52259299
Publisher
Nature Publishing Group (Houndmills, Basingstoke, Hampshire RG21 6XS, United Kingdom)

Link to the Ovid Full Text or citation:
Click here for full text options

Link to the External Link Resolver:
SFX Link

331.

Effects of hemodynamics on global and regional lung perfusion: A quantitative lung perfusion study by magnetic resonance imaging.
Cao J.J., Wang Y., McLaughlin J., Rhee P., Passick M., Ngai N., Cheng J., Gulotta R.J., Berke A.D., Petrossian G.A., Reichek N.
Embase
Circulation: Cardiovascular Imaging. 5 (6) (pp 693-699), 2012. Date of Publication: November 2012.
[Article]
AN: 368097994
Publisher
Lippincott Williams and Wilkins (530 Walnut Street,P O Box 327, Philadelphia PA 19106-3621, United States)

Link to the Ovid Full Text or citation:
Click here for full text options

Link to the External Link Resolver:
SFX Link

332.

Improved quantification in pinhole gated myocardial perfusion SPECT using micro-CT and ultrasound information.
Goethals L.R., de Geeter F., Vanhove C., Roosens B., Devos H., Lahoutte T.
Embase
Contrast Media and Molecular Imaging. 7 (2) (pp 167-174), 2012. Date of

Publication: March 2012.
[Article]
AN: 364486943
Publisher
John Wiley and Sons Ltd (Southern Gate, Chichester, West Sussex PO19 8SQ, United Kingdom)

Link to the Ovid Full Text or citation:
Click here for full text options

Link to the External Link Resolver:
SFX Link

333.

Non-pharmacological strategies in cardiovascular prevention: 2011 highlights.
Guiraud T., Labrunee M., Gayda M., Juneau M., Gremeaux V.
Embase
Annals of Physical and Rehabilitation Medicine. 55 (5) (pp 342-374), 2012. Date of Publication: July 2012.
[Short Survey]
AN: 51991532
Publisher
Elsevier Masson SAS (62 rue Camille Desmoulins, Issy les Moulineaux Cedex 92442, France)

Link to the Ovid Full Text or citation:
Click here for full text options

Link to the External Link Resolver:
SFX Link

334.

Continuous and non-invasive estimation of mean arterial blood pressure using photoplethysmograph waveform.
De Nadal M., Camps A., Ruiz-San Martin A., Garcia-Roche A., Riera J., Ruiz-Rodriguez J.C.
Embase
European Journal of Anaesthesiology. Conference: European Anaesthesiology Congress, EUROANAESTHESIA 2012. Paris France. Conference Publication: (var.pagings). 29 (SUPPL. 50) (pp 45), 2012. Date of Publication: June 2012.
[Conference Abstract]
AN: 71084135
Publisher
Lippincott Williams and Wilkins

Link to the Ovid Full Text or citation:
Click here for full text options

Link to the External Link Resolver:
SFX Link

335.

One pot preparation of 6,8 Ga-labelled macroaggregated albumin for PET-imaging of acute pulmonary embolism and myocardial perfusion quantification.
Velikyan I., Berhane T., Khan T., Borges J., Sorensen J.
Embase
European Journal of Nuclear Medicine and Molecular Imaging. Conference: 25th Annual Congress of the European Association of Nuclear Medicine, EANM 2012. Milan Italy. Conference Publication: (var.pagings). 39 (SUPPL. 2) (pp S410-S411), 2012. Date of Publication: October 2012.
[Conference Abstract]
AN: 70978006
Publisher
Springer Verlag

Link to the Ovid Full Text or citation:
Click here for full text options

Link to the External Link Resolver:
SFX Link

336.

Automated interpretation of myocardial perfusion images with Multilayer Perceptron network: A decision support system free from quantification and need for normal database.
Abbasi M., Farzanefar S., Emami A., Eftekhari M.
Embase
European Journal of Nuclear Medicine and Molecular Imaging. Conference: 25th Annual Congress of the European Association of Nuclear Medicine, EANM 2012. Milan Italy. Conference Publication: (var.pagings). 39 (SUPPL. 2) (pp S215), 2012. Date of Publication: October 2012.
[Conference Abstract]
AN: 70977267
Publisher
Springer Verlag

Link to the Ovid Full Text or citation:
Click here for full text options

Link to the External Link Resolver:
SFX Link

337.

Integrating physiologic signals with machine learning for predicting defibrillation success.
Shandilya S., Ward K., Kurz M.C., Najarian K.
Embase
Circulation. Conference: American Heart Association 2012 Scientific Sessions and Resuscitation Science Symposium. Los Angeles, CA United States. Conference Publication: (var.pagings). 126 (21 SUPPL. 1) (no pagination), 2012. Date of Publication: 20 Nov 2012.
[Conference Abstract]
AN: 70957275
Publisher
Lippincott Williams and Wilkins

Link to the Ovid Full Text or citation:
Click here for full text options

Link to the External Link Resolver:
SFX Link

338.

Automated interpretation of myocardial perfusion images with Multilayer Perceptron network: A decision support system free from quantification and need for normal database.
Abbasi M., Farzanefar S., Emami A., Eftekhari M.
Embase
Iranian Journal of Nuclear Medicine. Conference: 10th Asia-Oceania Congress of Nuclear Medicine and Biology. Tehran Iran, Islamic Republic of. Conference Publication: (var.pagings). 20 (SUPPL. 1) (pp 52), 2012. Date of Publication: May 2012.
[Conference Abstract]
AN: 70890139
Publisher
Tehran University of Medical Sciences

Link to the Ovid Full Text or citation:
Click here for full text options

Link to the External Link Resolver:
SFX Link

339.

A novel method for assessing diseased myocardium: Tracer arrival time evaluation.
Zarinabad Nooralipour N., Chiribiri N., Hautvast G., Breeuwer M., Nagel E.
Embase
European Heart Journal. Conference: ESC Congress 2012. Munchen Germany. Conference Publication: (var.pagings). 33 (SUPPL. 1) (pp 250), 2012. Date of

Publication: August 2012.
[Conference Abstract]
AN: 70883359
Publisher
Oxford University Press

Link to the Ovid Full Text or citation:
Click here for full text options

Link to the External Link Resolver:
SFX Link

340.

Improved accuracy of myocardial perfusion SPECT (MPS) for detection of coronary artery disease (CAD) by utilizing machine learning methods.
Arsanjani R., Tourassi G., Xu S., Fish M., Berman D.S., Germano G., Slomka P.J.
Embase
Journal of Nuclear Cardiology. Conference: 17th Annual Scientific Session of the American Society of Nuclear Cardiology, ASNC 2012. Baltimore, MD United States. Conference Publication: (var.pagings). 19 (4) (pp 827), 2012. Date of Publication: August 2012.
[Conference Abstract]
AN: 70850406
Publisher
Springer New York

Link to the Ovid Full Text or citation:
Click here for full text options

Link to the External Link Resolver:
SFX Link

341.

Inhibitory control task is decreased in vascular incontinence patients.
Sakakibara R., Haruta M., Ogata T., Panicker J., Fowler C., Tateno F., Yano H., Takahashi O., Yamamoto T., Yamanishi T., Uchiyama T., Yamaguchi C.
Embase
Neurourology and Urodynamics. Conference: 42nd Annual Meeting of the International Continence Society, ICS 2012. Beijing China. Conference Publication: (var.pagings). 31 (6) (pp 971-973), 2012. Date of Publication: August 2012.
[Conference Abstract]
AN: 70836073
Publisher
Wiley-Liss Inc.

Link to the Ovid Full Text or citation:
Click here for full text options

Link to the External Link Resolver:
SFX Link

342.

The blood-to-myocardium transfer coefficient, K1, determined by patlak plot analysis showed close correlation with MBF determined by dedicated perfusion CT software.
Kitagawa K., Ishida M., Ito T., Nakamori S., Nagata M., Sakuma H.
Embase
Journal of Cardiovascular Computed Tomography. Conference: 7th Annual Scietific Meeting of the Society of Cardiovascular Computed Tomography. Baltimore, MD United States. Conference Publication: (var.pagings). 6 (4 SUPPL. 1) (pp S57), 2012. Date of Publication: July-August 2012.
[Conference Abstract]
AN: 70808549
Publisher
Elsevier Inc.

Link to the Ovid Full Text or citation:
Click here for full text options

Link to the External Link Resolver:
SFX Link

343.

Steady-state first-pass perfusion (SSFPP): A 3D TWIST in myocardial first-pass perfusion imaging.
Giri S., Xue H., Wattar A., Ding Y., Kroeker R.M., Laub G., Kellman P., Zuehlsdorff S., Raman S.V., Simonetti O.P.
Embase
Journal of Cardiovascular Magnetic Resonance. Conference: 15th Annual SCMR Scientific Sessions: 2012. Orlando, FL United States. Conference Publication: (var.pagings). 14 (SUPPL. 1) (no pagination), 2012. Date of Publication: 01 Feb 2012.
[Conference Abstract]
AN: 70743695
Publisher
BioMed Central Ltd.

Link to the Ovid Full Text or citation:
Click here for full text options

Link to the External Link Resolver:
SFX Link

344.

Increased endocardial to epicardial flow ratio present at rest disappears during exercise stress perfusion CMR in normal volunteers - A potential mechanism for exercise induced subendocardial ischaemia.
Asrress K.N., Williams R., Lockie T., Biglands J.D., Chiribiri A., Radjenovic A., Jogiya R., De Silva K., Kozerke S., Chowienczyk P., Nagel E., Marber M., Redwood S., Plein S.
Embase
Journal of Cardiovascular Magnetic Resonance. Conference: 15th Annual SCMR Scientific Sessions: 2012. Orlando, FL United States. Conference Publication: (var.pagings). 14 (SUPPL. 1) (no pagination), 2012. Date of Publication: 01 Feb 2012.
[Conference Abstract]
AN: 70743670
Publisher
BioMed Central Ltd.

Link to the Ovid Full Text or citation:
Click here for full text options

Link to the External Link Resolver:
SFX Link

345.

Automated, quantitative analysis of rest myocardial perfusion obtained on 320-detector row volumetric CT can reliably identify obstructive coronary artery disease.
Qian Z., Rinehart S., Bhatt K., Odeh W., Joshi P., Dhungel A., Voros S.
Embase
Journal of the American College of Cardiology. Conference: 61th Annual Scientific Session of the American College of Cardiology and i2 Summit: Innovation in Intervention, ACC.12. Chicago, IL United States. Conference Publication: (var.pagings). 59 (13 SUPPL. 1) (pp E1360), 2012. Date of Publication: 27 Mar 2012.

[Conference Abstract]
AN: 70714800
Publisher
Elsevier USA

Link to the Ovid Full Text or citation:
Click here for full text options

Link to the External Link Resolver:
SFX Link

346.

Modern parameterization and explanation techniques in diagnostic decision support system: A case study in diagnostics of coronary artery disease.

Kukar M., Kononenko I., Groselj C.
Embase
Artificial Intelligence in Medicine. 52 (2) (pp 77-90), 2011. Date of Publication: June 2011.
[Article]
AN: 51460136
Publisher
Elsevier (P.O. Box 211, Amsterdam 1000 AE, Netherlands)

Link to the Ovid Full Text or citation:
Click here for full text options

Link to the External Link Resolver:
SFX Link

347.

Clinical data do not improve artificial neural network interpretation of myocardial perfusion scintigraphy.
Gjertsson P., Johansson L., Lomsky M., Ohlsson M., Underwood S.R., Edenbrandt L.

Embase
Clinical Physiology and Functional Imaging. 31 (3) (pp 240-245), 2011. Date of Publication: May 2011.
[Article]
AN: 51352631
Publisher
Blackwell Publishing Ltd (9600 Garsington Road, Oxford OX4 2XG, United Kingdom)

Link to the Ovid Full Text or citation:
Click here for full text options

Link to the External Link Resolver:
SFX Link

348.

Effect of content variation of Danshen water-soluble components on myocardial energy metabolism in isolated ischemia-reperfusion rat hearts.
Huang L.-H., Chen Y.-C., Cheng Y., He H., Si Q., Tang Y.-Q., Liu X.-Q.
Embase
Journal of China Pharmaceutical University. 42 (4) (pp 348-353), 2011. Date of Publication: August 2011.
[Article]
AN: 362543525
Publisher
China Pharmaceutical University (24 Tongjia Xiang, Nanjing, Jiangsu 210009, China)

Link to the Ovid Full Text or citation:

Click here for full text options

Link to the External Link Resolver:
SFX Link

349.

Comprehensive four-dimensional phase-contrast flow assessment in hemi-Fontan circulation: Systemic-to-pulmonary collateral flow quantification.
Valverde I., Rachel C., Kuehne T., Beerbaum P.
Embase
Cardiology in the Young. 21 (1) (pp 116-119), 2011. Date of Publication: February 2011.
[Article]
AN: 51127259
Publisher
Cambridge University Press (Shaftesbury Road, Cambridge CB2 2RU, United Kingdom)

Link to the Ovid Full Text or citation:
Click here for full text options

Link to the External Link Resolver:
SFX Link

350.

Validation of automated quantitation of myocardial perfusion and fatty acid metabolism abnormalities on SPECT images.
Yoshinaga K., Matsuki T., Hashimoto A., Tsukamoto K., Nakata T., Tamaki N.
Embase
Circulation Journal. 75 (9) (pp 2187-2195), 2011. Date of Publication: September 2011.
[Article]
AN: 362438735
Publisher
Japanese Circulation Society (14 Yoshida Kawaharacho, Sakyo-ku, Kyoto 606, Japan)

Link to the Ovid Full Text or citation:
Click here for full text options

Link to the External Link Resolver:
SFX Link

351.

Sinoatrial node activity is modulated by nerves emerging from the intrinsic cardiac ganglia of the pulmonary veins in the mouse heart.
Zarzoso M., Calvo C.J., Kean A.C., Pandit S., Pauza D.H., Jalife J., Noujaim. S.F.
Embase
Heart Rhythm. Conference: Cardiac Electrophysiology Society, CES Meeting 2011. Orlando, FL United States. Conference Publication: (var.pagings). 8 (11) (pp 1822), 2011. Date of Publication: November 2011.
[Conference Abstract]
AN: 71194825
Publisher
Elsevier

Link to the Ovid Full Text or citation:
[Click here for full text options]

Link to the External Link Resolver:
[SFX Link]

352.

Perfusion measurements using Arterial Spin Labeling and MRI in small animals: Comparison with radioactive and fluorescent microspheres.
Bos A., Bergmann R., Hofheinz F., Van Den Hoff J.
Embase
NuklearMedizin. Conference: 3rd International Congress of the German, Austrian and Swiss Associations of Nuclear Medicine, NuklearMedizin 2011. Bregenz Austria. Conference Publication: (var.pagings). 50 (2) (pp A10-A11), 2011. Date of Publication: 2011.
[Conference Abstract]
AN: 70975342
Publisher
Schattauer GmbH

Link to the Ovid Full Text or citation:
[Click here for full text options]

Link to the External Link Resolver:
[SFX Link]

353.

Does attenuation corrected normal stress database in myocardial perfusion scintigraphy better predict the need for a rest study and improves infarction detection?.
Tragardh Johansson E., Sjostrand K., Edenbrandt L.
Embase
European Heart Journal, Supplement. Conference: 10th International Conference of Non-Invasive Cardiovascular Imaging. Amsterdam Netherlands. Conference Publication: (var.pagings). 13 (SUPPL. A) (pp A11), 2011. Date of Publication: April 2011.

[Conference Abstract]
AN: 70777877
Publisher
Oxford University Press

Link to the Ovid Full Text or citation:
Click here for full text options

Link to the External Link Resolver:
SFX Link

354.

Half-dose/half-time WBR gated cardiac SPECT validation.
Pena H., Cantinho G., Wilk M., Srour Y., Godinho F.
Embase
European Heart Journal, Supplement. Conference: 10th International Conference of Non-Invasive Cardiovascular Imaging. Amsterdam Netherlands. Conference Publication: (var.pagings). 13 (SUPPL. A) (pp A10), 2011. Date of Publication: April 2011.
[Conference Abstract]
AN: 70777873
Publisher
Oxford University Press

Link to the Ovid Full Text or citation:
Click here for full text options

Link to the External Link Resolver:
SFX Link

355.

Computer aided diagnosis systems for the interpretation of myocardial perfusion scintigrams.
Johansson L., Lomsky M., Marving J., Svensson S., Edenbrandt L.
Embase
European Journal of Nuclear Medicine and Molecular Imaging. Conference: Annual Congress of the European Association of Nuclear Medicine 2011. Birmingham United Kingdom. Conference Publication: (var.pagings). 38 (SUPPL. 2) (pp S307), 2011. Date of Publication: October 2011.
[Conference Abstract]
AN: 70580696
Publisher
Springer Verlag

Link to the Ovid Full Text or citation:
Click here for full text options

Link to the External Link Resolver:
SFX Link

356.

Bayesian and artificial neural networks algorithms at myocardial perfusion imaging.
Vieira D., Nogueira F., Metello L.F.
Embase
European Journal of Nuclear Medicine and Molecular Imaging. Conference: Annual Congress of the European Association of Nuclear Medicine 2011. Birmingham United Kingdom. Conference Publication: (var.pagings). 38 (SUPPL. 2) (pp S270-S271), 2011. Date of Publication: October 2011.
[Conference Abstract]
AN: 70580543
Publisher
Springer Verlag

Link to the Ovid Full Text or citation:
Click here for full text options

Link to the External Link Resolver:
SFX Link

357.

Gated-SPECT myocardial perfusion imaging: Early 5 vs 30 minutes post-stress acquisition, using a high resolution matrix [128 x 128 pxls] and iterative reconstruction.
Ghilardi A., Medolago G.
Embase
European Journal of Nuclear Medicine and Molecular Imaging. Conference: Annual Congress of the European Association of Nuclear Medicine 2011. Birmingham United Kingdom. Conference Publication: (var.pagings). 38 (SUPPL. 2) (pp S136-S137), 2011. Date of Publication: October 2011.
[Conference Abstract]
AN: 70580042
Publisher
Springer Verlag

Link to the Ovid Full Text or citation:
Click here for full text options

Link to the External Link Resolver:
SFX Link

358.

Attenuation corrected images in myocardial perfusion scintigraphy better predicts the need for a rest study than non-corrected images.
Tragardh-Johansson E., Nystrom K., Edenbrandt L.
Embase
European Journal of Nuclear Medicine and Molecular Imaging. Conference: Annual Congress of the European Association of Nuclear Medicine 2011. Birmingham United Kingdom. Conference Publication: (var.pagings). 38 (SUPPL. 2) (pp S104), 2011. Date of Publication: October 2011.
[Conference Abstract]
AN: 70579924
Publisher
Springer Verlag

Link to the Ovid Full Text or citation:
Click here for full text options

Link to the External Link Resolver:
SFX Link

359.

Automated quantification of myocardial perfusion based on segmentation and non-rigid registration of contrast-enhanced cardiac magnetic resonance images.
Tarroni G., Corsi C., Veronesi F., Walter J., Lamberti C., Lang R.M., Mor-Avi V., Patel A.R.
Embase
Journal of Cardiovascular Magnetic Resonance. Conference: 2011 SCMR/Euro CMR Joint Scientific Sessions. Nice France. Conference Publication: (var.pagings). 13 (SUPPL. 1) (no pagination), 2011. Date of Publication: 2011.
[Conference Abstract]
AN: 70465609
Publisher
BioMed Central Ltd.

Link to the Ovid Full Text or citation:
Click here for full text options

Link to the External Link Resolver:
SFX Link

360.

Dynamic simulation of first pass myocardial perfusion MR with a novel perfusion phantom.
Chiribiri A., Schuster A., Ishida M., Hautvast G., Nooralipour N.Z., Paul M., Hussain S., Batchelor P., Breeuwer M., Schaeffter T., Nagel E.
Embase
Journal of Cardiovascular Magnetic Resonance. Conference: 2011 SCMR/Euro CMR Joint Scientific Sessions. Nice France. Conference Publication: (var.pagings). 13 (SUPPL. 1) (no pagination), 2011. Date of Publication: 2011.

[Conference Abstract]
AN: 70465450
Publisher
BioMed Central Ltd.

Link to the Ovid Full Text or citation:
Click here for full text options

Link to the External Link Resolver:
SFX Link

361.

PET assessment of myocardial perfusion reserve inversely correlates with intravascular ultrasound findings in angiographically normal cardiac transplant recipients.
Wu Y.-W., Chen Y.-H., Wang S.-S., Jui H.-Y., Yen R.-F., Tzen K.-Y., Chen M.-F., Lee C.-M.
Embase
Journal of Nuclear Medicine. 51 (6) (pp 906-912), 2010. Date of Publication: June 2010.
[Article]
AN: 359685742
Publisher
Society of Nuclear Medicine Inc. (1850 Samuel Morse Drive, Reston VA 22090-5316, United States)

Link to the Ovid Full Text or citation:
Click here for full text options

Link to the External Link Resolver:
SFX Link

362.

Head to head comparison of quantitative versus visual analysis of contrast CMR in the setting of myocardial stunning after STEMI: Implications on late systolic function and patient outcome.
Husser O., Bodi V., Sanchis J., Nunez J., Mainar L., Merlos P., Lopez-Lereu M.P., Monmeneu J.V., Chaustre F., Rumiz E., Riegger G.A.J., Chorro F.J., Llacer A.
Embase
International Journal of Cardiovascular Imaging. 26 (5) (pp 559-569), 2010. Date of Publication: June 2010.
[Article]
AN: 50816060
Publisher
Springer Netherlands (Van Godewijckstraat 30, Dordrecht 3311 GZ, Netherlands)

Link to the Ovid Full Text or citation:

Click here for full text options

Link to the External Link Resolver:
SFX Link

363.

Quantification of myocardial perfusion by cardiovascular magnetic resonance.
Jerosch-Herold M.
Embase
Journal of Cardiovascular Magnetic Resonance. 12 (1) (no pagination), 2010. Article Number: 57. Date of Publication: 2010.
[Article]
AN: 363005961
Publisher
BioMed Central Ltd. (Floor 6, 236 Gray's Inn Road, London WC1X 8HB, United Kingdom)

Link to the Ovid Full Text or citation:
Click here for full text options

Link to the External Link Resolver:
SFX Link

364.

Quantitative myocardial perfusion PET combined with coronary anatomy derived from CT angiography: Validation of a new fusion and visualisation software.
Fricke H., Elsner A., Weise R., Bolte M., Hoff J.v.d., Burchert W., Domik G., Fricke E.

Embase
Zeitschrift fur Medizinische Physik. 19 (3) (pp 182-188), 2009. Date of Publication: August 2009.
[Article]
AN: 50546863
Publisher
Elsevier GmbH

Link to the Ovid Full Text or citation:
Click here for full text options

Link to the External Link Resolver:
SFX Link

365.

Quantitative detection of myocardial ischaemia by stress echocardiography; A

comparison with SPECT.
Gudmundsson P., Shahgaldi K., Winter R., Dencker M., Kitlinski M., Thorsson O., Willenheimer R.B., Ljunggren L.
Embase
Cardiovascular Ultrasound. 7 (1) (no pagination), 2009. Article Number: 28. Date of Publication: 2009.
[Article]
AN: 355065823
Publisher
BioMed Central Ltd. (Floor 6, 236 Gray's Inn Road, London WC1X 8HB, United Kingdom)

Link to the Ovid Full Text or citation:
Click here for full text options

Link to the External Link Resolver:
SFX Link

366.

Pattern recognition of abnormal left ventricle wall motion in cardiac MR.
Lu Y., Radau P., Connelly K., Dick A., Wright G.
Embase
Medical image computing and computer-assisted intervention : MICCAI ... International Conference on Medical Image Computing and Computer-Assisted Intervention. 12 (Pt 2) (pp 750-758), 2009. Date of Publication: 2009.
[Article]
AN: 358940347

Link to the Ovid Full Text or citation:
Click here for full text options

Link to the External Link Resolver:
SFX Link

367.

Nonrigid registration of myocardial perfusion MRI using pseudo ground truth.
Li C., Sun Y.
Embase
Medical image computing and computer-assisted intervention : MICCAI ... International Conference on Medical Image Computing and Computer-Assisted Intervention. 12 (Pt 1) (pp 165-172), 2009. Date of Publication: 2009.
[Article]
AN: 358940152

Link to the Ovid Full Text or citation:
Click here for full text options

Link to the External Link Resolver:
SFX Link

368.

An open-source framework of neural networks for diagnosis of coronary artery disease from myocardial perfusion SPECT.
Guner L.A., Karabacak N.I., Akdemir O.U., Karagoz P.S., Kocaman S.A., Cengel A., Unlu M.
Embase
Journal of Nuclear Cardiology. 17 (3) (pp 405-413), 2010. Date of Publication: June 2010.
[Article]
AN: 50824145
Publisher
Springer New York (233 Spring Street, New York NY 10013-1578, United States)

Link to the Ovid Full Text or citation:
Click here for full text options

Link to the External Link Resolver:
SFX Link

369.

Automatic postprocessing for the assessment of quantitative human myocardial perfusion using MRI.
Weng A.M., Ritter C.O., Lotz J., Beer M.J., Hahn D., Kostler H.
Embase
European Radiology. 20 (6) (pp 1356-1365), 2010. Date of Publication: 2010.
[Article]
AN: 50738565
Publisher
Springer Verlag (Tiergartenstrasse 17, Heidelberg D-69121, Germany)

Link to the Ovid Full Text or citation:
Click here for full text options

Link to the External Link Resolver:
SFX Link

370.

Parametric quantification of myocardial ischaemia using real-time perfusion adenosine stress echocardiography images, with SPECT as reference method.
Gudmundsson P., Shahgaldi K., Winter R., Dencker M., Kitlinski M., Thorsson O., Ljunggren L., Willenheimer R.

Embase
Clinical Physiology and Functional Imaging. 30 (1) (pp 30-42), 2010. Date of Publication: January 2010.
[Article]
AN: 355835236
Publisher
Blackwell Publishing Ltd (9600 Garsington Road, Oxford OX4 2XG, United Kingdom)

Link to the Ovid Full Text or citation:
[Click here for full text options]

Link to the External Link Resolver:
[SFX Link]

371.

An automatic tool to align serial gated myocardial perfusion SPECT studies for left ventricular dyssynchrony and function measurements.
Faber T., Lin X., Santana C., Folks R., Garcia E., Soman P., Chen J.
Embase
Journal of Nuclear Medicine. Conference: Society of Nuclear Medicine and Molecular Imaging Annual Meeting, SNMMI 2010. Salt Lake City, UT United States. Conference Publication: (var.pagings). 51 (SUPPL. 2) (no pagination), 2010. Date of Publication: April-May 2010.
[Conference Abstract]
AN: 72157389
Publisher
Society of Nuclear Medicine Inc.

Link to the Ovid Full Text or citation:
[Click here for full text options]

Link to the External Link Resolver:
[SFX Link]

372.

Machine learning algorithms at myocardial perfusion imaging: A preliminary study.
Vieira D., Silva E., MacHado M., Cunha L., Metello L.F.
Embase
European Journal of Nuclear Medicine and Molecular Imaging. Conference: 23rd Annual Congress of the European Association of Nuclear Medicine, EANM 2010. Vienna Austria. Conference Publication: (var.pagings). 37 (SUPPL. 2) (pp S502-S503), 2010. Date of Publication: October 2010.
[Conference Abstract]
AN: 70977050
Publisher
Springer Verlag

Link to the Ovid Full Text or citation:
Click here for full text options

Link to the External Link Resolver:
SFX Link

373.

How to improve Gated-SPECT myocardial perfusion imaging using a high resolution matrix [128x128 pxls] and scatter correction: Preliminary results.
Ghilardi A., Medolago G., Bianchi C., Poli G.
Embase
European Journal of Nuclear Medicine and Molecular Imaging. Conference: 23rd Annual Congress of the European Association of Nuclear Medicine, EANM 2010. Vienna Austria. Conference Publication: (var.pagings). 37 (SUPPL. 2) (pp S482), 2010. Date of Publication: October 2010.
[Conference Abstract]
AN: 70976943
Publisher
Springer Verlag

Link to the Ovid Full Text or citation:
Click here for full text options

Link to the External Link Resolver:
SFX Link

374.

Automated quantification of myocardial perfusion scintigrams: A comparison of three software packages.
Lomsky M., Johansson L., Marving J., Svensson S., Edenbrandt L.
Embase
European Journal of Nuclear Medicine and Molecular Imaging. Conference: 23rd Annual Congress of the European Association of Nuclear Medicine, EANM 2010. Vienna Austria. Conference Publication: (var.pagings). 37 (SUPPL. 2) (pp S248-S249), 2010. Date of Publication: October 2010.
[Conference Abstract]
AN: 70975811
Publisher
Springer Verlag

Link to the Ovid Full Text or citation:
Click here for full text options

Link to the External Link Resolver:
SFX Link

375.

XPRESS3 - Half-dose/half-time GATED cardiac SPECT validation.
Pena H., Cantinho G., Pereira L., Magno I., Graca A., Wilk M., Srour Y., Godinho F.
Embase
European Journal of Nuclear Medicine and Molecular Imaging. Conference: 23rd Annual Congress of the European Association of Nuclear Medicine, EANM 2010. Vienna Austria. Conference Publication: (var.pagings). 37 (SUPPL. 2) (pp S201-S202), 2010. Date of Publication: October 2010.
[Conference Abstract]
AN: 70975604
Publisher
Springer Verlag

Link to the Ovid Full Text or citation:
Click here for full text options

Link to the External Link Resolver:
SFX Link

376.

XPRESS3 - Half-dose/half-time GATED cardiac SPECT validation.
Cantinho G., Pena H., Pereira L., Magno I., Godinho F.
Embase
Molecular Imaging and Biology. Conference: 2010 World Molecular Imaging Congress, WMIC 2010. Kyoto Japan. Conference Publication: (var.pagings). 12 (SUPPL. 2) (pp S1399), 2010. Date of Publication: December 2010.
[Conference Abstract]
AN: 70320312
Publisher
Springer New York

Link to the Ovid Full Text or citation:
Click here for full text options

Link to the External Link Resolver:
SFX Link

377.

Head to head comparison of quantitative versus visual analysis of contrast CMR in the setting of myocardial stunning after STEMI.
Husser O., Bodi Peris V., Sanchis J., Nunez J., Rumiz E., Lopez-Lereu M.P., Monmeneu J.V., Chaustre F., Chorro F.J., Llacer A.
Embase
European Heart Journal. Conference: European Society of Cardiology, ESC Congress 2010. Stockholm Sweden. Conference Publication: (var.pagings). 31 (SUPPL. 1) (pp 585), 2010. Date of Publication: September 2010.

[Conference Abstract]
AN: 70281767
Publisher
Oxford University Press

Link to the Ovid Full Text or citation:
[Click here for full text options]

Link to the External Link Resolver:
[SFX Link]

378.

Quantification of myocardial perfusion reserve using dynamic SPECT imaging in humans.
Breault C., Roth N., Slomka P.J., Moore S.C., Park M., Sitek A., Baavour R., Zilberstien Y., Berman D.S., Dorbala S., Hachamovitch R., Di Carli M.
Embase
Journal of Nuclear Cardiology. Conference: 15th Annual Scientific Session of the American Society of Nuclear Cardiology, ASNC2010. Philadelphia, PA United States. Conference Publication: (var.pagings). 17 (4) (pp 733-734), 2010. Date of Publication: July-August 2010.
[Conference Abstract]
AN: 70270782
Publisher
Springer New York

Link to the Ovid Full Text or citation:
[Click here for full text options]

Link to the External Link Resolver:
[SFX Link]

379.

Second generation fusion of myocardial perfusion distributions with coronary artery data from CT Coronary Angiography (CTCA).
Faber T.L., Arepalli C., Nye J.A., Folks R.D., Santana C.A., Votaw J.R., Garcia E.V., Vinten-Johansen J.
Embase
Journal of Nuclear Cardiology. Conference: 15th Annual Scientific Session of the American Society of Nuclear Cardiology, ASNC2010. Philadelphia, PA United States. Conference Publication: (var.pagings). 17 (4) (pp 722-723), 2010. Date of Publication: July-August 2010.
[Conference Abstract]
AN: 70270754
Publisher
Springer New York

Link to the Ovid Full Text or citation:
Click here for full text options

Link to the External Link Resolver:
SFX Link

380.

Preliminary classification of myocardial perfusion examinations using a short set of physiological parameters.
Cardoso G.G., Freire L.C., Almeida P.D.
Embase
European Heart Journal, Supplement. Conference: 9th International Conference of Non-Invasive Cardiovascular Imaging. Barcelona Spain. Conference Publication: (var.pagings). 11 (SUPPL. B) (pp S58), 2009. Date of Publication: May 2009.
[Conference Abstract]
AN: 70128051
Publisher
Oxford University Press

Link to the Ovid Full Text or citation:
Click here for full text options

Link to the External Link Resolver:
SFX Link

381.

Assessment of saved myocardium by primary angioplasty with myocardial perfusion imaging.
Aguade-Bruix S., Barios M., Cuberas G., Aliaga V., Sambola A., Garcia Del Blanco B., Candell-Riera J.
Embase
European Heart Journal, Supplement. Conference: 9th International Conference of Non-Invasive Cardiovascular Imaging. Barcelona Spain. Conference Publication: (var.pagings). 11 (SUPPL. B) (pp S14), 2009. Date of Publication: May 2009.
[Conference Abstract]
AN: 70127900
Publisher
Oxford University Press

Link to the Ovid Full Text or citation:
Click here for full text options

Link to the External Link Resolver:
SFX Link

382.

Need and opportunities for imaging investigation of metabolism and perfusion abnormalities in autism Spectrum Disorders and their relationship.
Shetty N., Ratai E., Herbert M.
Embase
Journal of Cerebral Blood Flow and Metabolism. Conference: 24th International Symposium on Cerebral Blood Flow and Metabolism and the 9th International Conference on Quantification of Brain Function with PET. Chicago, IL United States. Conference Publication: (var.pagings). 29 (SUPPL. 1) (pp S577), 2009. Date of Publication: October 2009.
[Conference Abstract]
AN: 70031284
Publisher
Nature Publishing Group

Link to the Ovid Full Text or citation:
Click here for full text options

Link to the External Link Resolver:
SFX Link

383.

28th Annual Meeting of the European Association of Cardiothoracic Anaesthesiologists, EACTA 2013.
Anonymous
Embase
Applied Cardiopulmonary Pathophysiology. Conference: 28th Annual Meeting of the European Association of Cardiothoracic Anaesthesiologists, EACTA 2013. (28). Spain. Conference Publication: (234 pages). 17 (2) (pp 233-234) Date of Publication: 2013.
[Conference Review]
AN: 75005254
Publisher
PABST Science Publishers

Link to the Ovid Full Text or citation:
Click here for full text options

Link to the External Link Resolver:
SFX Link

384.

Cardiac magnetic resonance imaging in patients with coronary disease.
Wu H.D., Kwong R.Y.
Embase
Current Treatment Options in Cardiovascular Medicine. 10 (1) (pp 83-92), 2008. Date of Publication: February 2008.

[Review]
AN: 351247774
Publisher
Springer Healthcare

Link to the Ovid Full Text or citation:
Click here for full text options

Link to the External Link Resolver:
SFX Link

385.

Resting myocardial perfusion quantification with CMR arterial spin labeling at 1.5 T and 3.0 T.
Northrup B.E., McCommis K.S., Zhang H., Ray S., Woodard P.K., Gropler R.J., Zheng J.
Embase
Journal of Cardiovascular Magnetic Resonance. 10 (1) (no pagination), 2008. Article Number: 53. Date of Publication: 2008.
[Article]
AN: 355084820
Publisher
BioMed Central Ltd. (Floor 6, 236 Gray's Inn Road, London WC1X 8HB, United Kingdom)

Link to the Ovid Full Text or citation:
Click here for full text options

Link to the External Link Resolver:
SFX Link

386.

Fully automated motion correction in first-pass myocardial perfusion MR image sequences.
Milles J., van der Geest R.J., Jerosch-Herold M., Reiber J.H., Lelieveldt B.P.
Embase
IEEE transactions on medical imaging. 27 (11) (pp 1611-1621), 2008. Date of Publication: Nov 2008.
[Article]
AN: 550122175

Link to the Ovid Full Text or citation:
Click here for full text options

Link to the External Link Resolver:
SFX Link

387.

Glyph-based SPECT visualization for the diagnosis of coronary artery disease.
Meyer-Spradow J., Stegger L., Doring C., Ropinski T., Hinrichs K.
Embase
IEEE transactions on visualization and computer graphics. 14 (6) (pp 1499-1506), 2008. Date of Publication: 2008 Nov-Dec.
[Article]
AN: 550074651

Link to the Ovid Full Text or citation:
Click here for full text options

Link to the External Link Resolver:
SFX Link

388.

Toward realistic and practical ideal observer (IO) estimation for the optimization of medical imaging systems.
He X., Caffo B.S., Frey E.C.
Embase
IEEE transactions on medical imaging. 27 (10) (pp 1535-1543), 2008. Date of Publication: Oct 2008.
[Article]
AN: 352551336

Link to the Ovid Full Text or citation:
Click here for full text options

Link to the External Link Resolver:
SFX Link

389.

New methods for improved evaluation of patients with suspected acute coronary syndrome in the emergency department.
Ekelund U., Forberg J.L.
Embase
Postgraduate Medical Journal. 84 (988) (pp 83-86), 2008. Date of Publication: February 2008.
[Review]
AN: 351972491
Publisher
BMJ Publishing Group (Tavistock Square, London WC1H 9JR, United Kingdom)

Link to the Ovid Full Text or citation:
Click here for full text options

Link to the External Link Resolver:
SFX Link

390.

Methodology for quantiying absolute myocardial perfusion with PET and SPECT.
Lodge M.A., Bengel F.M.
Embase
Current Cardiology Reports. 9 (2) (pp 121-128), 2007. Date of Publication: April 2007.
[Review]
AN: 46681113
Publisher
Current Medicine Group LLC (5 Marine View Plaza, Suite 218, Hoboken NJ 07030, United States)

Link to the Ovid Full Text or citation:
Click here for full text options

Link to the External Link Resolver:
SFX Link

391.

Fully automated registration of first-pass myocardial perfusion MRI using independent component analysis.
Milles J., van der Geest R.J., Jerosch-Herold M., Reiber J.H., Lelieveldt B.P.
Embase
Information processing in medical imaging : proceedings of the ... conference. 20 (pp 544-555), 2007. Date of Publication: 2007.
[Article]
AN: 47349577

Link to the Ovid Full Text or citation:
Click here for full text options

Link to the External Link Resolver:
SFX Link

392.

Registration of cardiac SPECT/CT data through weighted intensity co-occurrence priors.
Guetter C., Wacker M., Xu C., Hornegger J.

Embase
Medical image computing and computer-assisted intervention : MICCAI ... International Conference on Medical Image Computing and Computer-Assisted Intervention. 10 (Pt 1) (pp 725-733), 2007. Date of Publication: 2007.
[Article]
AN: 350325817

Link to the Ovid Full Text or citation:
Click here for full text options

Link to the External Link Resolver:
SFX Link

393.

Attenuation resilient AIF estimation based on hierarchical Bayesian modelling for first pass myocardial perfusion MRI.
Schmid V.J., Gatehouse P.D., Yang G.Z.
Embase
Medical image computing and computer-assisted intervention : MICCAI ... International Conference on Medical Image Computing and Computer-Assisted Intervention. 10 (Pt 1) (pp 393-400), 2007. Date of Publication: 2007.
[Article]
AN: 350325777

Link to the Ovid Full Text or citation:
Click here for full text options

Link to the External Link Resolver:
SFX Link

394.

Soft level set coupling for LV segmentation in gated perfusion SPECT.
Kohlberger T., Funka-Lea G., Desh V.
Embase
Medical image computing and computer-assisted intervention : MICCAI ... International Conference on Medical Image Computing and Computer-Assisted Intervention. 10 (Pt 1) (pp 327-334), 2007. Date of Publication: 2007.
[Article]
AN: 350325769

Link to the Ovid Full Text or citation:
Click here for full text options

Link to the External Link Resolver:
SFX Link

395.

Use of neural networks to improve quality control of interpretations in myocardial perfusion imaging.
Tagil K., Marving J., Lomsky M., Hesse B., Edenbrandt L.
Embase
International Journal of Cardiovascular Imaging. 24 (8) (pp 841-848), 2008. Date of Publication: 2008.
[Article]
AN: 50192869
Publisher
Springer Netherlands (Van Godewijckstraat 30, Dordrecht 3311 GZ, Netherlands)

Link to the Ovid Full Text or citation:
Click here for full text options

Link to the External Link Resolver:
SFX Link

396.

Evaluation of a decision support system for interpretation of myocardial perfusion gated SPECT.
Lomsky M., Gjertsson P., Johansson L., Richter J., Ohlsson M., Tout D., Van Aswegen A., Underwood S.R., Edenbrandt L.
Embase
European Journal of Nuclear Medicine and Molecular Imaging. 35 (8) (pp 1523-1529), 2008. Date of Publication: August 2008.
[Article]
AN: 50113895
Publisher
Springer Verlag (Tiergartenstrasse 17, Heidelberg D-69121, Germany)

Link to the Ovid Full Text or citation:
Click here for full text options

Link to the External Link Resolver:
SFX Link

397.

Quantitative myocardial-perfusion SPECT: Comparison of three state-of-the-art software packages.
Wolak A., Slomka P.J., Fish M.B., Lorenzo S., Acampa W., Berman D.S., Germano G.
Embase
Journal of Nuclear Cardiology. 15 (1) (pp 27-34), 2008. Date of Publication: January

2008/February 2008.
[Article]
AN: 351168512
Publisher
Springer New York (233 Springer Street, New York NY 10013-1578, United States)

Link to the Ovid Full Text or citation:
Click here for full text options

Link to the External Link Resolver:
SFX Link

398.

Temperature disturbances in traumatic brain injury: Relationship to secondary insults, barbiturate treatment and outcome.
Elf K., Nilsson P., Ronne-Engstrom E., Howells T., Enblad P.
Embase
Neurological Research. 30 (10) (pp 1097-1105), 2008. Date of Publication: December 2008.
[Article]
AN: 352811103
Publisher
Maney Publishing (Suite 1C, Joseph's Well, Hanover Walk, Leeds LS3 1AB, United Kingdom)

Link to the Ovid Full Text or citation:
Click here for full text options

Link to the External Link Resolver:
SFX Link

399.

Contrast-dose relation in first-pass myocardial MR perfusion imaging.
Utz W., Niendorf T., Wassmuth R., Messroghli D., Dietz R., Schulz-Menger J.
Embase
Journal of Magnetic Resonance Imaging. 25 (6) (pp 1131-1135), 2007. Date of Publication: June 2007.
[Article]
AN: 46870268
Publisher
John Wiley and Sons Inc. (P.O.Box 18667, Newark NJ 07191-8667, United States)

Link to the Ovid Full Text or citation:
Click here for full text options

Link to the External Link Resolver:
SFX Link

400.

Quantification of myocardial blood volume during dipyridamole and doubtamine stress: A perfusion CMR study.
McCommis K.S., Goldstein T.A., Zhang H., Misselwitz B., Gropler R.J., Zheng J.
Embase
Journal of Cardiovascular Magnetic Resonance. 9 (5) (pp 785-792), 2007. Date of Publication: September 2007.
[Article]
AN: 47463902
Publisher
BioMed Central Ltd. (34 - 42 Cleveland Street, London W1T 4LB, United Kingdom)

Link to the Ovid Full Text or citation:
Click here for full text options

Link to the External Link Resolver:
SFX Link

401.

Myocardial perfusion assessment by use of system identification method in a one-compartment model.
Kocher M., Daire J.L., Thevenaz P., Schindler T., Keller P.F., Didier D., Vallee J.P.
Embase
Conference proceedings : ... Annual International Conference of the IEEE Engineering in Medicine and Biology Society. IEEE Engineering in Medicine and Biology Society. Conference. (pp 4492-4495), 2007. Date of Publication: 2007.
[Article]
AN: 350367355

Link to the Ovid Full Text or citation:
Click here for full text options

Link to the External Link Resolver:
SFX Link

402.

Multi-slice computed tomography pulmonary perfusion imaging and clinic cardiac function in patients with chronic cardiac insufficiency: A preliminary correlative study.
Song J.-H., Miao J.-T., Xie X.-Q.
Embase
Chinese Journal of Medical Imaging Technology. 23 (11) (pp 1628-1632), 2007. Date of Publication: November 2007.
[Article]

AN: 350267752
Publisher
Editorial Board of Chinese Journal of Medical Imaging Techno (PO Box 2712, Beijing 100080, China)

Link to the Ovid Full Text or citation:
Click here for full text options

Link to the External Link Resolver:
SFX Link

403.

Motion-compensated MR valve imaging with COMB tag tracking and super-resolution enhancement.
Dowsey A.W., Keegan J., Lerotic M., Thom S., Firmin D., Yang G.Z.
Embase
Medical image computing and computer-assisted intervention : MICCAI ... International Conference on Medical Image Computing and Computer-Assisted Intervention. 9 (Pt 2) (pp 364-371), 2006. Date of Publication: 2006.
[Article]
AN: 46538788

Link to the Ovid Full Text or citation:
Click here for full text options

Link to the External Link Resolver:
SFX Link

404.

Quantification of total and perfused blood vessels in murine skin autografts using a fluorescent double-labeling technique.
O'Ceallaigh S., Herrick S.E., Bluff J.E., McGrouther D.A., Ferguson M.W.J.
Embase
Plastic and Reconstructive Surgery. 117 (1) (pp 140-151), 2006. Date of Publication: January 2006.
[Article]
AN: 44391559
Publisher
Lippincott Williams and Wilkins (351 West Camden Street, Baltimore MD 21201-2436, United States)

Link to the Ovid Full Text or citation:
Click here for full text options

Link to the External Link Resolver:
SFX Link

405.

Association rule discovery with the train and test approach for heart disease prediction.
Ordonez C.
Embase
IEEE transactions on information technology in biomedicine : a publication of the IEEE Engineering in Medicine and Biology Society. 10 (2) (pp 334-343), 2006. Date of Publication: Apr 2006.
[Article]
AN: 43713859

Link to the Ovid Full Text or citation:
Click here for full text options

Link to the External Link Resolver:
SFX Link

406.

Evaluation of an algorithm for semiautomated segmentation of thin tissue layers in high-frequency ultrasound images.
Qiu Q., Dunmore-Buyze J., Boughner D.R., Lacefield J.C.
Embase
IEEE transactions on ultrasonics, ferroelectrics, and frequency control. 53 (2) (pp 324-334), 2006. Date of Publication: Feb 2006.
[Article]
AN: 43465032

Link to the Ovid Full Text or citation:
Click here for full text options

Link to the External Link Resolver:
SFX Link

407.

Tissue perfusion diagnostic classification using a spatio-temporal analysis of contrast ultrasound image sequences.
Williams Q., Noble J.A., Ehlgen A., Becher H.
Embase
Information processing in medical imaging : proceedings of the ... conference. 19 (pp 222-233), 2005. Date of Publication: 2005.
[Article]
AN: 46538699

Link to the Ovid Full Text or citation:
Click here for full text options

Link to the External Link Resolver:
SFX Link

408.

Automatic segmentation of the left ventricle in 3D SPECT data by registration with a dynamic anatomic model.
Dornheim L., Tonnies K.D., Dixon K.
Embase
Medical image computing and computer-assisted intervention : MICCAI ... International Conference on Medical Image Computing and Computer-Assisted Intervention. 8 (Pt 1) (pp 335-342), 2005. Date of Publication: 2005.
[Article]
AN: 43857896

Link to the Ovid Full Text or citation:
Click here for full text options

Link to the External Link Resolver:
SFX Link

409.

Cardiac CT: Where are we today and where are we going?.
Fishman E.K.
Embase
Applied Radiology. 35 (12 SUPPL.) (pp 5-9), 2006. Date of Publication: December 2006.
[Article]
AN: 46111564
Publisher
Anderson Publishing Ltd (180 Glenside Avenue, Scotch Plains NJ 07076, United States)

Link to the Ovid Full Text or citation:
Click here for full text options

Link to the External Link Resolver:
SFX Link

410.

Model-based image reconstruction for dynamic cardiac perfusion MRI from sparse data.

Awate S.P., DiBella E.V., Tasdizen T., Whitaker R.T.
Embase
Conference proceedings : ... Annual International Conference of the IEEE Engineering in Medicine and Biology Society. IEEE Engineering in Medicine and Biology Society. Conference. (pp 936-941), 2006. Date of Publication: 2006.
[Article]
AN: 350365120

Link to the Ovid Full Text or citation:
Click here for full text options

Link to the External Link Resolver:
SFX Link

411.

The added value of ECG-gating for the diagnosis of myocardial infarction using myocardial perfusion scintigraphy and artificial neural networks.
Gjertsson P., Lomsky M., Richter J., Ohlsson M., Tout D., van Aswegen A., Underwood R., Edenbrandt L.
Embase
Clinical Physiology and Functional Imaging. 26 (5) (pp 301-304), 2006. Date of Publication: September 2006.
[Article]
AN: 44269805
Publisher
Blackwell Publishing Ltd (9600 Garsington Road, Oxford OX4 2XG, United Kingdom)

Link to the Ovid Full Text or citation:
Click here for full text options

Link to the External Link Resolver:
SFX Link

412.

Myocardial perfusion quantification in patients suspected of cardiac syndrome X with positive and negative exercise testing: A [13N]ammonia positron emission tomography study.
De Vries J., DeJongste M.J.L., Jessurun G.A.J., Jager P.L., Staal M.J., Slart R.H.J.A.

Embase
Nuclear Medicine Communications. 27 (10) (pp 791-794), 2006. Date of Publication: October 2006.
[Article]
AN: 44401247
Publisher
Lippincott Williams and Wilkins (250 Waterloo Road, London SE1 8RD, United Kingdom)

Link to the Ovid Full Text or citation:
Click here for full text options

Link to the External Link Resolver:
SFX Link

413.

Patient gender and radiopharmaceutical tracer is of minor importance for the interpretation of myocardial perfusion images using an artificial neural network.
Tagil K., Underwood S.R., Davies G., Latus K.A., Ohlsson M., Gotborg C.W., Edenbrandt L.
Embase
Clinical Physiology and Functional Imaging. 26 (3) (pp 146-150), 2006. Date of Publication: May 2006.
[Article]
AN: 43615346
Publisher
Blackwell Publishing Ltd (9600 Garsington Road, Oxford OX4 2XG, United Kingdom)

Link to the Ovid Full Text or citation:
Click here for full text options

Link to the External Link Resolver:
SFX Link

414.

Myocardial perfusion imaging with Tc-99m MIBI in patients with left bundle branch block: Visual and quantitative assessment of anteroseptal perfusion for the diagnosis of left anterior descending coronary artery stenosis. Miokardiale perfusiebeelding met Tc-99m MIBI in pasiente met linker bondeltakblok: Die visuele en kwantitatiewe beoordeling van anteroseptale perfusie vir die diagnose van stenose van die linker afdalende arterie <Miokardiale perfusiebeelding met Tc-99m MIBI in pasiente met linker bondeltakblok: Die visuele en kwantitatiewe beoordeling van anteroseptale perfusie vir die diagnose van stenose van die linker afdalende arterie.>
Moller J., Warwick J., Bouma H.
Embase
Cardiovascular Journal of South Africa. 16 (2) (pp 95-101), 2005. Date of Publication: March/April 2005.
[Article]
AN: 41109927
Publisher
South African Medical Association (Private Bag X1, Pinelands 7430, South Africa)

Link to the Ovid Full Text or citation:
Click here for full text options

Link to the External Link Resolver:

SFX Link

415.

Non-invasive coronary angiography with multislice computed tomography. Technology, methods, preliminary experience and prospects.
Traversi E., Bertoli G., Barazzoni G., Baldi M., Tramarin R.
Embase
Italian heart journal : official journal of the Italian Federation of Cardiology. 5 (2) (pp 89-98), 2004. Date of Publication: Feb 2004.
[Review]
AN: 39260936

Link to the Ovid Full Text or citation:
Click here for full text options

Link to the External Link Resolver:
SFX Link

416.

A new method of color Doppler perfusion measurement via dynamic sonographic signal quantification in renal parenchyma.
Scholbach T., Dimos I., Scholbach J.
Embase
Nephron - Physiology. 96 (4) (pp p99-p104), 2004. Date of Publication: 2004.
[Article]
AN: 38586153
Publisher
S. Karger AG (Allschwilerstrasse 10, P.O. Box, Basel CH-4009, Switzerland)

Link to the Ovid Full Text or citation:
Click here for full text options

Link to the External Link Resolver:
SFX Link

417.

Myocardial perfusion measurements by spin-labeling under different vasodynamic states.
Fidler F., Wacker C.M., Dueren C., Weigel M., Jakob P.M., Bauer W.R., Haase A.
Embase
Journal of Cardiovascular Magnetic Resonance. 6 (2) (pp 509-516), 2004. Date of Publication: 2004.
[Article]
AN: 39277122

Publisher
BioMed Central Ltd. (34 - 42 Cleveland Street, London W1T 4LB, United Kingdom)

Link to the Ovid Full Text or citation:
Click here for full text options

Link to the External Link Resolver:
SFX Link

418.

Dynamic color Doppler sonography of intestinal wall in patients with Crohn disease compared with healthy subjects.
Scholbach T., Herrero I., Scholbach J.
Embase
Journal of pediatric gastroenterology and nutrition. 39 (5) (pp 524-528), 2004. Date of Publication: Nov 2004.
[Article]
AN: 39709652

Link to the Ovid Full Text or citation:
Click here for full text options

Link to the External Link Resolver:
SFX Link

419.

Use of high flip angle in T1-prepared FAST sequences for myocardial perfusion quantification.
Vallee J.-P., Ivancevic M., Lazeyras F., Kasuboski L., Chatelain P., Righetti A., Didier D.
Embase
European Radiology. 13 (3) (pp 507-514), 2003. Date of Publication: 01 Mar 2003.
[Article]
AN: 36790523
Publisher
Springer Verlag (Tiergartenstrasse 17, Heidelberg D-69121, Germany)

Link to the Ovid Full Text or citation:
Click here for full text options

Link to the External Link Resolver:
SFX Link

420.

Diagnosing shock via artificial intelligence: Applying machine learning techniques to medicine.
Tegetmeyer K., Massey B., Goldstein B.
Embase
Critical Care Medicine. 32 (2) (pp 602-603), 2004. Date of Publication: February 2004.
[Editorial]
AN: 38234155
Publisher
Lippincott Williams and Wilkins (351 West Camden Street, Baltimore MD 21201-2436, United States)

Link to the Ovid Full Text or citation:
Click here for full text options

Link to the External Link Resolver:
SFX Link

421.

Classification of ischemic dysfunctional myocardium combining perfusion quantification and contractile reserve evaluation using nitrate-enhanced gated single photon emission computed tomography with dobutamine test.
Sciagra R., Leoncini M., Mennuti A., Dabizzi R.P., Pupi A.
Embase
Quarterly Journal of Nuclear Medicine and Molecular Imaging. 48 (1) (pp 4-11), 2004. Date of Publication: March 2004.
[Article]
AN: 39069658
Publisher
Edizioni Minerva Medica S.p.A. (Corso Bramante 83-85, Torino 10126, Italy)

Link to the Ovid Full Text or citation:
Click here for full text options

Link to the External Link Resolver:
SFX Link

422.

Contrast echocardiography: New agents.
Miller A.P., Nanda N.C.
Embase
Ultrasound in Medicine and Biology. 30 (4) (pp 425-434), 2004. Date of Publication: April 2004.
[Review]
AN: 38581141
Publisher
Elsevier USA (6277 Sea Harbor Drive, Orlando FL 32862 8239, United States)

Link to the Ovid Full Text or citation:
Click here for full text options

Link to the External Link Resolver:
SFX Link

423.

CT attenuation correction for myocardial perfusion quantification using a PET/CT hybrid scanner.
Koepfli P., Hany T.F., Wyss C.A., Namdar M., Burger C., Konstantinidis A.V., Berthold T., Von Schulthess G.K., Kaufmann P.A.
Embase
Journal of Nuclear Medicine. 45 (4) (pp 537-542), 2004. Date of Publication: 01 Apr 2004.
[Article]
AN: 47618582
Publisher
Society of Nuclear Medicine Inc. (1850 Samuel Morse Drive, Reston VA 22090-5316, United States)

Link to the Ovid Full Text or citation:
Click here for full text options

Link to the External Link Resolver:
SFX Link

424.

Three-dimensional fusion of coronary arteries with myocardial perfusion distributions: clinical validation.
Faber T.L., Santana C.A., Garcia E.V., Candell-Riera J., Folks R.D., Peifer J.W., Hopper A., Aguade S., Angel J., Klein J.L.
Embase
Journal of Nuclear Medicine. 45 (5) (pp 745-753), 2004. Date of Publication: 01 May 2004.
[Article]
AN: 47618551
Publisher
Society of Nuclear Medicine Inc. (1850 Samuel Morse Drive, Reston VA 22090-5316, United States)

Link to the Ovid Full Text or citation:
Click here for full text options

Link to the External Link Resolver:
SFX Link

425.

Integration of clinical and imaging data to predict the presence of coronary artery disease with the use of neural networks.
Scott J.A., Aziz K., Yasuda T., Gewirtz H.
Embase
Coronary Artery Disease. 15 (7) (pp 427-434), 2004. Date of Publication: November 2004.
[Article]
AN: 39488768
Publisher
Lippincott Williams and Wilkins (250 Waterloo Road, London SE1 8RD, United Kingdom)

Link to the Ovid Full Text or citation:
Click here for full text options

Link to the External Link Resolver:
SFX Link

426.

The Artificial Organ's Last Frontier.
White R.J.
Embase
Artificial Organs. 28 (5) (pp 437-438), 2004. Date of Publication: May 2004.
[Editorial]
AN: 38680155
Publisher
Blackwell Publishing Inc. (350 Main Street, Malden MA 02148, United States)

Link to the Ovid Full Text or citation:
Click here for full text options

Link to the External Link Resolver:
SFX Link

427.

Prediction of left ventricular ejection fraction in patients with coronary artery disease based on an analysis of perfusion patterns at rest. Assessment by an artificial neural network.
Stefaniak B., Cholewinski W., Tarkowska A.
Embase
Nuclear Medicine Review. 7 (1) (pp 7-12), 2004. Date of Publication: 2004.
[Article]
AN: 38745223

Publisher
Via Medica (Ul. Swietokrzyska 73, Gdansk 80-180, Poland)

Link to the Ovid Full Text or citation:
Click here for full text options

Link to the External Link Resolver:
SFX Link

428.

WeAidU - A decision support system for myocardial perfusion images using artificial neural networks.
Ohlsson M.
Embase
Artificial Intelligence in Medicine. 30 (1) (pp 49-60), 2004. Date of Publication: January 2004.
[Article]
AN: 37543647
Publisher
Elsevier (P.O. Box 211, Amsterdam 1000 AE, Netherlands)

Link to the Ovid Full Text or citation:
Click here for full text options

Link to the External Link Resolver:
SFX Link

429.

Quantitative Assessment of Myocardial Perfusion With a Spin-Labeling Technique: Preliminary Results in Patients with Coronary Artery Disease.
Wacker C.M., Fidler F., Dueren C., Hirn S., Jakob P.M., Ertl G., Haase A., Bauer W.R.
Embase
Journal of Magnetic Resonance Imaging. 18 (5) (pp 555-560), 2003. Date of Publication: November 2003.
[Article]
AN: 37339800
Publisher
John Wiley and Sons Inc. (P.O.Box 18667, Newark NJ 07191-8667, United States)

Link to the Ovid Full Text or citation:
Click here for full text options

Link to the External Link Resolver:
SFX Link

430.

The Ruhr Center of Competence for Medical Enginnering (Kompetenzzentrum Medizintechnik Ruhr KMR, Bochum).
Ermert H.
Embase
Biomedizinische Technik. Biomedical engineering. 47 Suppl 1 Pt 2 (pp 886-889), 2002. Date of Publication: 2002.
[Article]
AN: 35557143

Link to the Ovid Full Text or citation:
Click here for full text options

Link to the External Link Resolver:
SFX Link

431.

Neuro-fuzzy systems for computer-aided myocardial viability assessment.
Behloul F., Lelieveldt B.P., Boudraa A., Janier M.F., Revel D., Reiber J.H.
Embase
IEEE transactions on medical imaging. 20 (12) (pp 1302-1313), 2001. Date of Publication: Dec 2001.
[Article]
AN: 35567173

Link to the Ovid Full Text or citation:
Click here for full text options

Link to the External Link Resolver:
SFX Link

432.

Improving the measurement of the 99Tc(m)-ECD brain perfusion index by temporal analysis.
Groiselle C., Rocchisani J.M., Moretti J.L.
Embase
Nuclear medicine communications. 21 (9) (pp 811-816), 2000. Date of Publication: Sep 2000.
[Article]
AN: 33413488

Link to the Ovid Full Text or citation:
Click here for full text options

Link to the External Link Resolver:
SFX Link

433.

Microbubbles-ultrasound interactions. Interactions microbulles/ultrasons
<Interactions microbulles/ultrasons.>
Lafitte S.
Embase
Annales de Cardiologie et d'Angeiologie. 51 (4) (pp 210-211), 2002. Date of Publication: September 2002.
[Conference Paper]
AN: 35118884
Publisher
Elsevier Masson SAS (62 rue Camille Desmoulins, Issy les Moulineaux Cedex 92442, France)

Link to the Ovid Full Text or citation:
Click here for full text options

Link to the External Link Resolver:
SFX Link

434.

Low-dose dobutamine nitrate-enhanced technetium 99m sestamibi gated SPECT versus low-dose dobutamine echocardiography for detecting reversible dysfunction in ischemic cardiomyopathy.
Leoncini M., Sciagra R., Bellandi F., Maioli M., Sestini S., Marcucci G., Coppola A., Frascarelli F., Mennuti A., Dabizzi R.P.
Embase
Journal of Nuclear Cardiology. 9 (4) (pp 402-406), 2002. Date of Publication: July/August 2002.
[Article]
AN: 35071393
Publisher
Springer New York (233 Springer Street, New York NY 10013-1578, United States)

Link to the Ovid Full Text or citation:
Click here for full text options

Link to the External Link Resolver:
SFX Link

435.

Quantification of SPECT myocardial perfusion imaging.
Acampa W., He W., Cuocolo A.
Embase
Journal of Nuclear Cardiology. 9 (3) (pp 338-342), 2002. Date of Publication: May/June 2002.
[Article]
AN: 35072005
Publisher
Springer New York (233 Springer Street, New York NY 10013-1578, United States)

Link to the Ovid Full Text or citation:
Click here for full text options

Link to the External Link Resolver:
SFX Link

436.

Value of exercise data for the interpretation of myocardial perfusion SPECT.
Haraldsson H., Ohlsson M., Edenbrandt L.
Embase
Journal of Nuclear Cardiology. 9 (2) (pp 169-173), 2002. Date of Publication: March/April 2002.
[Article]
AN: 35071986
Publisher
Springer New York (233 Springer Street, New York NY 10013-1578, United States)

Link to the Ovid Full Text or citation:
Click here for full text options

Link to the External Link Resolver:
SFX Link

437.

Interpretation and reporting of myocardial perfusion SPECT: A summary for technologists.
Folks R.D.
Embase
Journal of Nuclear Medicine Technology. 30 (4) (pp 153-164), 2002. Date of Publication: December 2002.
[Review]
AN: 35417720
Publisher
Society of Nuclear Medicine Inc. (1850 Samuel Morse Drive, Reston VA 22090-5316, United States)

Link to the Ovid Full Text or citation:

Click here for full text options

Link to the External Link Resolver:
SFX Link

438.

Knowledge discovery approach to automated cardiac SPECT diagnosis.
Kurgan L.A., Cios K.J., Tadeusiewicz R., Ogiela M., Goodenday L.S.
Embase
Artificial Intelligence in Medicine. 23 (2) (pp 149-169), 2001. Date of Publication: 2001.
[Article]
AN: 32907282
Publisher
Elsevier (P.O. Box 211, Amsterdam 1000 AE, Netherlands)

Link to the Ovid Full Text or citation:
Click here for full text options

Link to the External Link Resolver:
SFX Link

439.

Prediction of functional recovery in patients with chronic coronary artery disease and left ventricular dysfunction combining the evaluation of myocardial perfusion and of contractile reserve using nitrate-enhanced technetium-99m sestamibi gated single-photon emission computed tomography and dobutamine stress.
Leoncini M., Marcucci G., Sciagra R., Frascarelli F., Simonetti I., Bini L., Maioli M., Mennuti A., Dabizzi R.P.
Embase
American Journal of Cardiology. 87 (12) (pp 1346-1350), 2001. Date of Publication: 15 Jun 2001.
[Article]
AN: 32523016
Publisher
Elsevier Inc. (360 Park Avenue South, New York NY 10010, United States)

Link to the Ovid Full Text or citation:
Click here for full text options

Link to the External Link Resolver:
SFX Link

440.

Noninvasive monitoring in the pediatric intensive care unit.
DeNicola L.K., Kissoon N., Abram Jr. H.S., Sullivan K.J., Delgado-Corcoran C., Taylor C.
Embase
Pediatric Clinics of North America. 48 (3) (pp 573-588), 2001. Date of Publication: 2001.
[Review]
AN: 32521705
Publisher
W.B. Saunders (Independence Square West, Philadelphia PA 19106-3399, United States)

Link to the Ovid Full Text or citation:
[Click here for full text options]

Link to the External Link Resolver:
[SFX Link]

441.

[1-11C]acetate as a quantitative perfusion tracer in myocardial PET.
Van den Hoff J., Burchert W., Borner A.-R., Fricke H., Kuhnel G., Meyer G.J., Otto D., Weckesser E., Wolpers H.-G., Knapp W.H.
Embase
Journal of Nuclear Medicine. 42 (8) (pp 1174-1182), 2001. Date of Publication: 2001.
[Article]
AN: 32717834
Publisher
Society of Nuclear Medicine Inc. (1850 Samuel Morse Drive, Reston VA 22090-5316, United States)

Link to the Ovid Full Text or citation:
[Click here for full text options]

Link to the External Link Resolver:
[SFX Link]

442.

Diagnostic performance of an expert system for the interpretation of myocardial perfusion SPECT studies.
Garcia E.V., Cooke C.D., Folks R.D., Santana C.A., Krawczynska E.G., De Braal L., Ezquerra N.F.
Embase
Journal of Nuclear Medicine. 42 (8) (pp 1185-1191), 2001. Date of Publication: 2001.
[Article]
AN: 32717836
Publisher
Society of Nuclear Medicine Inc. (1850 Samuel Morse Drive, Reston VA 22090-5316, United States)

Link to the Ovid Full Text or citation:
Click here for full text options

Link to the External Link Resolver:
SFX Link

443.

The optimal reference population for cardiac normality in myocardial SPET in the detection of coronary artery stenoses: Patients with normal coronary angiography or subjects with low likelihood of coronary artery disease?.
Toft J., Lindahl D., Ohlsson M., Palmer J., Lundin A., Edenbrandt L., Hesse B.
Embase
European Journal of Nuclear Medicine. 28 (7) (pp 831-835), 2001. Date of Publication: 2001.
[Article]
AN: 32662545
Publisher
Springer Verlag (Tiergartenstrasse 17, Heidelberg D-69121, Germany)

Link to the Ovid Full Text or citation:
Click here for full text options

Link to the External Link Resolver:
SFX Link

444.

A fast nonlinear method for parametric imaging of myocardial perfusion by dynamic 13N-ammonia PET.
Golish S.R., Hove J.D., Schelbert H.R., Gambhir S.S.
Embase
Journal of Nuclear Medicine. 42 (6) (pp 924-931), 2001. Date of Publication: 2001.
[Article]
AN: 32537441
Publisher
Society of Nuclear Medicine Inc. (1850 Samuel Morse Drive, Reston VA 22090-5316, United States)

Link to the Ovid Full Text or citation:
Click here for full text options

Link to the External Link Resolver:
SFX Link

445.

Scandinavian test of artificial neural network for classification of myocardial perfusion images.
Lindahl D., Toft J., Hesse B., Palmer J., Ali S., Lundin A., Edenbrandt L.
Embase
Clinical Physiology. 20 (4) (pp 253-261), 2000. Date of Publication: July 2000.
[Article]
AN: 30456122
Publisher
Blackwell Publishing Ltd. (9600 Garsington Road, Oxford OX4 2XG, United Kingdom)

Link to the Ovid Full Text or citation:
Click here for full text options

Link to the External Link Resolver:
SFX Link

446.

Left ventricular function and perfusion from gated SPECT perfusion images: An integrated method.
Faber T.L., Cooke C.D., Folks R.D., Vansant J.P., Nichols K.J., DePuey E.G., Pettigrew R.I., Garcia E.V.
Embase
Journal of Nuclear Medicine. 40 (4) (pp 650-659), 1999. Date of Publication: April 1999.
[Article]
AN: 29171808
Publisher
Society of Nuclear Medicine Inc. (1850 Samuel Morse Drive, Reston VA 22090-5316, United States)

Link to the Ovid Full Text or citation:
Click here for full text options

Link to the External Link Resolver:
SFX Link

447.

Magnetic resonance perfusion imaging in ischemic heart disease.
Canet E.P., Janier M.F., Revel D.
Embase
Journal of Magnetic Resonance Imaging. 10 (3) (pp 423-433), 1999. Date of Publication: September 1999.
[Article]
AN: 29463128
Publisher

John Wiley and Sons Inc. (P.O.Box 18667, Newark NJ 07191-8667, United States)

Link to the Ovid Full Text or citation:
Click here for full text options

Link to the External Link Resolver:
SFX Link

448.

Quantification of myocardial perfusion with FAST sequence and Gd bolus in patients with normal cardiac function.
Vallee J.-P., Lazeyras F., Kasuboski L., Chatelain P., Howarth N., Righetti A., Didier D.
Embase
Journal of Magnetic Resonance Imaging. 9 (2) (pp 197-203), 1999. Date of Publication: February 1999.
[Article]
AN: 29115341
Publisher
John Wiley and Sons Inc. (P.O.Box 18667, Newark NJ 07191-8667, United States)

Link to the Ovid Full Text or citation:
Click here for full text options

Link to the External Link Resolver:
SFX Link

449.

Improved classifications of myocardial bull's-eye scintigrams with computer-based decision support system.
Lindahl D., Lanke J., Lundin A., Palmer J., Edenbrandt L.
Embase
Journal of Nuclear Medicine. 40 (1) (pp 96-101), 1999. Date of Publication: January 1999.
[Article]
AN: 29044179
Publisher
Society of Nuclear Medicine Inc. (1850 Samuel Morse Drive, Reston VA 22090-5316, United States)

Link to the Ovid Full Text or citation:
Click here for full text options

Link to the External Link Resolver:
SFX Link

450.

Defining pulsatile perfusion: Quantification in terms of energy equivalent pressure.
Undar A., Frazier O.H., Fraser Jr. C.D.
Embase
Artificial Organs. 23 (8) (pp 712-716), 1999. Date of Publication: 1999.
[Article]
AN: 29383311
Publisher
Blackwell Publishing Inc. (350 Main Street, Malden MA 02148, United States)

Link to the Ovid Full Text or citation:
Click here for full text options

Link to the External Link Resolver:
SFX Link

451.

Myocardial SPET: Artificial neural networks describe extent and severity of perfusion defects.
Lindahl D., Palmer J., Edenbrandt L.
Embase
Clinical Physiology. 19 (6) (pp 497-503), 1999. Date of Publication: November 1999.
[Article]
AN: 29570465
Publisher
Blackwell Publishing Ltd. (9600 Garsington Road, Oxford OX4 2XG, United Kingdom)

Link to the Ovid Full Text or citation:
Click here for full text options

Link to the External Link Resolver:
SFX Link

452.

Quantification of myocardial perfusion by MRI after coronary occlusion.
Vallee J.-P.M., Sostman H.D., MacFall J.R., DeGrado T.R., Zhang J., Sebbag L., Cobb F.R., Wheeler T., Hedlund L.W., Turkington T.G., Spritzer C.E., Coleman R.E.
Embase
Magnetic Resonance in Medicine. 40 (2) (pp 287-297), 1998. Date of Publication: August 1998.
[Article]
AN: 28361532
Publisher

John Wiley and Sons Inc. (P.O.Box 18667, Newark NJ 07191-8667, United States)

Link to the Ovid Full Text or citation:
Click here for full text options

Link to the External Link Resolver:
SFX Link

453.

An assessment of two methods for generating automatic regions of interest.
Houston A.S., White D.R.R., Sampson W.F.D., Macleod M.A., Pilkington J.B.
Embase
Nuclear Medicine Communications. 19 (10) (pp 1005-1016), 1998. Date of Publication: 1998.
[Article]
AN: 28520150
Publisher
Lippincott Williams and Wilkins (250 Waterloo Road, London SE1 8RD, United Kingdom)

Link to the Ovid Full Text or citation:
Click here for full text options

Link to the External Link Resolver:
SFX Link

454.

Scintigraphic diagnosis of coronary artery disease: Myocardial bull's-eye images contain the important information.
Lindahl D., Palmer J., Pettersson J., White T., Lundin A., Edenbrandt L.
Embase
Clinical Physiology. 18 (6) (pp 554-561), 1998. Date of Publication: 1998.
[Article]
AN: 28488453
Publisher
Blackwell Publishing Ltd. (9600 Garsington Road, Oxford OX4 2XG, United Kingdom)

Link to the Ovid Full Text or citation:
Click here for full text options

Link to the External Link Resolver:
SFX Link

455.

Automated interpretation of myocardial SPECT perfusion images using artificial neural networks.
Lindahl D., Palmer J., Ohlsson M., Peterson C., Lundin A., Edenbrandt L.
Embase
Journal of Nuclear Medicine. 38 (12) (pp 1870-1875), 1997. Date of Publication: December 1997.
[Article]
AN: 28030913
Publisher
Society of Nuclear Medicine Inc. (1850 Samuel Morse Drive, Reston VA 22090-5316, United States)

Link to the Ovid Full Text or citation:
Click here for full text options

Link to the External Link Resolver:
SFX Link

456.

Identifying coronary stenosis using an image-recognition neural network.
Goodenday L.S., Cios K.J., Shin I.
Embase
IEEE Engineering in Medicine and Biology Magazine. 16 (5) (pp 139-144), 1997. Date of Publication: September/October 1997.
[Article]
AN: 27413187
Publisher
Institute of Electrical and Electronics Engineers Inc. (3 Park Avenue, 17th Floor, New York NY 10016-5997, United States)

Link to the Ovid Full Text or citation:
Click here for full text options

Link to the External Link Resolver:
SFX Link

457.

Three-dimensional mapping of acute ischemic regions using artificial neural networks and tagged MRI.
Azhari H., Oliker S., Rogers W.J., Weiss J.L., Shapiro E.P.
Embase
IEEE Transactions on Biomedical Engineering. 43 (6) (pp 619-626), 1996. Date of Publication: Jun.
[Article]
AN: 26158675
Publisher

IEEE Computer Society (445 Hoes Lane - P.O.Box 1331, Piscataway NJ 08855-1331, United States)

Link to the Ovid Full Text or citation:
Click here for full text options

Link to the External Link Resolver:
SFX Link

458.

Identification of a hypoperfused segment in bull's-eye myocardial perfusion images using a feed forward neural network.
Hamilton D., Riley P.J., Miola U.J., Amro A.A.
Embase
British Journal of Radiology. 68 (815) (pp 1208-1211), 1995. Date of Publication: 1995.
[Article]
AN: 25343628
Publisher
British Institute of Radiology (36 Portland Place, London W1N 1AT, United Kingdom)

Link to the Ovid Full Text or citation:
Click here for full text options

Link to the External Link Resolver:
SFX Link

459.

Three-dimensional displays of left ventricular epicardial surface from standard cardiac SPECT perfusion quantification techniques.
Faber T.L., Cooke C.D., Peifer J.W., Pettigrew R.I., Vansant J.P., Leyendecker J.R., Garcia E.V., Links J.M., Devous Sr. M.D.
Embase
Journal of Nuclear Medicine. 36 (4) (pp 697-703), 1995. Date of Publication: 1995.
[Article]
AN: 25111109
Publisher
Society of Nuclear Medicine Inc. (1850 Samuel Morse Drive, Reston VA 22090-5316, United States)

Link to the Ovid Full Text or citation:
Click here for full text options

Link to the External Link Resolver:
SFX Link

460.

A feed forward neural network for classification of bull's-eye myocardial perfusion images.
Hamilton D., Riley P.J., Miola U.J., Amro A.A.
Embase
European Journal of Nuclear Medicine. 22 (2) (pp 108-115), 1995. Date of Publication: 1995.
[Article]
AN: 25073733
Publisher
Springer Verlag (Tiergartenstrasse 17, Heidelberg D-69121, Germany)

Link to the Ovid Full Text or citation:
Click here for full text options

Link to the External Link Resolver:
SFX Link

461.

The use of computer-assisted diagnosis in cardiac perfusion nuclear medicine studies: a review (Part 3).
Datz F.L., Rosenberg C., Gabor F.V., Christian P.E., Gullberg G.T., Ahluwalia R., Morton K.A.
Embase
Journal of digital imaging : the official journal of the Society for Computer Applications in Radiology. 6 (2) (pp 67-80), 1993. Date of Publication: May 1993.
[Review]
AN: 23906061

Link to the Ovid Full Text or citation:
Click here for full text options

Link to the External Link Resolver:
SFX Link

462.

Hemodynamic and oxygen transport patterns for outcome prediction, therapeutic goals, and clinical algorithms to improve outcome. Feasibility of artificial intelligence to customize algorithms.
Shoemaker W.C., Patil R., Appel P.L., Kram H.B.
Embase
Chest. 102 (5 Suppl 2) (pp 617S-625S), 1992. Date of Publication: Nov 1992.
[Article]
AN: 22980699

Link to the Ovid Full Text or citation:
Click here for full text options

Link to the External Link Resolver:
SFX Link

463.

Three-dimensional motion and perfusion quantification in gated single- photon emission computed tomograms.
Faber T.L., Akers M.S., Peshock R.M., Corbett J.R.
Embase
Journal of Nuclear Medicine. 32 (12) (pp 2311-2317), 1991. Date of Publication: 1991.
[Article]
AN: 22022209
Publisher
Society of Nuclear Medicine Inc. (1850 Samuel Morse Drive, Reston VA 22090-5316, United States)

Link to the Ovid Full Text or citation:
Click here for full text options

Link to the External Link Resolver:
SFX Link

464.

Technical aspects of myocardial SPECT imaging with technetium-99m sestamibi.
Garcia E.V., Cooke C.D., Van Train K.F., Folks R., Peifer J., DePuey E.G., Maddahi J., Alazraki N., Galt J., Ezquerra N., Ziffer J., Areeda J., Berman D.S.
Embase
American Journal of Cardiology. 66 (13) (pp 23E-31E), 1990. Date of Publication: 1990.
[Conference Paper]
AN: 20369700
Publisher
Elsevier Inc. (360 Park Avenue South, New York NY 10010, United States)

Link to the Ovid Full Text or citation:
Click here for full text options

Link to the External Link Resolver:
SFX Link

465.

Three-dimensional techniques and artificial intelligence in thallium-201 cardiac imaging.
DePuey E.G., Garcia E.V., Ezquerra N.F.
Embase
American Journal of Roentgenology. 152 (6) (pp 1161-1168), 1989. Date of Publication: 1989.
[Review]
AN: 19140942
Publisher
American Roentgen Ray Society (44221 Slatestone Court, Leesburg VA 20176-5109, United States)

Link to the Ovid Full Text or citation:
Click here for full text options

Link to the External Link Resolver:
SFX Link

466.

Expansion of extracellular tracer spaces in the isolated heart perfused with crystalloid solutions: Expansion of extracellular space, trans-sarcolemmal leakage, or both?.
Polimeni P.I., Buraczewski S.I.
Embase
Journal of Molecular and Cellular Cardiology. 20 (1) (pp 15-22), 1988. Date of Publication: 1988.
[Article]
AN: 18118601
Publisher
Academic Press (24-28 Oval Road, London NW1 7DX, United Kingdom)

Link to the Ovid Full Text or citation:
Click here for full text options

Link to the External Link Resolver:
SFX Link

467.

Prognostically safe stress-only single-photon emission computed tomography myocardial perfusion imaging guided by machine learning: report from REFINE SPECT.
Hu LH; Miller RJH; Sharir T; Commandeur F; Rios R; Einstein AJ; Fish MB; Ruddy TD; Kaufmann PA; Sinusas AJ; Miller EJ; Bateman TM; Dorbala S; Di Carli M; Liang JX; Eisenberg E; Dey D; Berman DS; Slomka PJ.
Ovid MEDLINE(R) and Epub Ahead of Print, In-Process & Other Non-Indexed Citations and Daily
European heart journal cardiovascular Imaging. 2020 Jun 12.

[Journal Article]
UI: 32533137
Authors Full Name
Hu, Lien-Hsin; Miller, Robert J H; Sharir, Tali; Commandeur, Frederic; Rios, Richard; Einstein, Andrew J; Fish, Mathews B; Ruddy, Terrence D; Kaufmann, Philipp A; Sinusas, Albert J; Miller, Edward J; Bateman, Timothy M; Dorbala, Sharmila; Di Carli, Marcelo; Liang, Joanna X; Eisenberg, Evann; Dey, Damini; Berman, Daniel S; Slomka, Piotr J.

Link to the Ovid Full Text or citation:
Click here for full text options

Link to the External Link Resolver:
SFX Link

468.

Generalization Evaluation of Machine Learning Numerical Observers for Image Quality Assessment.
Kalayeh MM; Marin T; Brankov JG.
Ovid MEDLINE(R) and Epub Ahead of Print, In-Process & Other Non-Indexed Citations and Daily
IEEE Transactions on Nuclear Science. 60(3):1609-1618, 2013 Jun.
[Journal Article]
UI: 25346545
Authors Full Name
Kalayeh, Mahdi M; Marin, Thibault; Brankov, Jovan G.

Link to the Ovid Full Text or citation:
Click here for full text options

Link to the External Link Resolver:
SFX Link

469.

Predicting PET Cerebrovascular Reserve with Deep Learning by Using Baseline MRI: A Pilot Investigation of a Drug-Free Brain Stress Test.
Chen DYT; Ishii Y; Fan AP; Guo J; Zhao MY; Steinberg GK; Zaharchuk G.
Ovid MEDLINE(R) and Epub Ahead of Print, In-Process & Other Non-Indexed Citations and Daily
Radiology. 296(3):627-637, 2020 Sep.
[Journal Article]
UI: 32662761
Authors Full Name
Chen, David Y T; Ishii, Yosuke; Fan, Audrey P; Guo, Jia; Zhao, Moss Y; Steinberg, Gary K; Zaharchuk, Greg.

Link to the Ovid Full Text or citation:

Click here for full text options

Link to the External Link Resolver:
SFX Link

470.

Improved myocardial perfusion PET imaging using artificial neural networks.
Wang X; Yang B; Moody JB; Tang J.
Ovid MEDLINE(R) and Epub Ahead of Print, In-Process & Other Non-Indexed Citations and Daily
Physics in Medicine & Biology. 65(14):145010, 2020 07 20.
[Journal Article. Research Support, U.S. Gov't, Non-P.H.S.]
UI: 32244234
Authors Full Name
Wang, Xinhui; Yang, Bao; Moody, Jonathan B; Tang, Jing.

Link to the Ovid Full Text or citation:
Click here for full text options

Link to the External Link Resolver:
SFX Link

471.

A non-linear mathematical model using optical sensor to predict heart decellularization efficacy.
Pereira RHA; Prado AR; Caro LFCD; Zanardo TEC; Alencar AP; Nogueira BV.
Ovid MEDLINE(R) and Epub Ahead of Print, In-Process & Other Non-Indexed Citations and Daily
Scientific Reports. 9(1):12211, 2019 08 21.
[Journal Article. Research Support, Non-U.S. Gov't]
UI: 31434981
Authors Full Name
Pereira, Rayssa Helena Arruda; Prado, Adilson Ribeiro; Caro, Luiz Felipe Castello Del; Zanardo, Tadeu Eriton Caliman; Alencar, Airlane Pereira; Nogueira, Breno Valentim.

Link to the Ovid Full Text or citation:
Click here for full text options

Link to the External Link Resolver:
SFX Link

472.

Effect of Beam Hardening on Transmural Myocardial Perfusion Quantification in

Myocardial CT Imaging.
Fahmi R; Eck BL; Levi J; Fares A; Wu H; Vembar M; Dhanantwari A; Bezerra HG; Wilson DL.
Ovid MEDLINE(R) and Epub Ahead of Print, In-Process & Other Non-Indexed Citations and Daily
Proceedings of SPIE - the International Society for Optical Engineering. 9788, 2016 Mar.
[Journal Article]
UI: 32210495
Authors Full Name
Fahmi, Rachid; Eck, Brendan L; Levi, Jacob; Fares, Anas; Wu, Hao; Vembar, Mani; Dhanantwari, Amar; Bezerra, Hiram G; Wilson, David L.

Link to the Ovid Full Text or citation:
Click here for full text options

Link to the External Link Resolver:
SFX Link

473.

Corrigendum to "Automatic myocardial ischemic lesion detection on magnetic resonance perfusion weighted imaging prior perfusion quantification: A pre-modeling strategy" [Comput. Biol. Med. 110 (2019) 108-119].
Daviller C; Grenier T; Ratiney H; Sdika M; Croisille P; Viallon M.
Ovid MEDLINE(R) and Epub Ahead of Print, In-Process & Other Non-Indexed Citations and Daily
Computers in Biology & Medicine. 114:103455, 2019 Nov.
[Published Erratum]
UI: 31586780
Title Comment
[Erratum for: Comput Biol Med. 2019 Jul;110:108-119; PMID: 31153004 [https://www.ncbi.nlm.nih.gov/pubmed/31153004]]
Authors Full Name
Daviller, Clement; Grenier, Thomas; Ratiney, Helene; Sdika, Michael; Croisille, Pierre; Viallon, Magalie.

Link to the Ovid Full Text or citation:
Click here for full text options

Link to the External Link Resolver:
SFX Link

474.

Machine Learning based SpO2 Computation Using Reflectance Pulse Oximetry.
Venkat S; Arsath P S MTPS; Alex A; S P P; Balamugesh; D J C; Joseph J; Sivaprakasam M.
Ovid MEDLINE(R) and Epub Ahead of Print, In-Process & Other Non-Indexed Citations and Daily

Conference Proceedings: ... Annual International Conference of the IEEE Engineering in Medicine & Biology Society. 2019:482-485, 2019 07.
[Journal Article]
UI: 31945942
Authors Full Name
Venkat, Swaathi; Arsath P S, Mohamed Tanveejul P S; Alex, Annamol; S P, Preejith; Balamugesh; D J, Christopher; Joseph, Jayaraj; Sivaprakasam, Mohanasankar.

Link to the Ovid Full Text or citation:
Click here for full text options

Link to the External Link Resolver:
SFX Link

475.

Machine learning derived segmentation of phase velocity encoded cardiovascular magnetic resonance for fully automated aortic flow quantification.
Bratt A; Kim J; Pollie M; Beecy AN; Tehrani NH; Codella N; Perez-Johnston R; Palumbo MC; Alakbarli J; Colizza W; Drexler IR; Azevedo CF; Kim RJ; Devereux RB; Weinsaft JW.
Ovid MEDLINE(R) and Epub Ahead of Print, In-Process & Other Non-Indexed Citations and Daily
Journal of Cardiovascular Magnetic Resonance. 21(1):1, 2019 01 07.
[Journal Article. Multicenter Study. Research Support, N.I.H., Extramural. Validation Study]
UI: 30612574
Authors Full Name
Bratt, Alex; Kim, Jiwon; Pollie, Meridith; Beecy, Ashley N; Tehrani, Nathan H; Codella, Noel; Perez-Johnston, Rocio; Palumbo, Maria Chiara; Alakbarli, Javid; Colizza, Wayne; Drexler, Ian R; Azevedo, Clerio F; Kim, Raymond J; Devereux, Richard B; Weinsaft, Jonathan W.

Link to the Ovid Full Text or citation:
Click here for full text options

Link to the External Link Resolver:
SFX Link

476.

Myocardial perfusion quantification using simultaneously acquired 13 NH3 -ammonia PET and dynamic contrast-enhanced MRI in patients at rest and stress.
Kunze KP; Nekolla SG; Rischpler C; Zhang SH; Hayes C; Langwieser N; Ibrahim T; Laugwitz KL; Schwaiger M.
Ovid MEDLINE(R) and Epub Ahead of Print, In-Process & Other Non-Indexed Citations and Daily
Magnetic Resonance in Medicine. 80(6):2641-2654, 2018 12.
[Journal Article. Research Support, Non-U.S. Gov't]
UI: 29672922

Authors Full Name
Kunze, Karl P; Nekolla, Stephan G; Rischpler, Christoph; Zhang, Shelley HuaLei; Hayes, Carmel; Langwieser, Nicolas; Ibrahim, Tareq; Laugwitz, Karl-Ludwig; Schwaiger, Markus.

Link to the Ovid Full Text or citation:
Click here for full text options

Link to the External Link Resolver:
SFX Link

477.

Could Deep Learning Change Our Working Lives?.
Sabharwal NK.
Ovid MEDLINE(R) and Epub Ahead of Print, In-Process & Other Non-Indexed Citations and Daily
Jacc: Cardiovascular Imaging. 11(11):1664-1665, 2018 11.
[Editorial. Comment]
UI: 29550322
Title Comment
[Comment on: JACC Cardiovasc Imaging. 2018 Nov;11(11):1654-1663; PMID: 29550305 [https://www.ncbi.nlm.nih.gov/pubmed/29550305]]
Authors Full Name
Sabharwal, Nikant K.

Link to the Ovid Full Text or citation:
Click here for full text options

Link to the External Link Resolver:
SFX Link

478.

Can a Machine Learn Better Than Humans?.
Shaw LJ.
Ovid MEDLINE(R) and Epub Ahead of Print, In-Process & Other Non-Indexed Citations and Daily
Jacc: Cardiovascular Imaging. 11(7):1010-1011, 2018 07.
[Editorial. Comment]
UI: 29055636
Title Comment
[Comment on: JACC Cardiovasc Imaging. 2018 Jul;11(7):1000-1009; PMID: 29055639 [https://www.ncbi.nlm.nih.gov/pubmed/29055639]]
Authors Full Name
Shaw, Leslee J.

Link to the Ovid Full Text or citation:
Click here for full text options

Link to the External Link Resolver:
SFX Link

479.

Comparison of the diagnostic accuracies of very low stress-dose with standard-dose myocardial perfusion imaging: Automated quantification of one-day, stress-first SPECT using a CZT camera.
Sharir T; Pinskiy M; Pardes A; Rochman A; Prokhorov V; Kovalski G; Merzon K; Bojko A; Brodkin B.
Ovid MEDLINE(R) and Epub Ahead of Print, In-Process & Other Non-Indexed Citations and Daily
Journal of Nuclear Cardiology. 23(1):11-20, 2016 Feb.
[Comparative Study. Evaluation Study. Journal Article]
UI: 26012642
Title Comment
[Comment in: J Nucl Cardiol. 2016 Feb;23(1):21-3; PMID: 25971989 [https://www.ncbi.nlm.nih.gov/pubmed/25971989]]
Authors Full Name
Sharir, Tali; Pinskiy, Marina; Pardes, Abraham; Rochman, Arik; Prokhorov, Vitali; Kovalski, Gil; Merzon, Konstantine; Bojko, Andrzej; Brodkin, Boris.

Link to the Ovid Full Text or citation:
Click here for full text options

Link to the External Link Resolver:
SFX Link

480.

Myocardial blood flow quantification by Rb-82 cardiac PET/CT: A detailed reproducibility study between two semi-automatic analysis programs.
Dunet V; Klein R; Allenbach G; Renaud J; deKemp RA; Prior JO.
Ovid MEDLINE(R) and Epub Ahead of Print, In-Process & Other Non-Indexed Citations and Daily
Journal of Nuclear Cardiology. 23(3):499-510, 2016 06.
[Comparative Study. Evaluation Study. Journal Article. Validation Study]
UI: 25995182
Title Comment
[Comment in: J Nucl Cardiol. 2016 Jun;23(3):511-3; PMID: 25995184 [https://www.ncbi.nlm.nih.gov/pubmed/25995184]]
Authors Full Name
Dunet, Vincent; Klein, Ran; Allenbach, Gilles; Renaud, Jennifer; deKemp, Robert A; Prior, John O.

Link to the Ovid Full Text or citation:
Click here for full text options

Link to the External Link Resolver:

SFX Link

481.

Quantitative iodine-123-metaiodobenzylguanidine (MIBG) SPECT imaging in heart failure with left ventricular systolic dysfunction: Development and validation of automated procedures in conjunction with technetium-99m tetrofosmin myocardial perfusion SPECT.
Clements IP; Garcia EV; Chen J; Folks RD; Butler J; Jacobson AF.
Ovid MEDLINE(R) and Epub Ahead of Print, In-Process & Other Non-Indexed Citations and Daily
Journal of Nuclear Cardiology. 23(3):425-35, 2016 06.
[Evaluation Study. Journal Article. Validation Study]
UI: 25788403
Title Comment
[Comment in: J Nucl Cardiol. 2016 Jun;23(3):436-41; PMID: 25802176 [
https://www.ncbi.nlm.nih.gov/pubmed/25802176]]
Authors Full Name
Clements, Ian P; Garcia, Ernest V; Chen, Ji; Folks, Russell D; Butler, Javed; Jacobson, Arnold F.

Link to the Ovid Full Text or citation:
Click here for full text options

Link to the External Link Resolver:
SFX Link

482.

Coronary Computed Tomography Angiography: Enhancing Risk Stratification and Diagnosis of Cardiovascular Disease in Women. [Review]
Karnib S; Chinnaiyan KM.
Ovid MEDLINE(R) and Epub Ahead of Print, In-Process & Other Non-Indexed Citations and Daily
Current Treatment Options in Cardiovascular Medicine. 21(10):62, 2019 Oct 04.
[Journal Article. Review]
UI: 31584125
Authors Full Name
Karnib, Sara; Chinnaiyan, Kavitha M.

Link to the Ovid Full Text or citation:
Click here for full text options

Link to the External Link Resolver:
SFX Link

483.

Novel SPECT Technologies and Approaches in Cardiac Imaging.
Slomka P; Hung GU; Germano G; Berman DS.
Ovid MEDLINE(R) and Epub Ahead of Print, In-Process & Other Non-Indexed Citations and Daily
Cardiovascular Innovations & Applications. 2(1):31-46, 2016 Dec 01.
[Journal Article]
UI: 29034066
Authors Full Name
Slomka, Piotr; Hung, Guang-Uei; Germano, Guido; Berman, Daniel S.

Link to the Ovid Full Text or citation:
Click here for full text options

Link to the External Link Resolver:
SFX Link

484.

Fuzzy Rule-Based Classification System for Assessing Coronary Artery Disease.
Mohammadpour RA; Abedi SM; Bagheri S; Ghaemian A.
Ovid MEDLINE(R) and Epub Ahead of Print, In-Process & Other Non-Indexed Citations and Daily
Computational & Mathematical Methods in Medicine. 2015:564867, 2015.
[Journal Article. Research Support, Non-U.S. Gov't]
UI: 26448783
Authors Full Name
Mohammadpour, Reza Ali; Abedi, Seyed Mohammad; Bagheri, Somayeh; Ghaemian, Ali.

Link to the Ovid Full Text or citation:
Click here for full text options

Link to the External Link Resolver:
SFX Link

485.

Validation of an automated method to quantify stress-induced ischemia and infarction in rest-stress myocardial perfusion SPECT.
Fransson H; Ljungberg M; Carlsson M; Engblom H; Arheden H; Heiberg E.
Ovid MEDLINE(R) and Epub Ahead of Print, In-Process & Other Non-Indexed Citations and Daily
Journal of Nuclear Cardiology. 21(3):503-18, 2014 Jun.
[Journal Article. Randomized Controlled Trial. Research Support, Non-U.S. Gov't. Validation Study]
UI: 24532031
Authors Full Name
Fransson, Helen; Ljungberg, Michael; Carlsson, Marcus; Engblom, Henrik; Arheden, Hakan; Heiberg, Einar.

Link to the Ovid Full Text or citation:
Click here for full text options

Link to the External Link Resolver:
SFX Link

486.

Quantitative analysis of perfusion studies: strengths and pitfalls. [Review]
Slomka P; Xu Y; Berman D; Germano G.
Ovid MEDLINE(R) and Epub Ahead of Print, In-Process & Other Non-Indexed Citations and Daily
Journal of Nuclear Cardiology. 19(2):338-46, 2012 Apr.
[Journal Article. Review]
UI: 22302181
Authors Full Name
Slomka, Piotr; Xu, Yuan; Berman, Daniel; Germano, Guido.

Link to the Ovid Full Text or citation:
Click here for full text options

Link to the External Link Resolver:
SFX Link

487.

MRF-based intensity invariant elastic registration of cardiac perfusion images using saliency information.
Mahapatra D; Sun Y.
Ovid MEDLINE(R) and Epub Ahead of Print, In-Process & Other Non-Indexed Citations and Daily
IEEE Transactions on Biomedical Engineering. 58(4):991-1000, 2011 Apr.
[Journal Article. Research Support, Non-U.S. Gov't]
UI: 21097377
Authors Full Name
Mahapatra, Dwarikanath; Sun, Ying.

Link to the Ovid Full Text or citation:
Click here for full text options

Link to the External Link Resolver:
SFX Link

488.

Fully automatic registration and segmentation of first-pass myocardial perfusion MR image sequences.
Gupta V; Hendriks EA; Milles J; van der Geest RJ; Jerosch-Herold M; Reiber JH; Lelieveldt BP.
Ovid MEDLINE(R) and Epub Ahead of Print, In-Process & Other Non-Indexed Citations and Daily
Academic Radiology. 17(11):1375-85, 2010 Nov.
[Evaluation Study. Journal Article]
UI: 20801696
Authors Full Name
Gupta, Vikas; Hendriks, Emile A; Milles, Julien; van der Geest, Rob J; Jerosch-Herold, Michael; Reiber, Johan H C; Lelieveldt, Boudewijn P F.

Link to the Ovid Full Text or citation:
Click here for full text options

Link to the External Link Resolver:
SFX Link

489.

Automated quality control for segmentation of myocardial perfusion SPECT.
Xu Y; Kavanagh P; Fish M; Gerlach J; Ramesh A; Lemley M; Hayes S; Berman DS; Germano G; Slomka PJ.
Ovid MEDLINE(R) and Epub Ahead of Print, In-Process & Other Non-Indexed Citations and Daily
Journal of Nuclear Medicine. 50(9):1418-26, 2009 Sep.
[Evaluation Study. Journal Article. Research Support, N.I.H., Extramural]
UI: 19690019
Authors Full Name
Xu, Yuan; Kavanagh, Paul; Fish, Mathews; Gerlach, James; Ramesh, Amit; Lemley, Mark; Hayes, Sean; Berman, Daniel S; Germano, Guido; Slomka, Piotr J.

Link to the Ovid Full Text or citation:
Click here for full text options

Link to the External Link Resolver:
SFX Link

490.

k-t PCA: temporally constrained k-t BLAST reconstruction using principal component analysis.
Pedersen H; Kozerke S; Ringgaard S; Nehrke K; Kim WY.
Ovid MEDLINE(R) and Epub Ahead of Print, In-Process & Other Non-Indexed Citations and Daily
Magnetic Resonance in Medicine. 62(3):706-16, 2009 Sep.
[Evaluation Study. Journal Article. Research Support, Non-U.S. Gov't]
UI: 19585603
Authors Full Name

Pedersen, Henrik; Kozerke, Sebastian; Ringgaard, Steffen; Nehrke, Kay; Kim, Won Yong.

Link to the Ovid Full Text or citation:
Click here for full text options

Link to the External Link Resolver:
SFX Link

491.

Correlation of automatic processing method in myocardial perfusion SPET with the interactive method.
Malek H; Bitarafan-Rajabi A; Rastgoo F; Yaghoobi N; Firoozabadi H.
Ovid MEDLINE(R) and Epub Ahead of Print, In-Process & Other Non-Indexed Citations and Daily
Hellenic Journal of Nuclear Medicine. 12(1):10-2, 2009 Jan-Apr.
[Journal Article]
UI: 19330174
Authors Full Name
Malek, Hadi; Bitarafan-Rajabi, Ahmad; Rastgoo, Fereidoon; Yaghoobi, Nahid; Firoozabadi, Hasan.

Link to the Ovid Full Text or citation:
Click here for full text options

Link to the External Link Resolver:
SFX Link

492.

Semi-automated assessment of left ventricular mass using transaxial Tc-99m Sestamibi SPECT imaging.
Rojas G; Raff U; Gonzalez P; Jaimovich R; Quintana JC.
Ovid MEDLINE(R) and Epub Ahead of Print, In-Process & Other Non-Indexed Citations and Daily
Computerized Medical Imaging & Graphics. 33(4):247-55, 2009 Jun.
[Evaluation Study. Journal Article. Research Support, Non-U.S. Gov't]
UI: 19261437
Authors Full Name
Rojas, G; Raff, U; Gonzalez, P; Jaimovich, R; Quintana, J C.

Link to the Ovid Full Text or citation:
Click here for full text options

Link to the External Link Resolver:
SFX Link

493.

Computer-assisted determination of left ventricular endocardial borders reduces variability in the echocardiographic assessment of ejection fraction.
Maret E; Brudin L; Lindstrom L; Nylander E; Ohlsson JL; Engvall JE.
Ovid MEDLINE(R) and Epub Ahead of Print, In-Process & Other Non-Indexed Citations and Daily
Cardiovascular Ultrasound. 6:55, 2008 Nov 11.
[Evaluation Study. Journal Article. Research Support, Non-U.S. Gov't]
UI: 19014461
Authors Full Name
Maret, Eva; Brudin, Lars; Lindstrom, Lena; Nylander, Eva; Ohlsson, Jan L; Engvall, Jan E.

Link to the Ovid Full Text or citation:
Click here for full text options

Link to the External Link Resolver:
SFX Link

494.

Automated cardiac motion compensation in PET/CT for accurate reconstruction of PET myocardial perfusion images.
Khurshid K; McGough RJ; Berger K.
Ovid MEDLINE(R) and Epub Ahead of Print, In-Process & Other Non-Indexed Citations and Daily
Physics in Medicine & Biology. 53(20):5705-18, 2008 Oct 21.
[Evaluation Study. Journal Article]
UI: 18812649
Authors Full Name
Khurshid, Khawar; McGough, Robert J; Berger, Kevin.

Link to the Ovid Full Text or citation:
Click here for full text options

Link to the External Link Resolver:
SFX Link

495.

Motion-compensated MR valve imaging with COMB tag tracking and super-resolution enhancement.
Dowsey AW; Keegan J; Lerotic M; Thom S; Firmin D; Yang GZ.
Ovid MEDLINE(R) and Epub Ahead of Print, In-Process & Other Non-Indexed Citations and Daily
Medical Image Analysis. 11(5):478-91, 2007 Oct.

[Journal Article. Research Support, Non-U.S. Gov't]
UI: 17804277
Authors Full Name
Dowsey, Andrew W; Keegan, Jennifer; Lerotic, Mirna; Thom, Simon; Firmin, David; Yang, Guang-Zhong.

Link to the Ovid Full Text or citation:
Click here for full text options

Link to the External Link Resolver:
SFX Link

496.

Methodology for quantifying absolute myocardial perfusion with PET and SPECT. [Review] [46 refs]
Lodge MA; Bengel FM.
Ovid MEDLINE(R) and Epub Ahead of Print, In-Process & Other Non-Indexed Citations and Daily
Current Cardiology Reports. 9(2):121-8, 2007 Apr.
[Journal Article. Review]
UI: 17430679
Authors Full Name
Lodge, Martin A; Bengel, Frank M.

Link to the Ovid Full Text or citation:
Click here for full text options

Link to the External Link Resolver:
SFX Link

497.

Subendocardial versus transmural ischaemia in myocardial perfusion SPECT--a Monte Carlo study.
Bartosik J; El-Ali HH; Nilsson U; Dahlstrom J; Edenbrandt L; Ljungberg M.
Ovid MEDLINE(R) and Epub Ahead of Print, In-Process & Other Non-Indexed Citations and Daily
Clinical Physiology & Functional Imaging. 26(6):343-50, 2006 Nov.
[Journal Article. Research Support, Non-U.S. Gov't]
UI: 17042900
Authors Full Name
Bartosik, Jolanta; El-Ali, Henrik Hussein; Nilsson, Ulf; Dahlstrom, Jan; Edenbrandt, Lars; Ljungberg, Michael.

Link to the Ovid Full Text or citation:
Click here for full text options

Link to the External Link Resolver:

SFX Link

498.

Automated quantification of the spatial extent of perfusion defects and viability on myocardial contrast echocardiography.
Micari A; Sklenar J; Belcik TA; Kaul S; Lindner JR.
Ovid MEDLINE(R) and Epub Ahead of Print, In-Process & Other Non-Indexed Citations and Daily
Journal of the American Society of Echocardiography. 19(4):379-85, 2006 Apr.
[Journal Article. Research Support, N.I.H., Extramural]
UI: 16581476
Authors Full Name
Micari, Antonio; Sklenar, Jiri; Belcik, Todd A; Kaul, Sanjiv; Lindner, Jonathan R.

Link to the Ovid Full Text or citation:
Click here for full text options

Link to the External Link Resolver:
SFX Link

499.

Evaluation of left ventricular ejection fraction by the quantitative algorithms QGS, ECTb, LMC and LVGTF using gated myocardial perfusion SPECT: investigation of relative accuracy.
Khalil MM; Elgazzar A; Khalil W.
Ovid MEDLINE(R) and Epub Ahead of Print, In-Process & Other Non-Indexed Citations and Daily
Nuclear Medicine Communications. 27(4):321-32, 2006 Apr.
[Comparative Study. Evaluation Study. Journal Article. Validation Study]
UI: 16531917
Title Comment
[Erratum in: Nucl Med Commun. 2006 Oct;27(10):831]
Authors Full Name
Khalil, Magdy Mohamed; Elgazzar, Abdelhamid; Khalil, Wafaa.

Link to the Ovid Full Text or citation:
Click here for full text options

Link to the External Link Resolver:
SFX Link

500.

Tissue-engineered vessel strengthens quickly under physiological deformation: application of a new perfusion bioreactor with machine vision.

Xu J; Ge H; Zhou X; Yang D; Guo T; He J; Li Q; Hao Z.
Ovid MEDLINE(R) and Epub Ahead of Print, In-Process & Other Non-Indexed Citations and Daily
Journal of Vascular Research. 42(6):503-8, 2005 Nov-Dec.
[Journal Article]
UI: 16155366
Authors Full Name
Xu, Jie; Ge, Haiyan; Zhou, Xiaolin; Yang, Daping; Guo, Tiefang; He, Jian; Li, Qing; Hao, Zhenhong.

Link to the Ovid Full Text or citation:
Click here for full text options

Link to the External Link Resolver:
SFX Link

501.

A new automated method for analysis of gated-SPECT images based on a three-dimensional heart shaped model.
Lomsky M; Richter J; Johansson L; El-Ali H; Astrom K; Ljungberg M; Edenbrandt L.
Ovid MEDLINE(R) and Epub Ahead of Print, In-Process & Other Non-Indexed Citations and Daily
Clinical Physiology & Functional Imaging. 25(4):234-40, 2005 Jul.
[Clinical Trial. Journal Article. Research Support, Non-U.S. Gov't]
UI: 15972026
Authors Full Name
Lomsky, Milan; Richter, Jens; Johansson, Lena; El-Ali, Henrik; Astrom, Karl; Ljungberg, Michael; Edenbrandt, Lars.

Link to the Ovid Full Text or citation:
Click here for full text options

Link to the External Link Resolver:
SFX Link

502.

[Myocardial perfusion scintigraphy with Tc-99m MIBI in patients with left bundle branch block: Visual quantification of the anteroseptal perfusion imaging for the diagnosis of left anterior descending artery stenosis]. [Afrikaans] Miokardiale perfusiebeelding met Tc-99m MIBI in pasiente met linker bondeltakblok: die visuele en kwantitatiewe beoordeling van anteroseptale perfusie vir die diagnose van stenose van die linker afdalende arterie. <Miokardiale perfusiebeelding met Tc-99m MIBI in pasiente met linker bondeltakblok: die visuele en kwantitatiewe beoordeling van anteroseptale perfusie vir die diagnose van stenose van die linker afdalende arterie.>
Moller J; Warwick J; Bouma H.
Ovid MEDLINE(R) and Epub Ahead of Print, In-Process & Other Non-Indexed Citations and Daily

Cardiovascular Journal of Southern Africa. 16(2):95-101, 2005 Mar-Apr.
[Journal Article. Validation Study]
UI: 15915276
Authors Full Name
Moller, J; Warwick, J; Bouma, H.

Link to the Ovid Full Text or citation:
Click here for full text options

Link to the External Link Resolver:
SFX Link

503.

An observer study methodology for evaluating detection of motion abnormalities in gated myocardial perfusion SPECT.
Lalush DS; Jatko MK; Segars WP.
Ovid MEDLINE(R) and Epub Ahead of Print, In-Process & Other Non-Indexed Citations and Daily
IEEE Transactions on Biomedical Engineering. 52(3):480-5, 2005 Mar.
[Evaluation Study. Journal Article. Research Support, U.S. Gov't, P.H.S.. Validation Study]
UI: 15759578
Authors Full Name
Lalush, David S; Jatko, Megan K; Segars, W Paul.

Link to the Ovid Full Text or citation:
Click here for full text options

Link to the External Link Resolver:
SFX Link

504.

Automated quantification of myocardial perfusion SPECT using simplified normal limits.
Slomka PJ; Nishina H; Berman DS; Akincioglu C; Abidov A; Friedman JD; Hayes SW; Germano G.
Ovid MEDLINE(R) and Epub Ahead of Print, In-Process & Other Non-Indexed Citations and Daily
Journal of Nuclear Cardiology. 12(1):66-77, 2005 Jan-Feb.
[Clinical Trial. Controlled Clinical Trial. Journal Article]
UI: 15682367
Title Comment
[Comment in: J Nucl Cardiol. 2005 Jan-Feb;12(1):3-4; PMID: 15682358 [https://www.ncbi.nlm.nih.gov/pubmed/15682358]]
Authors Full Name
Slomka, Piotr J; Nishina, Hidetaka; Berman, Daniel S; Akincioglu, Cigdem; Abidov, Aiden; Friedman, John D; Hayes, Sean W; Germano, Guido.

Link to the Ovid Full Text or citation:
Click here for full text options

Link to the External Link Resolver:
SFX Link

505.

[1-(11)C]Acetate as a quantitative perfusion tracer in myocardial PET.
van den Hoff J; Burchert W; Borner AR; Fricke H; Kuhnel G; Meyer GJ; Otto D; Weckesser E; Wolpers HG; Knapp WH.
Ovid MEDLINE(R) and Epub Ahead of Print, In-Process & Other Non-Indexed Citations and Daily
Journal of Nuclear Medicine. 42(8):1174-82, 2001 Aug.
[Clinical Trial. Comparative Study. Journal Article]
UI: 11483676
Title Comment
[Comment in: J Nucl Med. 2001 Aug;42(8):1183-4; PMID: 11483677 [https://www.ncbi.nlm.nih.gov/pubmed/11483677]]
Authors Full Name
van den Hoff, J; Burchert, W; Borner, A R; Fricke, H; Kuhnel, G; Meyer, G J; Otto, D; Weckesser, E; Wolpers, H G; Knapp, W H.

Link to the Ovid Full Text or citation:
Click here for full text options

Link to the External Link Resolver:
SFX Link

506.

A knowledge discovery approach to diagnosing myocardial perfusion.
Cios KJ; Teresinska A; Konieczna S; Potocka J; Sharma S.
Ovid MEDLINE(R) and Epub Ahead of Print, In-Process & Other Non-Indexed Citations and Daily
IEEE Engineering in Medicine & Biology Magazine. 19(4):17-25, 2000 Jul-Aug.
[Journal Article. Research Support, Non-U.S. Gov't]
UI: 10916729
Authors Full Name
Cios, K J; Teresinska, A; Konieczna, S; Potocka, J; Sharma, S.

Link to the Ovid Full Text or citation:
Click here for full text options

Link to the External Link Resolver:
SFX Link

507.

Bradykinin modulation of isolated rabbit heart function is mediated by intrinsic cardiac neurons.
Izrailtyan I; Kresh JY.
Ovid MEDLINE(R) and Epub Ahead of Print, In-Process & Other Non-Indexed Citations and Daily
Cardiovascular Research. 33(3):641-9, 1997 Mar.
[Journal Article. Research Support, Non-U.S. Gov't]
UI: 9093534
Authors Full Name
Izrailtyan, I; Kresh, J Y.

Link to the Ovid Full Text or citation:
Click here for full text options

Link to the External Link Resolver:
SFX Link

508.

Feasibility analysis of a case-based reasoning system for automated detection of coronary heart disease from myocardial scintigrams.
Haddad M; Adlassnig KP; Porenta G.
Ovid MEDLINE(R) and Epub Ahead of Print, In-Process & Other Non-Indexed Citations and Daily
Artificial Intelligence in Medicine. 9(1):61-78, 1997 Jan.
[Journal Article]
UI: 9021059
Authors Full Name
Haddad, M; Adlassnig, K P; Porenta, G.

Link to the Ovid Full Text or citation:
Click here for full text options

Link to the External Link Resolver:
SFX Link
