# Supplementary material for: Deep learning applications in myocardial perfusion imaging, a systematic review and meta-analysis
Source: Inform Med Unlocked. 2022;32:101055. doi: 10.1016/j.imu.2022.101055 (PMC9514037; doi:10.1016/j.imu.2022.101055)
Supplement: Multimedia component 2 [file mmc2.pdf]

Table (2): List of all studies with their corresponding risk of bias assessments.

+ No risk of bias

? Equivocal risk

- High risk of bias

Study	Patient selection	Index test	Reference test	Index test results blinded	Reference test results blinded
Fujita 1992	?	+	+	?	?
Wang 1993	+	+	+	?	?
Porenta 1994	+	+	+	-	?
Hamilton 1994	+	-	+	-	?
Goodenday 1997	+	+	+	?	?
Lindahl 1997	+	+	+	+	?
Scott 2004	+	+	+	+	+
Ohlsson 2004	+	-	+	-	?
Tagil 2008	+	+	+	+	+
Lomsky 2008	+	+	+	?	+
Guner 2010	+	+	+	+	+
Abbasi 2012	+	-	+	-	?
Arsanjani 2013	+	+	+	+	?
Nakajima 2015	+	+	+	+	?
Xiong 2015	+	+	+	-	?
Parages 2016	?	+	+	?	?
Lee 2016	+	+	+	?	?
Li 2017	+	+	+	?	?
Kim 2017	+	+	+	?	?
Nakajima 2017	+	+	+	-	+
Al Mallah 2017	+	+	+	?	?
Nakajima 2018	+	+	+	-	+
Hung 2018	+	-	+	-	?
Eisenberg 2018	+	+	+	?	?

Betancur 2019	+	+	+	+	+
Kim 2019	+	+	+	?	?
Scannell 2019	+	-	+	-	+
Spire 2019	?	-	+	-	+
Fan 2019	+	+	+	+	+
Ko 2019	+	-	+	-	?
Chiu 2019	+	+	+	?	?
Song 2019	?	+	+	?	?
Rahmani 2019	+	-	+	-	?
Knott 2020	+	-	+	-	+
Shiri 2020	+	-	+	-	?
Singh 2020	+	+	+	?	?
Ramon 2020	+	-	+	-	?
Apostolopoulos 2020	+	+	+	?	?
Xue 2020	+	-	+	-	?
Berkaya 2020	+	-	+	-	?
Shi 2020	+	-	+	-	?
Hu 2020	+	+	+	+	+
Juarez-Orozco 2020	+	-	+	-	+
Shu 2020	+	+	+	+	+
Cantoni 2020	+	+	+	?	?
Wang 2020	+	+	+	-	+
