# Supplementary material for: Deep learning applications in myocardial perfusion imaging, a systematic review and meta-analysis
Source: Inform Med Unlocked. 2022;32:101055. doi: 10.1016/j.imu.2022.101055 (PMC9514037; doi:10.1016/j.imu.2022.101055)
Supplement: Multimedia component 3 [file mmc3.pdf]

SR perfusion

This is an R Markdown Notebook. When you execute code within the notebook, the results appear beneath the code.

Try executing this chunk by clicking the *Run* button within the chunk or by placing your cursor inside it and pressing *Cmd+Shift+Enter*.

```
Audit <- data.frame(TP = c(104, 67, 75, 359, 80, 375, 559, 41, 74, 474, 381,
114, 132), FN = c(41, 20, 15, 44, 33, 72, 251, 17, 10, 244, 62, 38, 18), FP =
c(1, 14, 50, 22, 62, 61, 132, 26, 1, 75, 10, 17, 0), TN = c(3, 34, 176, 525,
133, 449, 215, 56, 21, 367, 493, 47, 42))
Audit
```

```
##     TP  FN  FP  TN
## 1  104  41   1   3
## 2   67  20  14  34
## 3   75  15  50 176
## 4  359  44  22 525
## 5   80  33  62 133
## 6  375  72  61 449
## 7  559 251 132 215
## 8   41  17  26  56
## 9   74  10   1  21
## 10 474 244  75 367
## 11 381  62  10 493
## 12 114  38  17  47
## 13 132  18   0  42
```

```
Audit$names <- c("Porenta 1994", "Lindahl 1997", "Tagil 2008", "Lomsky 2008",
"Guner 2010", "Arsanjani 2013", "Nakajima 2015", "Xiong 2015", "Nakajima
2018", "Betancur 2019", "Spier 2019", "Apostolopoulos 2020", "Berkaya 2020")
library("mada")
```

```
## Loading required package: mvtnorm

## Loading required package: ellipse

##
## Attaching package: 'ellipse'

## The following object is masked from 'package:graphics':
##
##     pairs

## Loading required package: mvmeta

## This is mvmeta 1.0.3. For an overview type: help('mvmeta-package').

## Loading required package: metafor
```

```
## Loading required package: Matrix

## Loading required package: metadat

## Warning: package 'metadat' was built under R version 4.0.5

##
## Loading the 'metafor' package (version 3.4-0). For an
## introduction to the package please type: help(metafor)

##
## Attaching package: 'metafor'

## The following object is masked from 'package:mvmeta':
##
##     blup

##
## Attaching package: 'mada'

## The following object is masked from 'package:metafor':
##
##     forest

madad(Audit)

## Descriptive summary of Audit with 13 primary studies.
## Confidence level for all calculations set to 95 %
## Using a continuity correction of 0.5 if applicable
##
## Diagnostic accuracies
##                       sens  2.5% 97.5%  spec  2.5% 97.5%
## Porenta 1994        0.716 0.638 0.783 0.700 0.299 0.927
## Lindahl 1997        0.767 0.669 0.843 0.704 0.565 0.813
## Tagil 2008          0.830 0.739 0.893 0.778 0.719 0.827
## Lomsky 2008         0.890 0.856 0.917 0.959 0.939 0.973
## Guner 2010          0.706 0.617 0.782 0.681 0.613 0.742
## Arsanjani 2013      0.838 0.801 0.869 0.880 0.849 0.905
## Nakajima 2015       0.690 0.657 0.721 0.619 0.567 0.669
## Xiong 2015          0.703 0.577 0.805 0.681 0.574 0.771
## Nakajima 2018       0.876 0.790 0.931 0.935 0.760 0.985
## Betancur 2019       0.660 0.625 0.694 0.830 0.792 0.862
## Spier 2019          0.859 0.824 0.889 0.979 0.963 0.988
## Apostolopoulos 2020 0.748 0.674 0.810 0.731 0.612 0.823
## Berkaya 2020        0.877 0.816 0.921 0.988 0.898 0.999
##
## Test for equality of sensitivities:
## X-squared = 164.2997, df = 12, p-value = <2e-16
## Test for equality of specificities:
## X-squared = 349.1279, df = 12, p-value = <2e-16
##
##
```

```
## Diagnostic OR and likelihood ratios
##                         DOR    2.5%     97.5%  posLR   2.5%    97.5% negLR
## Porenta 1994          5.876   0.839    41.142  2.386  0.623    9.137 0.406
## Lindahl 1997          7.834   3.564    17.224  2.592  1.658    4.053 0.331
## Tagil 2008           17.024   9.070    31.956  3.729  2.874    4.839 0.219
## Lomsky 2008         188.681 111.676   318.784 21.673 14.440   32.529 0.115
## Guner 2010            5.133   3.105     8.485  2.214  1.748    2.805 0.431
## Arsanjani 2013       37.855  26.243    54.606  6.964  5.490    8.835 0.184
## Nakajima 2015         3.618   2.783     4.705  1.812  1.573    2.088 0.501
## Xiong 2015            5.056   2.450    10.435  2.203  1.544    3.142 0.436
## Nakajima 2018       101.698  17.205   601.138 13.439  2.854   63.274 0.132
## Betancur 2019         9.446   7.056    12.647  3.872  3.132    4.787 0.410
## Spier 2019          286.888 147.213   559.086 41.243 22.641   75.128 0.144
## Apostolopoulos 2020   8.072   4.178    15.596  2.780  1.843    4.192 0.344
## Berkaya 2020        608.784  35.919 10318.024 75.464  4.794 1187.897 0.124
##                      2.5% 97.5%
## Porenta 1994        0.217 0.762
## Lindahl 1997        0.217 0.504
## Tagil 2008          0.138 0.347
## Lomsky 2008         0.087 0.152
## Guner 2010          0.320 0.583
## Arsanjani 2013      0.149 0.228
## Nakajima 2015       0.439 0.571
## Xiong 2015          0.286 0.663
## Nakajima 2018       0.074 0.235
## Betancur 2019       0.367 0.458
## Spier 2019          0.114 0.181
## Apostolopoulos 2020 0.252 0.470
## Berkaya 2020        0.081 0.190
##
## Correlation of sensitivities and false positive rates:
##    rho  2.5 % 97.5 %
## -0.827 -0.947 -0.508
```

```
forest(madad(Audit), type="sens", snames=Audit$names, col="blue")
```

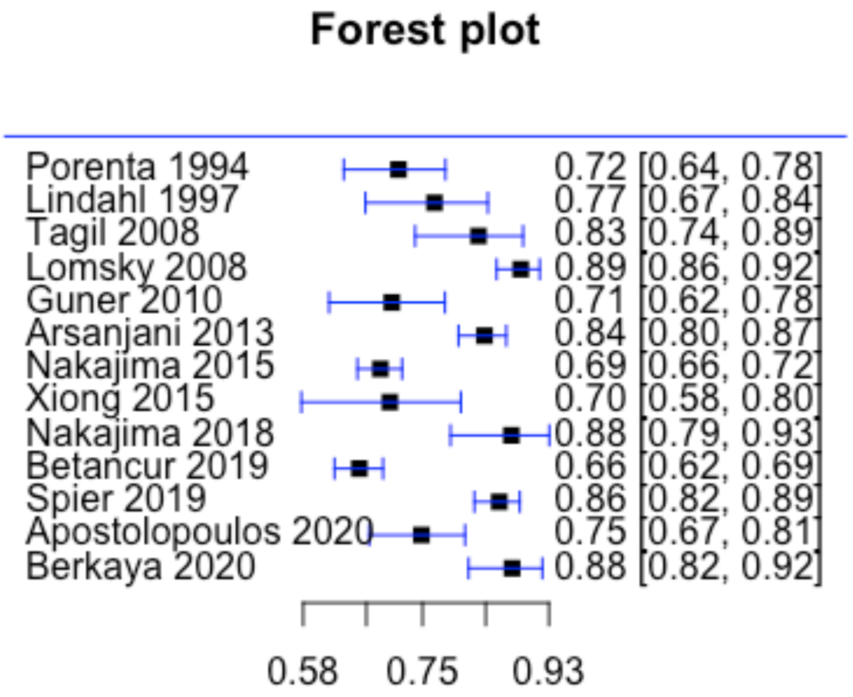


```
forest(madad(Audit), type="spec", snames=Audit$names, col="blue")
```

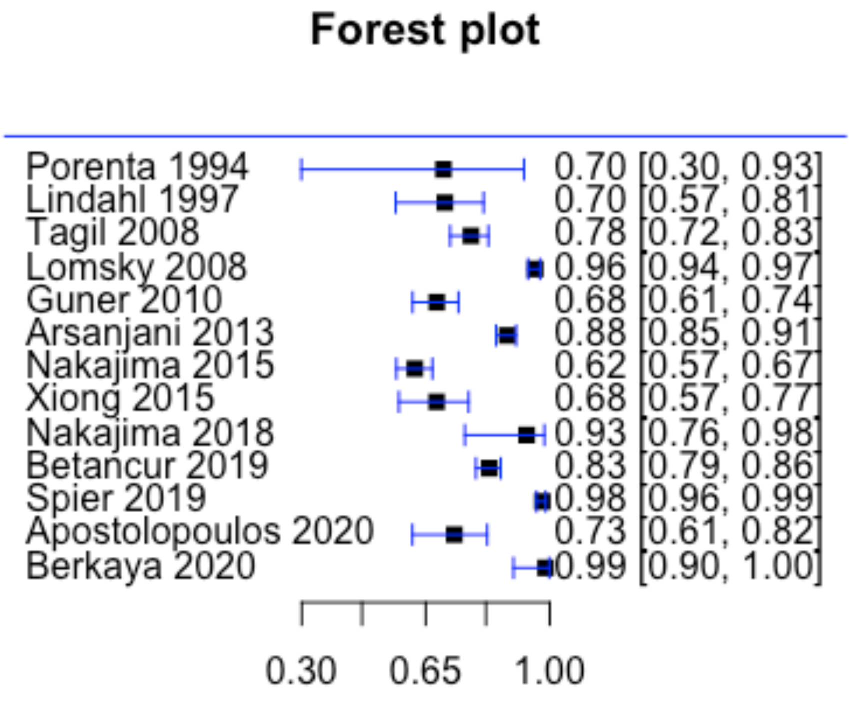


```
'comparing SROC for CNN and MLP'

## [1] "comparing SROC for CNN and MLP"

CNN <- data.frame(TP= c(474, 381, 114, 132), FN = c(244, 62, 38, 18), FP =
c(75, 10, 17, 0), TN = c(367, 493, 47, 42))
MLP <- data.frame(TP= c(104, 67, 75, 359, 80, 559, 74), FN = c(41, 20, 15,
44, 33, 251, 10), FP = c(1, 14, 50, 22, 62, 132, 1), TN = c(3, 34, 176, 525,
133, 215, 21))
fit.Audit <- reitsma((Audit))
fit.CNN <- reitsma(CNN)

## Warning in checkdata(freqdata): There are very few primary studies!

fit.MLP <- reitsma(MLP)
plot(fit.CNN, xlim=c(0, .5), ylim=c(.5, 1), col="red", main="Comparison of
CNN and MLP")
lines(sroc(fit.MLP), lty=2)
ROCellipse(fit.MLP, lty=2, pch=2, col="blue", add=TRUE)
points(fpr(CNN), sens(CNN), cex=.5, col="red")
points(fpr(MLP), sens(MLP), cex=.5, pch=2, col="blue")
legend("bottomright", c("CNN", "MLP"), pch=1:2, lty=1:2, col = c("red",
"blue"))
```

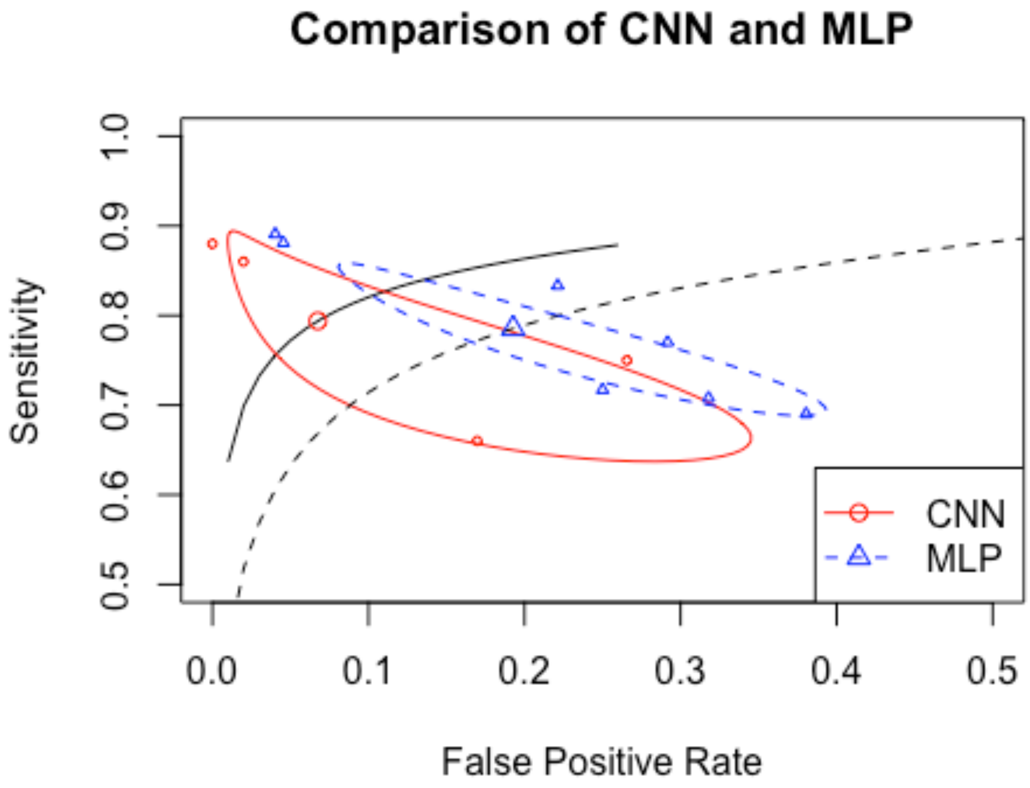


```
summary(fit.Audit)

## Call:  reitsma.default(data = (Audit))
##
## Bivariate diagnostic random-effects meta-analysis
## Estimation method: REML
##
## Fixed-effects coefficients
##                     Estimate Std. Error      z Pr(>|z|) 95%ci.lb 95%ci.ub
## tsens.(Intercept)     1.304      0.147  8.860    0.000    1.016    1.593
***
## tfpr.(Intercept)     -1.721      0.324 -5.310    0.000   -2.357   -1.086
***
## sensitivity           0.787          -      -        -    0.734    0.831
## false pos. rate       0.152          -      -        -    0.087    0.252
## ---
## Signif. codes:  0 '***' 0.001 '**' 0.01 '*' 0.05 '.' 0.1 ' ' 1
##
## Variance components: between-studies Std. Dev and correlation matrix
##       Std. Dev  tsens  tfpr
## tsens    0.494  1.000     .
## tfpr     1.107 -0.853 1.000
##
```

```
##  logLik     AIC     BIC
##  28.194 -46.387 -40.097
##
## AUC:  0.859
## Partial AUC (restricted to observed FPRs and normalized):  0.777
##
## I2 estimates
## Zhou and Dendukuri approach:  29.4 %
## Holling sample size unadjusted approaches:  90.9 - 96.9 %
## Holling sample size adjusted approaches:  10.1 - 11.3 %
```

```
summary(fit.CNN)
```

```
## Call:  reitsma.default(data = CNN)
##
## Bivariate diagnostic random-effects meta-analysis
## Estimation method: REML
##
## Fixed-effects coefficients
##                    Estimate Std. Error      z Pr(>|z|) 95%ci.lb 95%ci.ub
## tsens.(Intercept)     1.349      0.321  4.200    0.000    0.719    1.978
***
## tfpr.(Intercept)     -2.625      0.811 -3.236    0.001   -4.215   -1.035
**
## sensitivity           0.794          -      -        -    0.672    0.878
## false pos. rate       0.068          -      -        -    0.015    0.262
## ---
## Signif. codes:  0 '***' 0.001 '**' 0.01 '*' 0.05 '.' 0.1 ' ' 1
##
## Variance components: between-studies Std. Dev and correlation matrix
##        Std. Dev  tsens  tfpr
## tsens     0.620  1.000     .
## tfpr      1.552 -0.907 1.000
##
##  logLik     AIC     BIC
##  11.393 -12.786 -12.388
##
## AUC:  0.894
## Partial AUC (restricted to observed FPRs and normalized):  0.816
##
## I2 estimates
## Zhou and Dendukuri approach:  60.2 %
## Holling sample size unadjusted approaches:  95.3 - 98.4 %
## Holling sample size adjusted approaches:  12.6 - 14.6 %
```

```
summary(fit.MLP)
```

```
## Call:  reitsma.default(data = MLP)
##
## Bivariate diagnostic random-effects meta-analysis
## Estimation method: REML
```

```
##
## Fixed-effects coefficients
##                     Estimate Std. Error      z Pr(>|z|) 95%ci.lb 95%ci.ub
## tsens.(Intercept)    1.297      0.207  6.279    0.000    0.892    1.702
***
## tfpr.(Intercept)    -1.433      0.408 -3.513    0.000   -2.232   -0.633
***
## sensitivity          0.785          -      -        -    0.709    0.846
## false pos. rate      0.193          -      -        -    0.097    0.347
## ---
## Signif. codes:  0 '***' 0.001 '**' 0.01 '*' 0.05 '.' 0.1 ' ' 1
##
## Variance components: between-studies Std. Dev and correlation matrix
##       Std. Dev  tsens  tfpr
## tsens    0.515  1.000     .
## tfpr     1.032 -1.000 1.000
##
##  logLik     AIC     BIC
##  16.909 -23.818 -20.623
##
## AUC:  0.848
## Partial AUC (restricted to observed FPRs and normalized):  0.77
##
## I2 estimates
## Zhou and Dendukuri approach:  0 %
## Holling sample size unadjusted approaches:  91.2 - 97.4 %
## Holling sample size adjusted approaches:  14.8 - 16.2 %
```

```
'Publication bias'
```

```
## [1] "Publication bias"
```

```
library(tidyverse)
```

```
## ── Attaching packages ─────────────────────────────────────── tidyverse
1.3.0 ──

## ✓ ggplot2 3.3.5     ✓ purrr   0.3.4
## ✓ tibble  3.1.0     ✓ dplyr   1.0.5
## ✓ tidyr   1.1.3     ✓ stringr 1.4.0
## ✓ readr   1.4.0     ✓ forcats 0.5.1

## ── Conflicts ──────────────────────────────────────────
tidyverse_conflicts() ──
## x tidyr::expand() masks Matrix::expand()
## x dplyr::filter() masks stats::filter()
## x dplyr::lag()    masks stats::lag()
## x tidyr::pack()   masks Matrix::pack()
## x readr::spec()   masks mada::spec()
## x tidyr::unpack() masks Matrix::unpack()
```

```
library(meta)

## Loading 'meta' package (version 5.5-0).
## Type 'help(meta)' for a brief overview.
## Readers of 'Meta-Analysis with R (Use R!)' should install
## older version of 'meta' package: https://tinyurl.com/dt4y5drs

##
## Attaching package: 'meta'

## The following object is masked from 'package:mada':
##
##     forest

Audit$acc <- c(0.718, 0.748, 0.794, 0.931,  0.692, 0.861, 0.669, 0.693,
0.896, 0.725, 0.924, 0.745, 0.906)
mean(Audit$acc)

## [1] 0.7924615

Audit$se <- sd(Audit$acc)/sqrt(Audit$acc)
Audit$ez <- Audit$acc / mean(Audit$acc)
Audit

##     TP  FN  FP  TN                names   acc        se        ez
## 1  104  41   1   3        Porenta 1994 0.718 0.1152080 0.9060377
## 2   67  20  14  34        Lindahl 1997 0.748 0.1128741 0.9438944
## 3   75  15  50 176          Tagil 2008 0.794 0.1095556 1.0019414
## 4  359  44  22 525         Lomsky 2008 0.931 0.1011743 1.1748204
## 5   80  33  62 133          Guner 2010 0.692 0.1173524 0.8732285
## 6  375  72  61 449      Arsanjani 2013 0.861 0.1052067 1.0864881
## 7  559 251 132 215       Nakajima 2015 0.669 0.1193526 0.8442050
## 8   41  17  26  56         Xiong 2015 0.693 0.1172677 0.8744904
## 9   74  10   1  21       Nakajima 2018 0.896 0.1031314 1.1306542
## 10 474 244  75 367       Betancur 2019 0.725 0.1146505 0.9148709
## 11 381  62  10 493          Spier 2019 0.924 0.1015568 1.1659872
## 12 114  38  17  47 Apostolopoulos 2020 0.745 0.1131011 0.9401087
## 13 132  18   0  42        Berkaya 2020 0.906 0.1025607 1.1432732

m.gen <- metagen(TE= Audit$ez, seTE=Audit$se, studlab = Audit$names)
funnel.meta(m.gen, xlim=c(0.6, 1.3), studlab=FALSE)
```

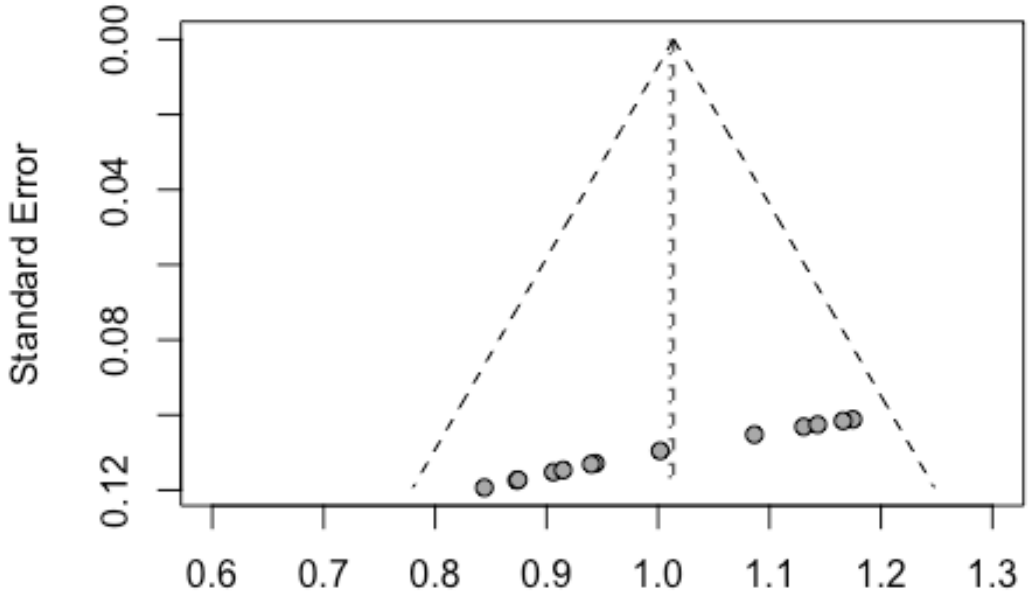


```
col.contour = c("gray75", "gray85", "gray95")
funnel.meta(m.gen, xlim=c(0.6, 1.4), contour=c(0.9, 0.95, 0.99),
col.contour=col.contour, sutdlab=FALSE)
legend(x=1.2, y=0.01, legend=c("p < 0.1", "p < 0.05", "p < 0.01"), fill =
col.contour)
title("Funnel Plot (MPS perfusion classification)")
```

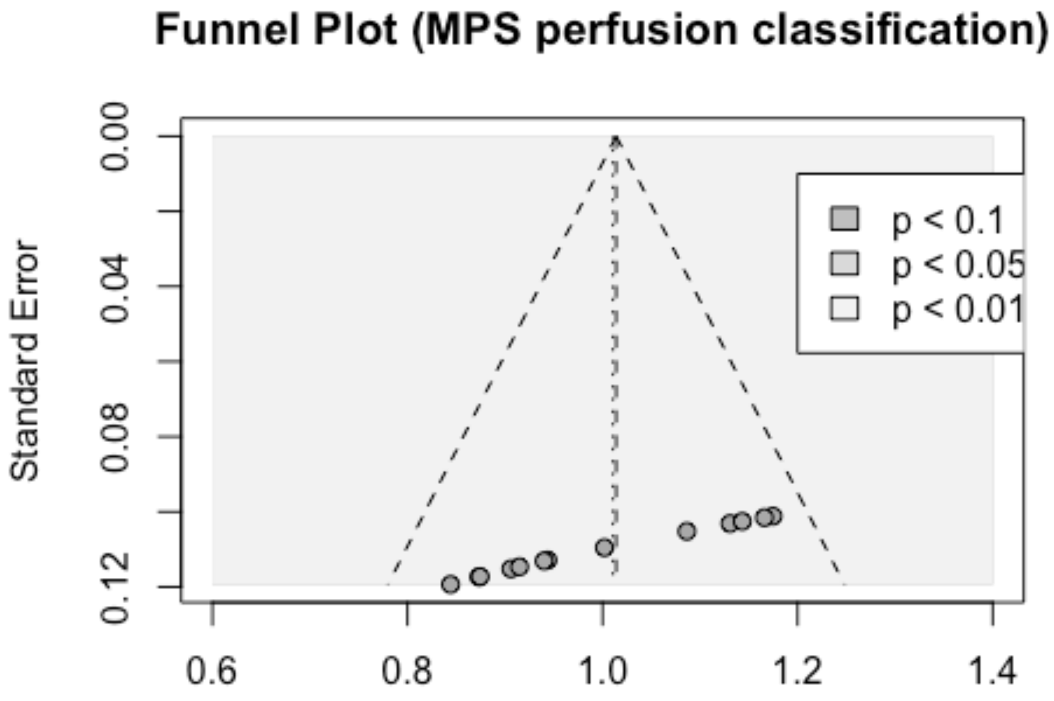


```
'Eggers test'

## [1] "Eggers test"

m.gen$data %>% mutate(y=Audit$ez/Audit$se, x = 1/Audit$se) %>% lm(y ~ x,
data=.) %>% summary()

##
## Call:
## lm(formula = y ~ x, data = .)
##
## Residuals:
##      Min       1Q   Median       3Q      Max
## -0.09601 -0.05997 -0.00595  0.04226  0.10831
##
## Coefficients:
##             Estimate Std. Error t value Pr(>|t|)
## (Intercept) -18.4937     0.3027  -61.09  2.8e-15 ***
## x             3.0385     0.0332   91.53  < 2e-16 ***
## ---
## Signif. codes:  0 '***' 0.001 '**' 0.01 '*' 0.05 '.' 0.1 ' ' 1
##
## Residual standard error: 0.06422 on 11 degrees of freedom
```

```
## Multiple R-squared:  0.9987, Adjusted R-squared:  0.9986 
## F-statistic:  8378 on 1 and 11 DF,  p-value: < 2.2e-16
```

```
'Test heterogeneity'
```

```
## [1] "Test heterogeneity"
```

```
m.gen <- update.meta(m.gen, prediction = TRUE)
summary(m.gen)
```

```
##                                      95%-CI %W(common) %W(random)
## Porenta 1994        0.9060 [0.6802; 1.1318]        7.0        7.1
## Lindahl 1997        0.9439 [0.7227; 1.1651]        7.3        7.4
## Tagil 2008          1.0019 [0.7872; 1.2167]        7.7        7.7
## Lomsky 2008         1.1748 [0.9765; 1.3731]        9.0        8.7
## Guner 2010          0.8732 [0.6432; 1.1032]        6.7        6.9
## Arsanjani 2013      1.0865 [0.8803; 1.2927]        8.4        8.2
## Nakajima 2015       0.8442 [0.6103; 1.0781]        6.5        6.8
## Xiong 2015          0.8745 [0.6446; 1.1043]        6.7        6.9
## Nakajima 2018       1.1307 [0.9285; 1.3328]        8.7        8.5
## Betancur 2019       0.9149 [0.6902; 1.1396]        7.0        7.2
## Spier 2019          1.1660 [0.9669; 1.3650]        9.0        8.7
## Apostolopoulos 2020 0.9401 [0.7184; 1.1618]        7.2        7.4
## Berkaya 2020        1.1433 [0.9423; 1.3443]        8.8        8.5
## 
## Number of studies combined: k = 13
## 
##                                       95%-CI     z  p-value
## Common effect model  1.0140 [0.9544; 1.0736] 33.34 < 0.0001
## Random effects model 1.0107 [0.9425; 1.0789] 29.05 < 0.0001
## Prediction interval         [0.8569; 1.1645]                
## 
## Quantifying heterogeneity:
##  tau^2 = 0.0037 [0.0000; 0.0295]; tau = 0.0606 [0.0000; 0.1717]
##  I^2 = 22.3% [0.0%; 59.4%]; H = 1.13 [1.00; 1.57]
## 
## Test of heterogeneity:
##      Q d.f. p-value
##  15.44   12  0.2184
## 
## Details on meta-analytical method:
## - Inverse variance method
## - Restricted maximum-likelihood estimator for tau^2
## - Q-Profile method for confidence interval of tau^2 and tau
```

Add a new chunk by clicking the *Insert Chunk* button on the toolbar or by pressing *Cmd+Option+I*.

When you save the notebook, an HTML file containing the code and output will be saved alongside it (click the *Preview* button or press *Cmd+Shift+K* to preview the HTML file).

The preview shows you a rendered HTML copy of the contents of the editor. Consequently, unlike *Knit*, *Preview* does not run any R code chunks. Instead, the output of the chunk when it was last run in the editor is displayed.
